# Regulatory T Cell Responses in Participants with Type 1 Diabetes after a Single Dose of Interleukin-2: A Non-Randomised, Open Label, Adaptive Dose-Finding Trial

**DOI:** 10.1371/journal.pmed.1002139

**Published:** 2016-10-11

**Authors:** John A. Todd, Marina Evangelou, Antony J. Cutler, Marcin L. Pekalski, Neil M. Walker, Helen E. Stevens, Linsey Porter, Deborah J. Smyth, Daniel B. Rainbow, Ricardo C. Ferreira, Laura Esposito, Kara M. D. Hunter, Kevin Loudon, Kathryn Irons, Jennie H. Yang, Charles J. M. Bell, Helen Schuilenburg, James Heywood, Ben Challis, Sankalpa Neupane, Pamela Clarke, Gillian Coleman, Sarah Dawson, Donna Goymer, Katerina Anselmiova, Jane Kennet, Judy Brown, Sarah L. Caddy, Jia Lu, Jane Greatorex, Ian Goodfellow, Chris Wallace, Tim I. Tree, Mark Evans, Adrian P. Mander, Simon Bond, Linda S. Wicker, Frank Waldron-Lynch

**Affiliations:** 1 JDRF/Wellcome Trust Diabetes and Inflammation Laboratory, Department of Medical Genetics, Cambridge Institute for Medical Research, National Institute for Health Research Cambridge Biomedical Research Centre, Cambridge Biomedical Campus, University of Cambridge, Cambridge, United Kingdom; 2 Department of Mathematics, Imperial College London, London, United Kingdom; 3 National Institute for Health Research Cambridge Clinical Trials Unit, Cambridge University Hospitals NHS Foundation Trust, Cambridge Biomedical Campus, Cambridge, United Kingdom; 4 Department of Immunobiology, Faculty of Life Sciences & Medicine, King’s College London, National Institute of Health Research Biomedical Research Centre, Guy’s and St Thomas’ National Health Service Foundation Trust and King’s College London, London, United Kingdom; 5 Wellcome Trust/MRC Institute of Metabolic Science, National Institute for Health Research Cambridge Biomedical Research Centre, Cambridge Biomedical Campus, Cambridge, United Kingdom; 6 Division of Virology, Department of Pathology, Addenbrooke’s Hospital, Cambridge Biomedical Campus, University of Cambridge, Cambridge, United Kingdom; 7 Public Health England, Clinical Microbiology and Public Health Laboratory, Addenbrooke’s Hospital, Cambridge University Hospitals NHS Foundation Trust, Cambridge Biomedical Campus, Cambridge, United Kingdom; 8 MRC Biostatistics Unit Hub for Trials Methodology Research, Cambridge Institute of Public Health, Cambridge Biomedical Campus, Cambridge, United Kingdom; Leiden University Medical Centre, NETHERLANDS

## Abstract

**Background:**

Interleukin-2 (IL-2) has an essential role in the expansion and function of CD4^+^ regulatory T cells (Tregs). Tregs reduce tissue damage by limiting the immune response following infection and regulate autoreactive CD4^+^ effector T cells (Teffs) to prevent autoimmune diseases, such as type 1 diabetes (T1D). Genetic susceptibility to T1D causes alterations in the IL-2 pathway, a finding that supports Tregs as a cellular therapeutic target. Aldesleukin (Proleukin; recombinant human IL-2), which is administered at high doses to activate the immune system in cancer immunotherapy, is now being repositioned to treat inflammatory and autoimmune disorders at lower doses by targeting Tregs.

**Methods and Findings:**

To define the aldesleukin dose response for Tregs and to find doses that increase Tregs physiologically for treatment of T1D, a statistical and systematic approach was taken by analysing the pharmacokinetics and pharmacodynamics of single doses of subcutaneous aldesleukin in the Adaptive Study of IL-2 Dose on Regulatory T Cells in Type 1 Diabetes (DILT1D), a single centre, non-randomised, open label, adaptive dose-finding trial with 40 adult participants with recently diagnosed T1D. The primary endpoint was the maximum percentage increase in Tregs (defined as CD3^+^CD4^+^CD25^high^CD127^low^) from the baseline frequency in each participant measured over the 7 d following treatment. There was an initial learning phase with five pairs of participants, each pair receiving one of five pre-assigned single doses from 0.04 × 10^6^ to 1.5 × 10^6^ IU/m^2^, in order to model the dose-response curve. Results from each participant were then incorporated into interim statistical modelling to target the two doses most likely to induce 10% and 20% increases in Treg frequencies. Primary analysis of the evaluable population (*n* = 39) found that the optimal doses of aldesleukin to induce 10% and 20% increases in Tregs were 0.101 × 10^6^ IU/m^2^ (standard error [SE] = 0.078, 95% CI = −0.052, 0.254) and 0.497 × 10^6^ IU/m^2^ (SE = 0.092, 95% CI = 0.316, 0.678), respectively. On analysis of secondary outcomes, using a highly sensitive IL-2 assay, the observed plasma concentrations of the drug at 90 min exceeded the hypothetical Treg-specific therapeutic window determined in vitro (0.015–0.24 IU/ml), even at the lowest doses (0.040 × 10^6^ and 0.045 × 10^6^ IU/m^2^) administered. A rapid decrease in Treg frequency in the circulation was observed at 90 min and at day 1, which was dose dependent (mean decrease 11.6%, SE = 2.3%, range 10.0%–48.2%, *n* = 37), rebounding at day 2 and increasing to frequencies above baseline over 7 d. Teffs, natural killer cells, and eosinophils also responded, with their frequencies rapidly and dose-dependently decreased in the blood, then returning to, or exceeding, pretreatment levels. Furthermore, there was a dose-dependent down modulation of one of the two signalling subunits of the IL-2 receptor, the β chain (CD122) (mean decrease = 58.0%, SE = 2.8%, range 9.8%–85.5%, *n* = 33), on Tregs and a reduction in their sensitivity to aldesleukin at 90 min and day 1 and 2 post-treatment. Due to blood volume requirements as well as ethical and practical considerations, the study was limited to adults and to analysis of peripheral blood only.

**Conclusions:**

The DILT1D trial results, most notably the early altered trafficking and desensitisation of Tregs induced by a single ultra-low dose of aldesleukin that resolves within 2–3 d, inform the design of the next trial to determine a repeat dosing regimen aimed at establishing a steady-state Treg frequency increase of 20%–50%, with the eventual goal of preventing T1D.

**Trial Registration:**

ISRCTN Registry ISRCTN27852285; ClinicalTrials.gov NCT01827735

## Abstract 2

Frank Waldron-Lynch and colleagues investigate immune cell biomarker responses in patients with type 1 diabetes following a single dose of recombinant interleukin-2.

## Author Summary

**Why Was This Study Done?:**

- Insulin replacement, the only approved therapy for the treatment of type 1 diabetes (T1D), treats the symptoms but not the underlying cause of the disease, namely, immune-mediated destruction of the insulin-producing β cells of the pancreas.
- Aldesleukin, recombinant interleukin-2 (IL-2), was selected as a candidate immunotherapy to prevent or delay autoimmunity because previous genetic and phenotypic analyses indicate a major role for the IL-2 pathway in the development of T1D.
- IL-2 is critical for maintaining the function of the regulatory T cells (Tregs) that prevent autoimmunity. Therefore, the first step in our approach to T1D prevention was to determine the single doses of aldesleukin that increase Treg numbers within the physiological range, aiming to mimic the protection against T1D afforded by the risk-reducing alleles of the IL-2 pathway.

**What Did the Researchers Do and Find?:**

- We successfully employed a state-of-the-art dose-finding, open label, adaptive clinical trial design in 40 participants with T1D to determine the doses of aldesleukin needed to raise Treg frequencies by 10% and 20%.
- Increased Treg frequencies induced by aldesleukin were preceded by a peak of aldesleukin in the blood at 90 minutes that caused a transient dose-dependent decrease of Tregs and other cell subsets in the circulation.
- Following treatment with a dose of aldesleukin, Tregs had a decreased sensitivity to IL-2 that returned to baseline on day 3 after treatment.

**What Do These Findings Mean?:**

- The DILT1D trial showed that it is possible to conduct an adaptive dose-finding trial to estimate two drug doses based on an immune cell biomarker as a primary outcome.
- Partial desensitisation of Tregs might help explain why some participants in previous trials with daily dosing regimens did not respond to aldesleukin, suggesting that in future trials the drug should not be administered on a daily basis.

## Introduction

Type 1 diabetes (T1D) is the second most common chronic disease of children, and yet insulin replacement is the only therapy currently approved to treat the disease. Insulin replacement corrects insulin deficiency and hyperglycaemia but does not treat the underlying autoimmune T lymphocyte–mediated destruction of the insulin-producing β cells of the pancreatic islets [1]. The result is a lifelong requirement for intensive insulin treatment that increases the risk of hypoglycaemia and stops the majority of patients from achieving adequate metabolic control to prevent the long-term complications of retinopathy, neuropathy, and/or nephropathy [2,3]. Given these clinical outcomes, there have been intensive efforts over the last four decades to develop immunotherapies that suppress β cell autoimmunity in order to preserve endogenous insulin production, using standard trial methodologies [4–8]. While these approaches have achieved some success, an alternative strategy to identify potential immunotherapies is to use pathophysiological insights from human genetic associations to identify modifiable pathways [9,10].

The interest in modulation of the IL-2 pathway as a potential T1D therapy was initiated by mapping of a major susceptibility locus in the non-obese diabetic (NOD) mouse model to the IL-2 gene [11]. The mechanism for T1D susceptibility in NOD mice was identified as a reduction of regulatory T cell (Treg) function through reduced IL-2 production by the NOD susceptibility allele [12]. In humans, allelic variation of the IL-2 receptor gene, *IL2RA*, encoding the α subunit (CD25) was identified as a susceptibility determinant for T1D [13]. Genome-wide association studies identified a number of other T1D susceptibility genes in the IL-2 pathway, encoding critical proteins mediating immune activation and regulation (IL-2, IL-21, BACH2, PTPN2, IL-10) [14,15]. Analysis of the effect of the common susceptibility allele of the major causal variant in the *IL2RA* region showed that it decreased expression of CD25 on the surface of CD4^+^ effector T cells (Teffs) and Tregs [16,17]. Furthermore, the *IL2*-*IL21* T1D susceptibility region has been found to be associated with decreased numbers of IL-10 producing islet-specific CD4^+^ Teffs [18]. Thus, deficiencies in the IL-2 pathway and in genes expressed in CD4^+^ T cells, including Tregs, could contribute to disruption of the homeostatic equilibrium between IL-2-dependent Tregs and Teffs, which produce IL-2 to sustain the Tregs and enable them to regulate Teff activity during infections and maintain self-tolerance in health [19–23].

High-dose aldesleukin (Proleukin; recombinant human IL-2) is currently licensed for the treatment of adults with metastatic renal cell cancer and metastatic melanoma, administered intravenously at 600,000 IU/kg every 8 h for 5 d followed by 9 d of rest and a further two treatment cycles, if clinically tolerated [24,25]. Aldesleukin at these doses induces the activation and proliferation of Teffs and natural killer (NK) cells to destroy tumour cells. However, it was found that high-dose aldesleukin also expands the Treg pool such that patients with melanoma with enhanced expansion of Tregs had a worse outcome compared to those with fewer Tregs [26]. These observations, combined with the results of several preclinical murine studies in which aldesleukin selectively increased Tregs and prevented or reversed autoimmune diabetes, have focused efforts on the development of low-dose aldesleukin protocols to treat T1D and other immune disorders [27,28].

Most recently, proof of concept of the clinical benefit of low-dose aldesleukin has been reported for chronic graft versus host disease [29,30], hepatitis-C-virus-induced vasculitis [31], systemic lupus erythematosus [32], and alopecia areata [33]. In T1D, a small safety study was conducted in which relatively high doses of aldesleukin (4.5 × 10^6^ IU, three times per week for 1 mo) were combined with rapamycin, resulting in a transient loss of endogenous insulin production (as measured by C-peptide) in participants that was reversed on withdrawal of the therapy [34]. This has led to discontinuation of this combination [35]. In contrast, using lower doses of aldesleukin alone (range 0.33 × 10^6^ to 3.00 × 10^6^ IU) administered daily for 5 d induced an increase in Tregs without impairment of pancreatic β cell function [36]. The treatment regimen developed from this dose-finding study has now been carried forward into the DIABIL-2 clinical trial to assess the efficacy and safety of ultra-low doses of recombinant human IL-2 (rhIL-2) in children and adults with T1D [37].

These studies and trials share an initial induction phase of daily or every other day rhIL-2 administration or long-term daily dosing modelled on cancer therapy and a standard approach to drug development. In contrast, our goal is to establish a regular dosing regimen that could be given to children and adults shortly after diagnosis of T1D over a long period of time that maintains residual β cell function by protecting β cells from autoimmune Teffs through improving Treg function within physiological ranges. The underlying rationale is that we aim to mimic the protection from disease afforded by the risk-reducing alleles of the IL-2 pathway genes by establishing in experimental medicine studies a long-term dosing regimen that induces and maintains a stable, steady-state increase in Tregs within the normal physiological range, but that does not compromise immune defence against pathogens, responses to vaccines, or cancer immunosurveillance. This approach might avoid the potential complications and risks associated with prolonged administration of immunosuppressive treatments that preclude their use in the T1D prevention setting [38,39]. More intensive aldesleukin treatment regimens may be required for autoinflammatory disorders in which the tissue damage caused by the ongoing immune response causes pathology that may be life-threatening without immunosuppression, such as in systemic lupus erythematosus and chronic graft versus host disease. In contrast, the immune systems of patients diagnosed with T1D are relatively immunocompetent, with only small differences in lymphocyte phenotypes between cases and controls [18,40,41].

The first step in optimising the delivery of aldesleukin in T1D is to understand the kinetics of the biological effects of a single dose in adults with T1D. Here, in the Adaptive Study of IL-2 Dose on Regulatory T Cells in Type 1 Diabetes (DILT1D), the effects of single-dose aldesleukin were investigated by observing multiple biomarkers before treatment (baseline), at 90 min after drug administration, daily for 4 d, and then intermittently for 9 wk. The study used an adaptive design to initially estimate the Treg dose response to aldesleukin and then to identify the two doses of aldesleukin that increase Tregs minimally (10% relative to pretreatment) or maximally (20%) while remaining within the normal Treg frequency range.

## Methods

### Adaptive Study Design

The DILT1D trial was a 9-wk, single centre, non-randomised, open label, adaptive dose-finding trial that was conducted at the National Institute for Health Research Cambridge Biomedical Research Centre, Addenbrooke’s Hospital, Cambridge, United Kingdom. The study included 12 visits: after a screening visit, the aldesleukin dose was administered on day 0, and the participants were followed up, including blood sampling, 90 min after administration and subsequently on days 1, 2, 3, 4, 7, 9, 14, and 21, with a final follow-up visit on day 60. According to protocol amendments, visits between the seventh and 21st days were given 48 h of flexibility, and a safety visit on day 5 was removed. During protocol development, statistical simulations suggested that a sample size of 40 would give an informative estimate of the dose and target responses. The trial consisted of two phases: a learning phase (with ten individuals) and an adaptive phase (with 30 individuals). In the learning phase, participants received 0.04 × 10^6^, 0.16 × 10^6^, 0.60 × 10^6^, 1.00 × 10^6^, and 1.50 × 10^6^ IU/m^2^ of aldesleukin in ascending order, with each dose administered to two participants before escalation. Once the tenth participant had completed 7 d of follow-up, the clinical Treg and safety data were extracted from the trial database, and a preplanned interim analysis was carried out by the clinical trial statisticians by fitting a candidate set of parametric models to estimate the dose-response curve. A statistical analysis report was generated and delivered to the Dose Determining Committee (DDC)—consisting of a physician (S. Neupane, M. Evans, or F. Waldron-Lynch), a statistician (M. Evangelou, S. Bond, or A. P. Mander), and a scientist (J. A. Todd or L. S. Wicker)—within seven working days in order to select the doses to achieve the targeted Treg increases from baseline of 10% (minimal Treg increase) and 20% (maximal). In the adaptive phase, as soon as a participant had completed the day 7 visit, the accumulated data of the primary endpoint were analysed by the statisticians of the study. The DDC then reviewed the interim analyses and accumulated safety data before allocating the next recommended dose to the next participant of the adaptive phase. For each model, a set of doses to assign to the next patient cohort was identified that maximised the predicted reduction in the area of the 95% confidence region around the two estimated target doses (i.e., minimising the determinant of the covariance matrix) at the following interim analysis. The DDC resolved the infrequent cases where the models recommended different sets of doses to assign. The DILT1D protocol and novel governance structures that were developed to make regular dose decisions by committee were published prior to completion of the study and final analysis of the endpoints [42].

### Primary Endpoint

The primary endpoint is based on the percentage of CD4^+^ Tregs (defined as CD3^+^CD4^+^CD25^high^CD127^low^) within the CD3^+^CD4^+^ T cell gate following treatment with aldesleukin as measured by fluorescence-activated cell sorting (FACS). The maximum value observed in each participant’s profile in the follow-up period was identified, and the percentage change from the baseline value defined the primary endpoint.

### Secondary Endpoints

The following secondary endpoints were measured following treatment with aldesleukin: change in full blood count; change in Treg number, phenotype, and proliferation measured by FACS; change in cytokines and soluble receptors; change in Treg cell epigenetic profile (methylation status); change in Teff number, proliferation, and phenotype measured by FACS; change in lymphocyte subset cell number, proliferation and phenotype subsets and NK and natural killer T cells measured by FACS; and change in metabolic control as measured by self-monitoring of blood glucose and laboratory measurement of blood glucose and glycated haemoglobin (HbA1c) and C-peptide.

### Exploratory Endpoints

The following exploratory endpoints were measured following treatment with aldesleukin: change in intracellular T and NK cell signalling, measured in vitro by FACS; in vitro dose response to IL-2, measured to assess changes in intracellular T cell signalling; and change in Treg function, measured by a T cell suppression assay.

### Safety Assessments

The safety and tolerability of the treatment was assessed in trial participants by clinical history, insulin use, physical examination, temperature, blood pressure, heart rate, 12-lead electrocardiograms, blood glucose, HbA1c, clinical laboratory tests, and adverse event (AE) recording.

### Study Participants, Consent Procedure, and Safety Assessments

Potential participants were eligible for the study if they had a T1D duration of less than 2 y, were positive for at least one autoantibody (anti-islet cell, anti-GAD, anti-IA2, anti-ZnT8), were aged 18 to 50 y, and were living in the European Union. The date of diagnosis of T1D was established by referring physicians, diabetes specialist nurses, review of register records, and self-reporting by potential participants. Potential participants were excluded from the study if they had a history or evidence on screening of severe organ dysfunction, unstable diabetes, pregnancy, malignancy (within 5 y), hepatitis B or C, human immunodeficiency virus, organ transplantation, and/or donation of more than 500 ml of blood in the 2 mo prior to treatment.

Potential participants interested in enrolling in DILT1D were provided with a patient information sheet and an informed consent form to review. Individuals were given a minimum of 24 h to consider the information provided and then were contacted to determine if they remained interested in participating in the study or if they had any further queries. Interested potential participants were then invited to attend a visit where the Chief Investigator or delegate discussed the study with the participant, who then provided written informed consent before undergoing screening and any other trial-related procedures.

### Ethical Approval, Sponsorship, and Trial Registration

The trial was sponsored by the University of Cambridge and Cambridge University Hospitals NHS Foundation Trust. Ethical approval for the study was granted by the Health Research Authority, National Research Ethics Service, England (approval number: 13/EE/0020) on 18 February 2013. The study was registered in the ISRCTN Registry (ISRCTN27852285) on 26 March 2013 and at ClinicalTrials.gov (NCT01827735) on 4 April 2013.

### Clinical Immunophenotyping

All clinical flow cytometry (FACS) was performed following good clinical practice at the Department of Clinical Immunology, Addenbrooke’s Hospital, Cambridge, UK, within 4 h of phlebotomy. Operators were blinded to the aldesleukin dose allocated. A 2.6-ml sample of peripheral whole blood was collected into EDTA tubes, and 50 μl was stained with specific fluorochrome-conjugated antibodies at room temperature for 15 min to identify Tregs as CD3^+^CD4^+^CD25^high^CD127^low^ T cells. The clones used were anti-CD3 (clone SK7, phycoerythrin [PE]-Cy7-labelled; BD Biosciences), anti-CD4 (clone RPA-T4, FITC-labelled; BD Biosciences), anti-CD127 (clone HIL-7R-M21, PE-labelled; BD Biosciences), anti-CD25 (clone M-A251 and 2A3, allophycocyanin [APC]-labelled; BD Biosciences), anti-CD45RA (clone HI100, APC-Cy7-labelled; BioLegend), and anti-CD62L (clone DREG-56, PerCP/Cy5.5-labelled; BioLegend). Red cells were then lysed (BD FACS Lysing Solution); the cells were washed and resuspended in BD Cell Fix and then immediately analysed on a BD FACSCanto II flow cytometer utilising FACSDiva software (BD Biosciences). In parallel, a whole-blood BD Multitest 6-Color TBNK assay using BD Trucount Tubes according to the manufacturers’ instructions (BD Biosciences) was run to determine the relative and absolute concentration of lymphocyte subpopulations, including T, B, and NK cells.

### Mechanistic Immunophenotyping

For each participant, 30 ml of peripheral whole blood was collected into lithium heparin tubes and processed according to the workflow (S1 Fig). To determine the frequencies and phenotypes of NK and T cell subsets in whole blood within 4 h of phlebotomy, multicolour FACS was performed using the antibodies shown in S1 and S2 Tables. A full standard operating procedure is provided in S1 Materials. For surface staining, 150 μl of whole blood was incubated with fluorochrome-conjugated antibodies at room temperature for 45 min. Red cells were then lysed (BD FACS Lysing Solution), washed, and then analysed on a BD Fortessa flow cytometer. For intracellular staining, cells were treated with Intracellular Fixation & Permeabilization Buffer Set (eBioscience) after labelling with surface antibodies, washed twice with Permeabilization Buffer, and then incubated with an intracellular antibody panel (shown in grey in S1 Table) at 4°C for 45 min. Samples were analysed on a BD Fortessa flow cytometer using FACSDiva software (BD Biosciences) and FlowJo (Tree Star). Where possible, subset frequencies such as percent Tregs were expressed as an average obtained from the analysis of tubes 1–6 (S1 Table). To measure the effects in vitro of aldesleukin on CD25 and CD122 expression on lymphocyte subsets, heparinized venous blood (25 ml) from healthy volunteers was divided into ten 2-ml aliquots. Aliquots of whole blood were cultured with either 50 units/ml aldesleukin or medium alone (equivalent volume PBS + 2% BSA) at time points 0, 15, 30, 90, and 180 min. Samples were incubated at 37°C while being rotated at 35 degrees above horizontal. All samples were processed at 180 min for surface staining as per the described standard operating procedure, except that following addition of the antibody panel, samples were quenched on ice for 2 min and incubated at 4°C to prevent ongoing stimulation. The number of participants contributing to each secondary outcome analysis can vary due to missing data. The missing data, with a description of the quality control process, are summarised in S2 Materials.

### Cell Sorting

For in vitro functional assays, within 4 h of phlebotomy 3-ml whole blood samples were stained with fluorochrome-conjugated antibodies (S3 Table) for 1.5 h at room temperature, followed by the addition of 30 ml of RBC Lysis Buffer (eBioscience) for 10 min. Cells were washed and then resuspended in 500 μl of X-VIVO 15 + 1% AB serum for cell sorting with a BD FACS Aria II flow cytometer and FACSDiva software (BD Biosciences). CD4^+^ Tregs and Teffs were sorted by the expression pattern of CD25 and CD127: Tregs were defined as CD25^high^CD127^low^, and Teffs (non-Tregs) as CD25^low-medium^CD127^low-high^. Naïve and memory Teffs and Tregs were defined using CD45RA. Central and effector memory Teffs were defined by the presence or absence of CD62L, respectively.

### Flow Cytometric Analysis for pSTAT5

pSTAT5 staining was carried out as previously described [17]. Whole blood was either directly lysed and fixed ex vivo (Lyse/Fix Buffer, BD Biosciences) or stimulated for 30 min at 37°C with the indicated concentrations of aldesleukin diluted in X-VIVO 15 + 1% human AB serum (Lonza), then lysed and fixed. In some instances, 300 μl of blood was incubated for 2 h at 37°C alone, with anti-human-IL-2 antibody (20 μg/ml) (clone MQ1-17H12, purified NA/LE, BD Biosciences) or isotype control antibody (20 μg/ml) (clone RTK2758, purified NA/LE, BD Biosciences) prior to addition of lyse/fix buffer. After washing, cells were permeabilized with methanol (>99.9%, Sigma-Aldrich), washed, and stained with the fluorescence-conjugated antibodies detailed in S4 Table. Cells were acquired using a BD Fortessa flow cytometer (BD Biosciences) using FACSDiva software. To generate normalised results per lymphocyte cell subset for in vitro aldesleukin dose titration studies, the pSTAT5 mean fluorescence intensity (MFI) of cells not incubated with aldesleukin was subtracted from the pSTAT5 MFI at each aldesleukin dose and then divided by the pSTAT5 MFI observed in the maximal response of that cell subset.

pSTAT5 staining of aldesleukin-stimulated cryopreserved peripheral blood mononuclear cells (PBMCs) was carried out as previously described [43] in a batch manner, where each batch included 14 samples from participants and two biological controls that were kept constant for all experiments to enable normalisation of data influenced by day-to-day staining variation. Assays were conducted using selected participants across the dose groups at relevant time points in regard to Treg responses. PBMCs were stained with anti-CD4-APC-eFluor 780 (clone SK3, eBioscience), anti-CD25-PE (clones ZA3 and M-A251, BD Biosciences), anti-CD45RA-PE-Cy7 (clone HI100, BioLegend), anti-FOXP3-Alexa Fluor 488 (clone 236A/E7, BD Biosciences) and pSTAT5 (pY694)-Alexa Fluor 647 (clone 47, BD Biosciences) for 1 h at 4–8°C. Data acquisition was performed on a BD FACSCanto II (BD Biosciences) and analysed using FlowJo (Tree Star).

### In Vitro Suppression Assays

Suppression assays were performed in selected participants with Treg responses in V-bottom 96-well plates using cryopreserved PBMCs by coculturing 500 sorted CD4^+^CD25^low-medium^CD127^+^CD45RA^−^ memory Teffs (mTeffs) in the presence or absence of CD4^+^CD25^high^CD127^low^ Tregs at various ratios (Treg:Teff 0:1 to 1:8) with 1 × 10^3^ CD19^+^CD4^−^ B cells. Samples were stimulated with PHA (4 μg/ml; Alere) and incubated at 37°C, 5% CO_2_, for 6 d. Proliferation was assessed by the addition of 0.5 μCi/well [^3^H] thymidine (PerkinElmer) for the final 20 h of coculture. All conditions were run in 12 replicates, and proliferation readings (counts per minute [CPM]) averaged. Any samples with averaged proliferation less than 1,000 CPM from the mTeff wells alone were excluded. The percentage suppression in each culture was calculated using the following formula: percent suppression = 100 − [(CPM in the presence of Tregs ÷ CPM in the absence of Tregs) × 100].

### Methylation Status

The methylation status of the *FOXP3* Treg-specific demethylated region (TSDR) was analysed using a next-generation sequencing method that assesses the TSDR at single-base resolution [44].

### Cytokine Measurements

Plasma C-reactive protein (CRP) was measured using a custom V-PLEX Human CRP Kit according to manufacturer’s instructions (Meso Scale Diagnostics [MSD]) and read on the MSD Sector Imager 6000. Plasma IL-2 was measured using the MSD S-PLEX Human IL-2 Assay [45] (limit of quantitation 2 fg/ml) at MSD. In order to convert the plasma IL-2 (fg/ml) levels provided by MSD to plasma aldesleukin (IU/ml) levels, readings from the MSD S-PLEX Human IL-2 Assay at 90 min after aldesleukin administration for all DILT1D participants were compared to results from an in-house human IL-2 DELFIA-based immunoassay [46] that has a limit of quantitation of 10 pg/ml using aldesleukin (specific activity 16.4 IU/ng) as the standard curve. A linear relationship was observed between the two assays; a conversion of 3.4-fold was required to convert MSD S-PLEX Human IL-2 Assay readings to those obtained using an aldesleukin standard curve. This conversion factor was applied to all other samples tested at MSD, all of which had been below the limit of detection or quantification in the in-house assay. Plasma-soluble CD25 was measured in duplicate using an in-house sandwich immunoassay as described in [47].

### Statistical Analysis

#### Predefined populations of the study

Three populations were defined in this study: analysis, evaluable, and safety. The analysis population includes all participants who received a single dose of aldesleukin with a follow-up period of 7 d and for whom the primary endpoint was observed. The evaluable population includes all participants who received a single dose of aldesleukin, for whom the primary endpoint was observed, and who were not withdrawn from the study on clinical grounds. The safety population includes all participants who received a single dose of aldesleukin and were followed up for any period of time during the trial.

#### Primary endpoint analysis—interim analyses

The primary endpoint analysis was carried out at all conducted interim analyses of the trial as well as at the end of the trial using all accumulated data of the evaluable population. The aim of the primary analysis was to find the best model that describes the dose-response curve, and to identify the doses that achieve the minimum Treg increase (10%) and the maximum Treg increase (20%) targets selected by the DDC of the trial. The relationship of the primary endpoint with dose was explored by fitting a number of candidate models. The candidate models include the linear, the quadratic, the cubic, the logistic, and the Emax (with three and four parameters). The Akaike information criterion (AIC) and the deviance of each model were computed as measures of adequacy of fit. The proposed target doses of each model, with their standard errors and with a 95% confidence interval, were also computed. The primary endpoint analysis at the end of the trial was conducted on both the evaluable and the analysis populations (S1 Analysis).

#### Secondary endpoint analyses

For each time point and for each response/phenotype, two measures were defined: the absolute change and the percentage change. The absolute change is defined as the difference between the time point measurement and the baseline (pretreatment) measurement. The percentage change is defined as the ratio of the aforementioned absolute change to the baseline measurement. Based both on the absolute and on the percentage change, a number of derived variables were defined, including the 90-min measurement (where available), the day 1 measurement, and the maximum observed value from day 1 (or from 90 min where available) to day 7 times 100. One of the key hypotheses of interest is whether the absolute (or percentage) change at a particular time point of interest is zero. This hypothesis was tested using a *t*-test statistic. Equivalently, paired *t*-tests were used to compare two responses at a time point of interest, as reported in the results. The effect of dose on the derived variables computed using the percentage change was explored by fitting both a linear model and a cubic model. The model with the smallest AIC was chosen as the most representative model for fitting the data. A *p*-value of association of dose with the response was computed by comparing through an analysis of variance (ANOVA) the two aforementioned models with the model represented only by the intercept term. For cases where the linear model was the best fit, we report both the dose coefficient and the dose *p*-value; for cases where the cubic model was the best fit, we report only the dose *p*-value. The effect of dose on the derived variables computed using the absolute change was explored by comparing a series of models that included the effect of the baseline measurement of each tested response. The model with the smallest AIC was chosen as the best model. For the cases where the interaction and additive models were the best ones, they were compared with the baseline model for finding the effect of dose. A dose *p*-value was calculated through ANOVA. Similarly, for the models where the dose model was the best, a dose *p*-value was computed by comparing the dose model with the null model through ANOVA. For Treg CD25 MFI, pSTAT5 (normalised memory Treg) MFI, and FOXP3 MFI (CD25^+^FOXP3^+^ out of total CD4^+^ T cells) responses, we were interested in understanding how their measurements changed per day. Using the percentage change at all time points between 90 min and day 60 (or day 3 for pSTAT5), the relationship with dose was modeled either through a linear model or through a cubic model, both with no intercept term included. For each response, the model with the smallest AIC was chosen across the tested days. The best model was used for predicting the response and a 95% confidence interval around it for each time point for the two chosen doses derived from the primary endpoint analysis.

#### Average response plots and summary statistics

The range of doses administered was divided into five dose groups. These five groups are 0.040–0.045 × 10^6^, 0.160–0.380 × 10^6^, 0.385–0.610 × 10^6^, 0.620–0.990 × 10^6^, 1.00–1.50 × 10^6^ IU/m^2^, with 12, 8, 9, 7, and 4 participants in the analysis population, respectively. For each time point of either the absolute change or the percentage change, we averaged the time measurements of the participants within each dose group. This averaging was performed from the day 1 measurements (or from 90 min if applicable) to the day 7 measurements. For the participants whose visit on day 7 was truncated to an alternative day (±48 h, according to protocol amendment), their day 7 measurement was linearly interpolated using the closest measurements to day 7, which was subsequently used in the averaged response graphs. Throughout the manuscript the following statistics are reported for each phenotype: mean (standard error [SE], range) and the number of individuals (*n*).

## Results

### Participant Analysis Populations, Characteristics, and Study Design

**Fig 1:**
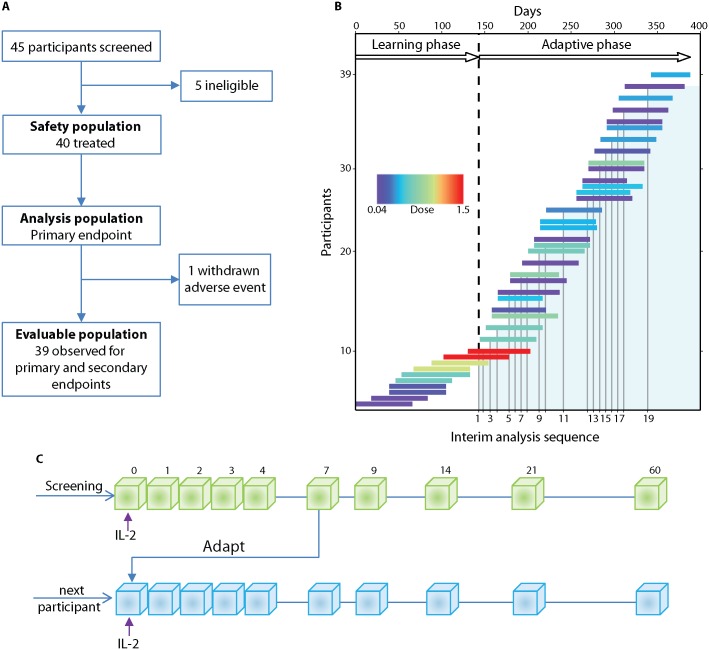
DILT1D study profile and adaptive design. (A) Flow chart showing the allocation of participants to the three predefined study populations: evaluable, safety, and analysis. (B) The study was conducted in two phases, a learning phase (140 d) and an adaptive phase (240 d). Individual participants are represented by a horizontal line, the length corresponding to the time from treatment until their final visit. In the learning phase, the first ten participants received a 0.04 × 10^6^, 0.16 × 10^6^, 0.60 × 10^6^, 1.00 × 10^6^, or 1.50 × 10^6^ IU/m^2^ dose of aldesleukin (colour represents dose allocated) in ascending order, with each dose being administered to two participants before escalation. In the adaptive phase, the DDC (S3 Materials) met on 19 occasions to review the interim safety data and allocate doses based on the analysis of the accumulated Treg data (shaded area) from all treated participants at that time. (C) Schematic of the study design illustrating that each participant who passed screening was administered a single dose of aldesleukin and followed for 60 d. During the adaptive phase, Treg data for every participant up to day 7 was included in an interim analysis and then further doses were allocated to the next participants. Treg, regulatory T cell; DDC, Dose Determining Committee.

DILT1D was an adaptive dose-finding study to increase Tregs as a proportion of total CD4^+^ T cells within the physiological range, with a sample size of 40. In total, 45 participants were enrolled in the study between 22 March 2013 and 15 May 2014, with the first participant recruited on 8 April 2013. Five participants were ineligible for the study due to the absence of a single T1D-associated antibody. Forty participants were treated with a single dose of aldesleukin (Proleukin, Novartis); one participant withdrew due to the development of a norovirus infection. Thirty-nine participants are included in the evaluable population, while 40 are included in the safety and analysis populations (Fig 1A). The demographic characteristics and autoantibody status at screening of the safety and analysis populations are shown in Table 1. Participants were recruited from the European Union to the study site at the University of Cambridge, United Kingdom, with 38 from the United Kingdom, 1 from Ireland, and 1 from France [48]. Most participants had been recently diagnosed with T1D (*n* = 36; duration 3–24 mo), with four newly diagnosed (< 3 mo). The DILT1D study team employed a response adaptive design, with a learning phase to find the shape of the dose-response curve and an adaptive phase to find the two aldesleukin doses that increase the target Treg frequency by 10% and 20% in the best statistical model (Fig 1B and 1C).

**Table 1:** Participant characteristics and autoantibody status.

Category	Characteristic	*N*	Mean (SE, Range)
**Demography (*n* = 40)**	Ethnicity white	40	—
	Male/female	30/10	—
	Age (years)	40	30.54 (1.32, 18.35–49.08)
	Body mass index (kg/m^2^)	40	23.88 (0.59, 16.70–32.00)
	Body surface area (m^2^)*	40	1.91 (0.03,1.55–2.23)
	Duration of disease (months)	40	10.74 (0.96, 0.86–23.70)
**Autoantibody status (*n* = 40)**	**Autoantibody type**		
	Anti-islet	14	—
	Anti-GAD	39	—
	Anti-IA2	19	—
	Anti-ZnT8	26	—
	**Autoantibody number**		
	One positive antibody	11	—
	Two positive antibodies	12	—
	Three positive antibodies	7	—
	Four positive antibodies	10	—

*DuBois formula.

SE, standard error.

There were no differences in haematological parameters between baseline and the final visit of participants (complete blood count, S5 Table; FACS analysis, S6 Table). Some clinical evidence of an improvement in metabolic control was recorded, with a decrease in participants’ mean glucose and HbA1c, accompanied by a reduction in the dose of basal insulin, though this may be a result of participants’ receiving a higher degree of specialist care by the trial team. There was no evidence of a decrease in random non-fasting C-peptide or the development of thyroid dysfunction (S7 Table).

### Primary Endpoint

**Fig 2:**
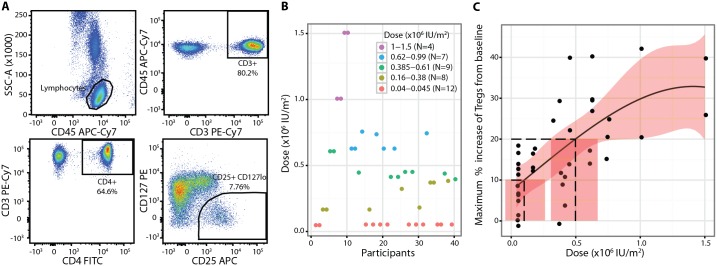
Regulatory T cell primary endpoint. (A) Percentage of Tregs was defined as the percentage of CD3^+^CD4^+^CD25^high^CD127^low^ cells within the CD3^+^CD4^+^ gate measured. (B) Individual participant dose allocations and dose groups showing convergence of the study to doses that achieve the two defined Treg targets. (C) A cubic model described the Treg dose response to aldesleukin best, with dashed lines showing the 10% and 20% Treg targets and doses. The shaded areas represent 95% CIs. Baseline, or pretreatment, Treg (percent of CD4^+^ T cells): 6.60% (SE = 0.25%, range 3.50%–10.70%, *n* = 39). SSC-A, side-scattered light-A; Treg, regulatory T cell.

The primary endpoint was the maximum percentage increase from the baseline of Tregs in each participant measured over the 7 d following treatment (Fig 2A) with adaptive dosing (Fig 2B). Tregs showed a dose response to a single administration of aldesleukin, with higher doses leading to larger increases (Fig 2C). This relationship was analysed in several candidate models, with the cubic model being found to be the best one to describe the dose-response curve as it had the smallest deviance and visually fitted the data the best (S2 Analysis). Analysis of the evaluable population for the primary endpoint found that the optimal doses of aldesleukin to induce 10% and 20% increases in Tregs were 0.101 × 10^6^ IU/m^2^ (SE = 0.078, 95% CI = −0.052, 0.254) and 0.497 × 10^6^ IU/m^2^ (SE = 0.092, 95% CI = 0.316, 0.678), respectively (Fig 2C). The lower confidence limit for the dose to increase Tregs by 10% is negative as a result of the estimated target dose being close to zero.

### Safety and Tolerability

Single doses of aldesleukin were well tolerated at all doses, with no serious AEs reported (Table 2). Non-serious AEs were clinically graded as mild (easily tolerated), moderate (discomfort), or severe (unable to carry out usual activities) and on their relatedness to aldesleukin administration (causality). There were 45 unexpected non-serious AEs reported by 28 participants in the safety population. Sixteen had a single event, nine had two events, and three had three or more events, all resolving without residual effects. Three events (two episodes of nasal congestion on day 1 and one immediate injection site reaction) were assessed as related to aldesleukin and were classified as adverse reactions.

**Table 2:** Summary of non-serious adverse events and reactions (safety population).

Type of AE	Category	Details	*N*
**Unexpected AEs and reactions (*n* = 45)**	**Grade**		
	Mild		39
	Moderate	Common cold (2), asthma exacerbation (1), hand abrasion (1), headache (1)	5
	Severe	Norovirus infection (1)	1
	**Relatedness to aldesleukin**		
	Unrelated		31
	Unlikely		11
	Possibly*	Nasal congestion (2)	2
	Almost certainly*	Acute site reaction (1)	1
**Expected AEs–injection site reaction (*n* = 45)**	**Erythema**		38
	**Nodule**		29

Values in parentheses are the numbers within each category detail.

*Adverse reaction.

AE, adverse event.

A single participant had a severe AE that consisted of a transient self-limiting gastrointestinal event commencing 30 h after administration of a dose of aldesleukin (S2 Fig). Prior to administration of aldesleukin, clinical history, examination, and laboratory investigations were normal, apart from a low neutrophil count (2.3 × 10^9^/l [normal range: 4 × 10^9^/l to 11 × 10^9^/l]). The clinical presentation and course were consistent with a norovirus infection. To confirm the clinical diagnosis and identify the pathogen, we carried out serology testing for antibodies to norovirus and detected the induction of GII.4 IgG antibody titres at day 9 (S2 Fig), peaking at day 21 post-infection and declining thereafter, confirming that the participant had been infected with norovirus.

Most participants had an expected AE at the injection site consisting a non-itchy, local (1–5 cm), non-painful erythematous rash on day 1 followed by a subcutaneous nodule on day 2 that resolved on average by day 10 (SE = 2, range 1–58 d; S3 Fig). Aldesleukin had no effect on renal, bone, or liver biochemistries during the study (S8 Table).

### Secondary and Exploratory Endpoints

#### Transient decrease in lymphocytes in blood

**Fig 3:**
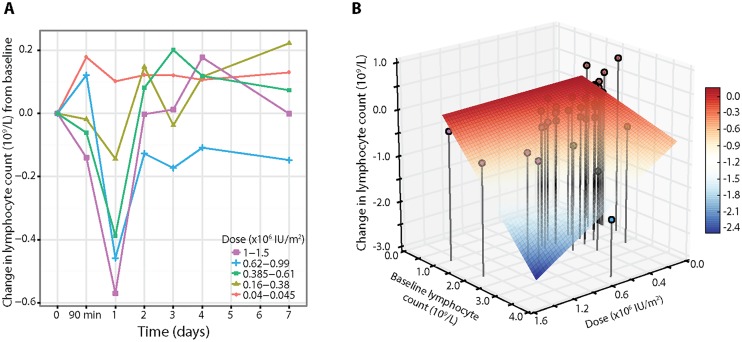
Lymphocyte responses to a dose of aldesleukin. (A) Average response curves of the absolute change in lymphocyte count across the five dose groups (average baseline lymphocyte count 1.78 × 10^9^/l, SE = 0.08, range 0.95–3.84, *n* = 39). (B) Three-dimensional plot of dose, baseline lymphocyte count, and change in lymphocyte count on day 1, with lines representing the vertical projections of points (coloured by dose) on the dose/baseline lymphocyte count axis. The surface grid represents the regression model for change in lymphocyte count on day 1 (colour scale), showing that the decrease in lymphocytes depends both on dose and pretreatment count.

Full blood count analysis stratified by dose showed a decrease in lymphocyte count in participants treated with doses greater than 0.045 × 10^6^ IU/m^2^ on day 1, with a recovery by day 2 (Fig 3A; S5 Table). To investigate if the pretreatment lymphocyte count affected the day 1 response, a set of candidate models that included the effects of both baseline lymphocyte count and dose was analysed. The change in lymphocyte count was found to be associated with dose through a model that depended both on baseline lymphocyte count and dose (*n* = 39, dose *p* = 0.006; Fig 3B), with the negative relationship to baseline lymphocyte count meaning that the higher the pretreatment lymphocyte count, the greater the reduction in count on day 1 (S3 Analysis). The greatest decrease in lymphocytes was observed in participants who received the highest doses (1.0 × 10^6^ and 1.5 × 10^6^ IU/m^2^; *n* = 4), with one participant developing a transient asymptomatic lymphopenia (count < 1 × 10^9^/l) that resolved by day 2. The white blood cell and neutrophil count decreased on average by approximately 10% on day 1, without a dose response, while the monocyte and basophil counts were unchanged by aldesleukin treatment (S5 Table).

#### Early transient increase in eosinophils related to aldesleukin dose and pretreatment count

**Fig 4:**
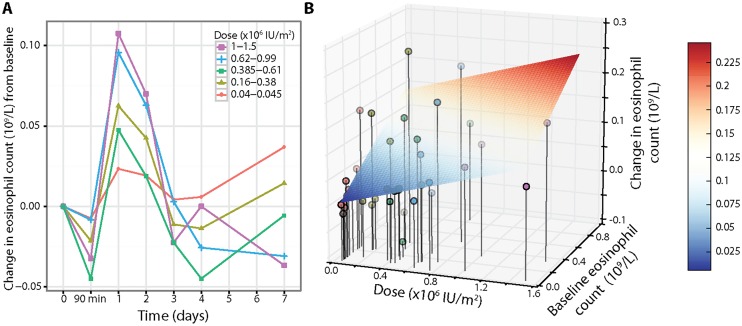
Eosinophil response depends on baseline counts and aldesleukin dose. (A) Eosinophil counts showed an initial transient decrease at 90 min in a hyperacute response to aldesleukin followed by a dose-dependent increase on day 1, with a return to baseline by day 3–4 (average baseline eosinophil count 0.15 × 10^9^/l, SE = 0.03, range 0.04–0.86, *n* = 39). (B) Three-dimensional plot of dose, baseline eosinophil count, and change in eosinophil count on day 1, with lines representing the vertical projections of points (coloured by dose) on the dose/baseline eosinophil count axis. The change in eosinophil count is affected by both dose and baseline eosinophil count using a linear dose-response model, with the grid showing the regression model (colour scale) for increase in eosinophils on day 1 (colour scale) (absolute change in eosinophil count on day 1 = −0.0058 + [0.0693 × dose] + [0.1748 × baseline]).

There was an asymptomatic decrease in eosinophils of approximately 15% at 90 min, followed by an increase on day 1 that resolved by day 3–4, with six participants developing a transient eosinophilia (count > 0.4 × 10^9^/l) (Fig 4A; S5 Table). Given the increase and development of eosinophilia, we analysed the eosinophil counts further to explore if the change was related to both dose and baseline eosinophil count. We found that the change in eosinophil count on day 1 was dependent on the additive effects of dose through a model that included both baseline eosinophil count and dose effects (*n* = 39, dose *p* = 0.026; Fig 4B). We found that the relationship was positive with a higher pretreatment eosinophil count, resulting in a greater increase in eosinophils on day 1.

#### Rapid circulatory changes in regulatory T cell numbers

**Fig 5:**
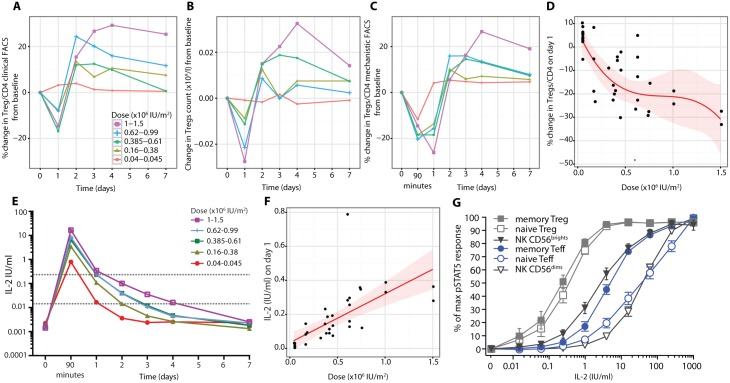
Hyperacute regulatory T cell response to aldesleukin. (A) Treg proportions as a percent of CD4^+^ T cells (average Treg level 6.6%, SE = 0.2%, range 3.5%–10.7%, *n* = 39) and (B) Treg counts following injection of aldesleukin as measured by the clinical grade FACS assay in conjunction with the BD Multitest TBNK assay are shown (average baseline Treg count 0.06 × 10^9^/l, SE = 0.01, range 0.02–0.14, *n* = 39). (C) Tregs as a percent of CD4^+^ T cells were measured in the mechanistic FACS assay (average Treg level 6.99%, SE = 0.27%, range 3.93%–10.74%, *n* = 37). (D) The decline of Tregs in blood on day 1 fits a cubic model (shaded area presents the 95% CI, *n* = 37). (E) Plasma IL-2 levels following aldesleukin dosing. The dotted grey lines mark the 0.015 and 0.24 IU/ml concentrations that are the threshold levels of aldesleukin at which Tregs, and Teffs and NK CD56^bright^ cells, respond, respectively. (F) Relationship between the dose of aldesleukin administered and the plasma concentration of IL-2 at day 1 in vivo. (G) Aldesleukin dose-response curves generated in whole blood from DILT1D participants for pSTAT5 responses within individual cell populations on day 60 post-treatment (mean with bars showing 95% CI, *n* = 39). (A–C) show averaged response plots across the five dose groups. (D) and (F) show the best fitted models with 95% CI. Teff, effector T cell; Treg, regulatory T cell.

Utilising clinical grade FACS assays, we observed a rapid decrease on day 1 after aldesleukin administration in the frequency of Tregs (Fig 5A; S6 Table) and in Treg numbers (Fig 5B). This decrease was followed by increases in Tregs over baseline, reaching a maximum by day 3 (SE = 0.31 d, range 1–7 d, *n* = 39), and then a gradual return to, or towards, baseline by day 7 for most participants. At the highest doses, there were transient decreases in the counts of CD8^+^ T cells, B lymphocytes (CD19^+^CD3^−^), and NK cells (CD3^−^CD56+CD16^+^CD19^−^) on day 1, recovering to baseline by day 4 (S4 Fig).

Mechanistic studies were performed in parallel with the clinical grade FACS measurements (Fig 5C), with strong agreement between the measurements of Treg frequency from the two independent sources (*n* = 377, *t*-test *p* = 2.2 × 10^−16^; S5 Fig). Henceforth, the data generated by these mechanistic assays are presented, unless otherwise stated.

Measurement of Tregs in whole blood samples after 90 min revealed that the reduction was rapid and sustained, with Treg percentage remaining below baseline on day 1 at all doses greater than the lowest dose range (Fig 5C). At 90 min the decline in Tregs was not dose dependent, but by day 1, the reduction in Tregs was dose dependent, with higher doses of aldesleukin leading to greater declines in Tregs (*n* = 37, dose *p* = 1.32 × 10^−5^; Fig 5D).

Using a recently developed IL-2 assay that is 5,000-fold more sensitive than conventional assays, we established that baseline levels of IL-2 in the DILT1D participants were 12.17–64.07 fg/ml (0.0007–0.0036 IU/ml) (Fig 5E), similar to those reported for healthy individuals [45]. At 90 min following subcutaneous dosing, which is near the time of peak blood concentration of aldesleukin using this route of administration [49], IL-2 plasma levels ranged from 0.35 to 27.46 IU/ml depending on the dose delivered and averaged 5.73 IU/ml (SE = 1.07, *n* = 37) (Fig 5E). There was a linear relationship between the dose of aldesleukin administered and the plasma concentration of IL-2 at day 1 in vivo (Fig 5F). All aldesleukin doses produced IL-2 blood levels at 90 min sufficient to increase pSTAT5 in a portion of Teffs and NK CD56^bright^ (CD56^high^TCR^α/β−^) cells in addition to a majority of the Tregs (Fig 5G); this finding was confirmed by assessing pSTAT5 levels in various cell types ex vivo at 90 min following aldesleukin dosing. Aldesleukin plasma levels remained above the 0.015 IU/ml concentration required to cause signalling in naïve Tregs (nTregs) and memory Tregs (mTregs) for up to 4 d, and above the 0.24 IU/ml concentration required for mTeff and NK CD56^bright^ cell activation for up to 1 d, depending on dose. Thus, although Tregs have an approximately 10-fold higher affinity for aldesleukin than do Teffs and NK CD56^bright^ cells, a completely Treg-specific aldesleukin plasma level was not achieved, even at the lowest doses administered.

#### Change in Treg phenotype

**Fig 6:**
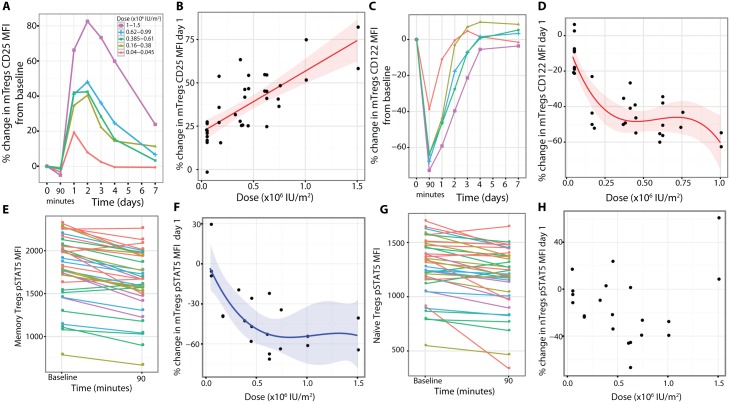
Phenotypes of the residual circulating regulatory T cells at day 1. (A and B) CD25 expression was increased on mTregs (average baseline CD25 MFI on mTreg = 7,412, SE = 181, range 5,119–9,393, *n* = 37). (C and D) Concurrently, there was a dose-dependent reduction in CD122 on mTregs in blood (baseline CD122 MFI on mTreg = 444.2, SE = 14.0, range 288.0–616.0, *n* = 33). (E) There was a reduction in pSTAT5 levels in mTregs incubated with a saturating concentration of aldesleukin (1,000 IU/ml) in vitro when assessing blood obtained 90 min after dosing of aldesleukin. (F) At day 1 post-dosing, there was a dose-dependent reduction in the percentage of mTregs that were pSTAT5^+^ following incubation with 0.4 IU/ml aldesleukin in vitro (percent of pretreatment time point mTregs that were pSTAT5^+^ following aldesleukin incubation: 56.25%, SE = 1.60%, range 43.23%–71.03%, *n* = 22). (G) There was a reduction in pSTAT5 levels in nTregs assessed 90 min post-dosing when the cells were incubated with a saturating dose of aldesleukin (1,000 IU/ml) in vitro. (H) At day 1 post-dosing, there was not a consistent change from baseline in the percentage of nTregs that were pSTAT5^+^ following incubation with 0.4 IU/ml aldesleukin in vitro (baseline percent of nTregs that were pSTAT5^+^ following incubation with 0.4 IU/ml aldesleukin: 58.01%, SE = 1.65%, range 40.83%–69.88%, *n* = 21). (A) and (C) show averaged response plots across the five dose groups. (B), (D), and (E) show the best fitted models with 95% CIs. MFI, mean fluorescence intensity; mTreg, memory regulatory T cell; nTreg, naïve regulatory T cell.

In order to determine if there was a change in Treg phenotype in blood after treatment, we analysed expression of CD25 and CD122 on Tregs before and after treatment. At baseline, mTregs have approximately on average a 55% higher expression of CD25 and a 90% higher expression of CD122 than nTregs (S6 Fig). Because of this heterogeneity between Treg subsets, mTregs and nTregs were assessed separately as well as together as total Tregs. At higher aldesleukin doses there were initial small decreases of CD25 expression on mTregs at 90 min (Fig 6A), followed by a large dose-dependent increase on day 1 in response to treatment (*n* = 37, dose coefficient = 35.24, dose *p* = 6.68 × 10^−8^; Fig 6B). The increase in CD25 in total Tregs was maximal on day 1.59 (SE = 0.13, range 1–4 d, *n* = 37) and was dose dependent (*n* = 37, dose coefficient = 35.40, *p* = 3.02 × 10^−8^; S7 Fig). A greater increase was observed in the mTreg compared to the nTreg subset (maximum observed percentage change of the CD25 MFI: mTreg = 43.1%, SE = 3.7%, range 1.5%–106.0%; nTreg = 28.4%, SE = 3.0%, range 1.8%–89.2%, *n* = 37, *t*-test *p* = 8.15 × 10^−9^). This mTreg increase was sustained over at least 4 d for doses greater than 0.045 × 10^6^ IU/m^2^ (Fig 6A). In contrast to changes observed for Treg CD25 expression, CD122 levels on the mTregs remaining in the blood were substantially lower at 90 min post-dosing. This was dose dependent at day 1 (*n* = 33, dose *p* = 1.17 × 10^−6^; Fig 6C and 6D). This lower CD122 expression was still prevalent at day 2, with CD122 levels on mTregs returning to baseline by day 3 at all doses except the two highest doses, 1.0 × 10^6^ and 1.5 × 10^6^ IU/m^2^, with similar changes in CD122 observed in nTregs (S6 Fig). A rapid disappearance of surface CD122 was also observed in vitro when Tregs were incubated with aldesleukin (S8 Fig).

We investigated if there were functional changes in the Treg subpopulations remaining in blood after aldesleukin treatment, focusing on phenotypes that could be altered by lower CD122 cell-surface levels. Whole blood samples at baseline and 90 min post-dosing were compared for their ability to respond to aldesleukin at a concentration that saturates the high-affinity IL-2 receptor (1,000 IU/ml). We found that the mTregs at 90 min responded to the high concentration of aldesleukin in vitro with lower pSTAT5 levels compared to mTregs at baseline (*n* = 36, *p* = 1.28 × 10^−9^; Fig 6E). In order to replicate and extend our findings, pSTAT5 was measured in Treg subsets from cryopreserved PBMCs isolated at baseline and day 1 that were incubated with 0.4 IU/ml aldesleukin to provide a submaximal stimulation. In the mTreg subset we observed that dosing with aldesleukin resulted in a transient dose-dependent decrease in the percentage of mTregs that became pSTAT5^+^ on day 1 post-dosing (*n* = 21, dose *p* = 9.60 × 10^−4^; Fig 6F). In contrast, although nTregs did show a reduction in pSTAT5 at 90 min after administration of aldesleukin (*n* = 37, *p* = 0.0002; Fig 6G), it was less profound than that observed for mTregs, and there was not a consistent reduction in signalling when PBMCs were stimulated with 0.4 IU/ml of aldesleukin (Fig 6H).

#### Sustained Treg phenotypes and functional responses

**Fig 7:**
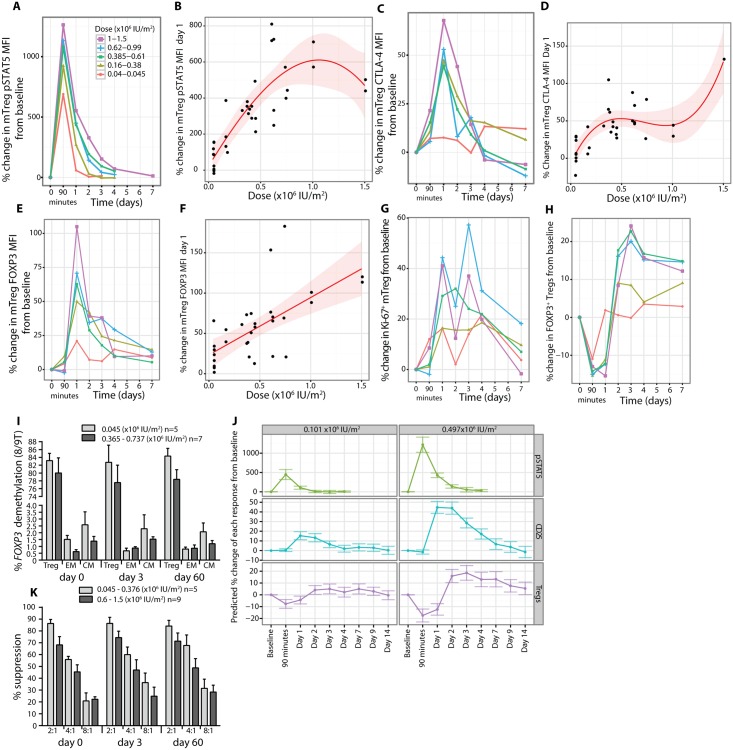
In vivo regulatory T cell phenotypes and functional responses to aldesleukin. (A and B) mTregs had their maximum pSTAT5 response to treatment at 90 min, and a detectable response was sustained for up to 4 d at the higher doses and was dose dependent on day 1 with a cubic dose response (average baseline pSTAT5 MFI = 7.36, SE = 0.33, range 4.64–12.53, *n* = 36). (C–F) Following activation, mTregs had a dose-dependent increase in CTLA-4 and FOXP3 expression, returning to baseline by day 3–4 post-dosing (mTreg CTLA-4 MFI = 1,539, SE = 65, range 877–2,411, *n* = 32; and mTreg FOXP3 MFI = 1,174, SE = 68, range 580–2,009, *n* = 34). (G) Concurrent with these changes on day 1, there was an increase in proliferation of mTregs in blood (baseline Ki-67^+^ mTreg = 15.27%, SE = 0.86%, range 7.10%–30.20%, *n* = 33). (H) Intracellular staining of Tregs from whole blood for FOXP3 showed an increase in FOXP3^+^ Tregs on day 3 (FOXP3^+^ Tregs/CD4^+^ T cells = 6.44%, SE = 0.25%, range 4.03%–10.30%, *n* = 37). (I) Analysis of FOXP3 gene demethylation on total Tregs and CD62L^low^ (effector memory) and CD62L^high^ (central memory) CD4^+^ memory T cells sorted from whole blood at pretreatment, post-treatment (day 3), and the last visit (day 60) showing stability of this Treg phenotype. (K) Tregs expanded in vivo at day 3 post-aldesleukin suppressed in vitro proliferation of autologous Teffs equivalently to Tregs at day in a suppression assay across the dose range (Treg:Teff ratio) tested. Error bars in (I) and (K) represent SEs. (J) Predictive cubic models based on the study data for CD25, pSTAT5, and Treg responses at the doses identified to increase Tregs by 10% and 20%. The error bars present the 95% confidence intervals around the predictions by these models. CM, central memory; EM, effector memory; MFI, mean fluorescent intensity; mTreg, memory regulatory T cell.

As measured by ex vivo pSTAT5 levels, mTregs were more responsive to in vivo aldesleukin treatment than nTregs at 90 min (*n* = 36, *t*-test comparison *p* = 1.46 × 10^−9^; Figs 7A and S9). The pSTAT5 response in mTregs at 90 min and at day 1 was related to dose, suggesting that the maximum response had been observed (*n* = 36, *p* = 0.001, and *n* = 36, *p* = 8.32 × 10^−9^, respectively) (Fig 7B). The day 1 ex vivo pSTAT5 signal could be neutralised in vitro by the addition of anti-IL-2 antibodies to the assay (S9 Fig), which confirms that it is the aldesleukin remaining in the blood that is responsible for the elevated levels of pSTAT5 ex vivo.

Similarly, a dose-dependent relationship was found for CTLA-4 MFI (*n* = 31, *p* = 4.92 × 10^−6^; Fig 7C and 7D), FOXP3 MFI (*n* = 33, *p* = 3.58 × 10^−5^, dose coefficient = 71.18; Fig 7E and 7F), and Ki-67 expression (mTregs) (*n* = 33, *p* = 0.03, dose coefficient = 39.14; Fig 7G) on day 1, with maximum increases on average by day 1.61 (SE = 0.27, range 0.1–7 d), 1.74 (SE = 0.29, range 0.1–8 d), and 2.16 (SE = 0.27, range 0.1–7 d), respectively. The findings on day 1 were consistent with an independent analysis of cryopreserved PBMCs from day 3 post-dosing, when the Treg count in the blood is greatest. The circulating mTregs and nTregs are more responsive to aldesleukin with higher CD25 (*n* = 15, *p* = 3.76 × 10^−5^, and *n* = 15, *p* = 2.83 × 10^−6^, respectively; S9 Fig) and FOXP3 (*n* = 15, *p* = 0.01, and *n* = 15, *p* = 0.005, respectively; S9 Fig) expression than at baseline. The overall effect of a dose of aldesleukin on Tregs by day 3 was to have increased the number of fully suppressive, demethylated FOXP3^+^ Tregs, which we presume have trafficked through tissues and been activated by self-antigens (Fig 7H, 7I and 7K).

The data generated in the study was used to develop a predictive model for each of these Treg responses and applied it to investigate the effects of the optimal doses of aldesleukin to induce 10% and 20% increases in Tregs, 0.101 × 10^6^ IU/m^2^ and 0.497 × 10^6^ IU/m^2^, respectively. At 0.101 × 10^6^ IU/m^2^, Treg pSTAT5 peaks at 90 min and then returns to baseline by day 2, while CD25 MFI and Treg frequencies are increased for 3 d. The 0.497 × 10^6^ IU/m^2^ dose had a more sustained predicted response, showing elevated Treg pSTAT5 for up to 3 d and total Treg frequencies not quite at baseline at 7 d, with an increase in CD25 expression for up to 7 d (Fig 7J).

#### Effector T cell responses

**Fig 8:**
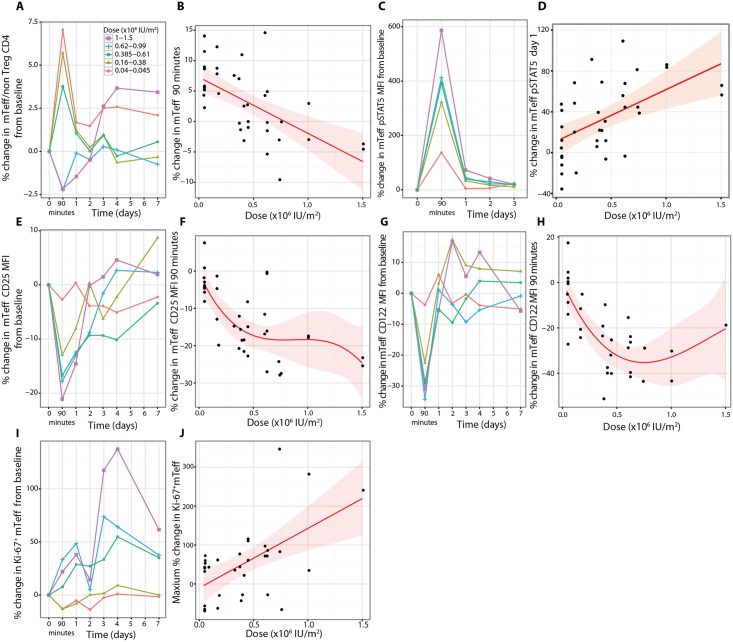
Effects of aldesleukin on effector T cell number, phenotypes, and proliferation. (A) mTeffs were responsive to aldesleukin, with their frequencies as a percentage of non-regulatory CD4^+^ T cells altered in circulation (B), resulting in opposing effects, with lower doses leading to higher mTeff frequencies and higher doses leading to reduced frequencies (average baseline mTeff percent of non-regulatory CD4^+^ T cells: 61.1%, SE = 1.9%, range 38.6%–87.4%, *n* = 37). (C and D) There was increased pSTAT5 in mTeffs (mTeff pSTAT5 MFI = 7, SE = 0.3, range 4–13, *n* = 36). Concurrently there was a dose-dependent decrease in CD25 (average baseline mTeff CD25 MFI = 1,005, SE = 31, range 676–1,436, *n* = 37) (E and F) and in CD122 (MFI = 137, SE = 7, range 59–248, *n* = 33) (G and H). (I and J) There was a dose-dependent increase in proliferation of mTeffs as measured by an increase in Ki-67^+^ mTeffs over the 7 d following treatment (baseline Ki-67^+^ Teffs = 2.92%, SE = 0.32%, range 0.75%–10.40%, *n* = 33). MFI, mean fluorescence intensity; mTeff, memory effector T cell; Treg, regulatory T cell.

There were rapid dose-dependent changes in the proportions of mTeffs following aldesleukin treatment, with increases at 90 min at doses less than 0.61 × 10^6^ IU/m^2^ and decreases below this dose, suggesting that there may be a dose threshold for this response (*n* = 37, *p* = 6.40 × 10^−5^, dose coefficient = −9.23; Fig 8A and 8B). At the two highest doses (1.0 × 10^6^ and 1.5 × 10^6^ IU/m^2^, *n* = 4), there was a sustained increase in mTeffs for days 3–7 (Figs 8A and S10). pSTAT5 levels in mTeffs increased at 90 min dose-dependently (*n* = 36, dose *p* = 2.71 × 10^−7^; Fig 8C). The increase in pSTAT5 remained detectable on day 1 at all doses greater than 0.045 × 10^6^ IU/m^2^, with the two highest doses giving a larger and more prolonged response, an observation verified by the linear dose relationship on day 1 (*n* = 36, *p* = 6.24 × 10^−4^, dose coefficient = 50.58; Fig 8D). There was a transient small increase in CD69 expression at all doses 90 min post-treatment for both mTregs and mTeffs (S10 Fig). There was a small transient reduction in IL-2 sensitivity at 90 min in mTeffs compared to Tregs, at all doses, that returned to baseline by day 1 (*n* = 37, *t-*test *p* = 8.18 × 10^−5^; S10 Fig). There was a rapid dose-dependent reduction in expression of CD25 on mTeffs in blood that remained below baseline until day 3, except in participants who received the two highest doses, who had a sustained increase above baseline from day 3 onwards (*n* = 37, *p* = 5.15 × 10^−6^; Fig 8E and 8F). For CD122 on mTeffs in blood, there was initially a dose-dependent decrease (*n* = 33, *p* = 1.46 × 10^−5^; Fig 8G and 8H) followed by an increase on days 1–4 in participants who received the larger doses. Similar patterns of CD25 and CD122 expression changes were observed in vitro when mTeffs were incubated with aldesleukin (S8 Fig). To determine if there was evidence of proliferation of mTeffs, we measured Ki-67 and found that there was a linear dose relationship (*n* = 33, *p* = 1.93 × 10^−3^, dose coefficient = 145.71; Fig 8I and 8J).

#### Natural killer, natural killer CD56^bright^, and natural killer T cell responses

**Fig 9:**
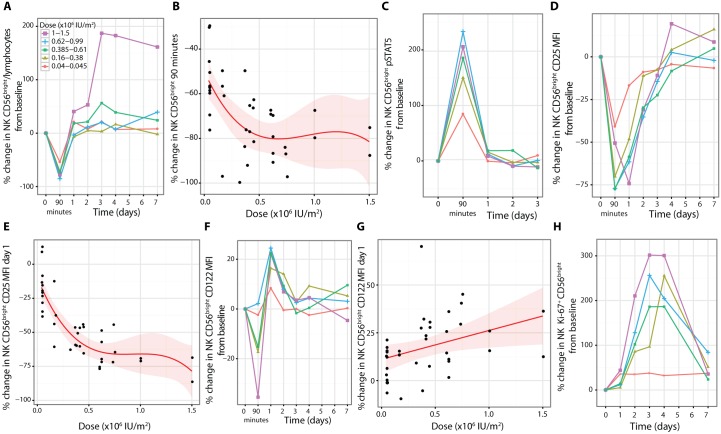
Effects of aldesleukin on NK CD56^bright^ cell number, phenotypes, and proliferation. (A and B) NK CD56^bright^ cells showed a rapid dose-dependent decline, with the majority of cells not in circulation at 90 min (NK CD56^bright^ cells percent of lymphocytes: 0.41%, SE = 0.03%, range 0.09%–0.96%, *n* = 38). (C) Concurrent with this decline is a dose-dependent increase in NK CD56^bright^ cell pSTAT5 levels (baseline pSTAT5 MFI = 16.55, SE = 0.70, range 9.51–27.87, *n* = 37). (D and E) There was a sustained dose-dependent reduction in expression of CD25 (MFI = 642, SE = 32, range 255–1,148, *n* = 38) on NK CD56^bright^ cells and (F) a transient reduction in CD122 at 90 min (G) followed by a linear dose-dependent increase on day 1 (baseline CD122 MFI = 6,605, SE = 213, range 3,786–9,554, *n* = 38). (H) The outcome of treatment was increased proliferation of NK CD56^bright^ cells (baseline percentage of Ki-67^+^ NK CD56^bright^ cells = 9.9%, SE = 0.9%, range 3.35%–25.9%, *n* = 30). MFI, mean fluorescence intensity; NK, natural killer.

Treatment at all aldesleukin doses caused a decline of total NK cells in circulation at 90 min, with the largest decrease in the NK CD56^bright^ cells compared to the NK CD56^dim^ (CD56^low^TCR^α/β−^) cells (*n* = 38, *t-*test comparison *p* = 1.00 × 10^−10^) (Figs 9A and S11). The decline in NK CD56^bright^ cells at 90 min was dose dependent (*n* = 38, dose *p* = 0.0005; Fig 9B). NK CD56^bright^ cells were the NK subset most responsive to treatment, with a greater increase in NK CD56^bright^ cells compared to NK CD56^dim^ cells (*n* = 32, *t-*test comparison *p* = 6.83 × 10^−11^) (Figs 9A and S11) and a larger pSTAT5 response (Figs 9C and S11).

At baseline, NK CD56^bright^ cells had approximately 250% more expression of CD25 and 100% more expression of CD122 than the NK CD56^dim^ cells (S11 Fig). Following treatment, there was a rapid dose-dependent reduction in expression of CD25 on the NK CD56^bright^ cells remaining in the blood (Fig 9D and 9E; *n* = 37, dose *p* = 2.09 × 10^−6^), and this change was greater than in the NK CD56^dim^cells at 90 min (S11 Fig; *n* = 37, *t-*test *p* = 5.37 × 10^−9^). In contrast to the Treg response, a substantial decrease in CD25 expression on NK CD56^bright^ cells was observed that remained below baseline for up to 4 d after treatment (Fig 9D). CD122 on NK CD56^bright^ cells transiently decreased by a much more modest amount at 90 min compared to CD25, followed by a dose-dependent linear increase (*n* = 38, *p* = 0.02, dose coefficient = 15.10) (in contrast to CD122 on Tregs; Fig 6C) that returned to baseline by day 3 (Fig 9F and 9G). Similar patterns of CD25 and CD122 expression changes were observed in vitro when NK CD56^bright^ cells were incubated with aldesleukin (S8 Fig). To assess the functional effects of treatment on NK homeostasis, the proliferation marker Ki-67 was measured and it was observed that the greatest increase was in the NK CD56^bright^ cells at all doses administered (*n* = 30, *t-*test comparison *p* = 0.0012; Figs 9H and S11). However, in the 1.0 × 10^6^ to 1.5 × 10^6^ IU/m^2^ dose range there was a reduction in the specificity since proliferation of both NK subsets was similar (~300% increase; Figs 9H and S11).

There was a dose-dependent change in the frequency of natural killer T cells (CD56^+^TCR^α/β+^) cells in circulation following aldesleukin treatment, with a decrease at 90 min at doses less than 0.61 × 10^6^ IU/m^2^ and little change (~10%) at higher doses. Only at the two highest doses, 1.0 × 10^6^ and 1.5 × 10^6^ IU/m^2^, was there evidence of a sustained increase (~100%), with a decline towards baseline by day 7 (S11 Fig).

#### Changes in inflammatory markers soluble CD25 and C-reactive protein

There was a decrease in serum soluble CD25 at 90 min followed by an increase in levels of this immune activation marker at day 1 (S12 Fig). A linear relationship was found between dose and the percentage change of soluble CD25 on day 1 (*n* = 39, dose coefficient = 13.94, *p* = 0.005; S12 Fig). The highest doses (1.0 and 1.5 × 10^6^ IU/m^2^) had the longest duration of response, taking 7 d to return to baseline. We measured CRP to determine if there was evidence of an acute phase response following treatment and observed that there was a dose-dependent increase in CRP on day 1, where doses greater than 0.62 × 10^6^ IU/m^2^ are estimated to increase CRP greater than 100% (S12 Fig).

#### Increases in chemokine expression on Tregs

**Fig 10:**
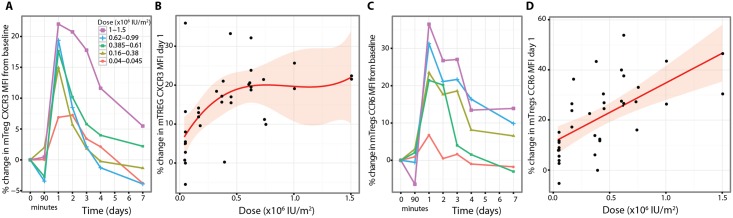
Aldesleukin upregulates CXCR3 and CCR6 on regulatory T cells. (A and B) Dose-dependent sustained increase in expression of CXCR3 on mTregs (CXCR3 average baseline MFI 2252 (45.08; 1783–3193) *n* = 35). (C and D) The increase in CCR6 expression by mTregs was maximal and dose dependent on Day 1 (CCR6 MFI 1523 (31; 1106–1973) *n* = 37). MFI, mean fluorescence intensity; Treg, regulatory T cell.

CXCR3 and CCR6 are chemokine receptors that contribute to the trafficking of T cells between the circulation and tissues [50]. In regard to the proportion of mTregs expressing CXCR3 and CCR6, we found no differences in response to aldesleukin between chemokine-defined subsets of Tregs (S13 and S14 Figs). However, the expression of CXCR3 was increased on a per cell basis on mTregs in a dose-dependent manner, peaking at day 1, and maintained for greater than 4 d at the two highest doses (*n* = 35, dose *p* = 4 × 10^−3^; Figs 10A, 10B and S14). CCR6 on mTregs also increased, peaking at day one (*n* = 37, *p* = 1.48 × 10^−4^), and was sustained above baseline for greater than 7 d at the higher doses (Figs 10C, 10D and S14).

## Discussion

Our aim is to develop an immunomodulatory therapy for T1D that increases immune regulation within physiological levels to inhibit autoreactive T cells while preserving immune responses to pathogens and cancer immunosurveillance. Such a therapy could be tested initially in newly diagnosed patients, many of whom retain sufficient insulin production to prevent diabetic complications [51,52], and, if successful, lead to the treatment of autoimmunity to prevent the metabolic diagnosis of T1D. Critical to this strategy is to first characterise the Treg dose response, the duration, and the effects of a single ultra-low dose of aldesleukin on the human immune system. In DILT1D we have determined the Treg dose response to a single dose of subcutaneous aldesleukin and defined two doses required to increase Treg frequencies by predefined amounts, in a formal clinical governance framework, with an adaptive statistical design.

The single dose of aldesleukin was well tolerated other than a small self-limiting injection site reaction, a common adverse event observed in almost every treated participant except in one, in whom there was no increase in plasma aldesleukin at 90 min following administration of an extremely low volume dose (0.02 ml). The influenza-like syndrome (malaise, myalgia, arthralgia, shivering, or fever) that has been observed in participants administered 5-d or daily dosing regimens was not recorded in DILT1D [36,53], although there were two episodes of rhinitis that were possibly related to drug administration. A norovirus infection developed following treatment in a single participant who, retrospectively, was found to have been subclinically infected before treatment and who fully recovered, mounting a robust immune response to the virus. The early time point and intensive daily monitoring in DILT1D enabled us to establish that lymphocyte counts have a transient dose-dependent decline on day 1 without the development of the persistent lymphopenia that is characteristic of the high-dose aldesleukin oncology protocols [54]. Persistent lymphopenia would not be a favourable feature of a repeat dosing regimen, but in DILT1D we observed that the decrease in lymphocytes was dose dependent, and a transient asymptomatic lymphopenia (count < 1 × 10^9^/l) was observed in only a single participant treated with the highest dose administered (1.5 × 10^6^ IU/m^2^). The two doses that DILT1D identified as increasing Tregs by 10% and 20% are ultra-low and have a low risk of lymphopenia. In contrast, eosinophils increased on day 1 after treatment, and analysis of eosinophil counts determined that the pretreatment count influenced the eosinophil response to aldesleukin, with high baseline counts leading to a transient eosinophilia post-treatment. This finding suggests that in future trials, participants with lower eosinophil counts could be stratified to aldesleukin treatment to avoid eosinophilia.

DILT1D was a mechanistic trial designed to determine immune outcomes of treatment and not to assess clinical efficacy [55]. Participants had no evidence of the development of a decline in C-peptide as has been observed with frequent administration of repeat IL-2 doses with rapamycin in participants with T1D [34]. Another feature of the trial is the use of FACS analysis of whole blood rather than cryopreserved PBMCs. Fresh whole blood facilitated rapid analysis, reduced the manipulation of the samples, and allowed the analysis of the expression of surface receptors such as the chemokine receptors that might be lost during the freezing, storage, and thawing of PBMCs. However, due to the variation in T cell numbers in the blood between participants, lower numbers of T cells were available for analysis in blood samples from some participants, making it difficult to define the response in rare subsets. The DILT1D trial was limited to adults and the analysis of peripheral blood only due to blood volume requirements and ethical and practical considerations.

In achieving the two primary endpoints (10% and 20% maximum increases in Treg frequencies from baseline), the pharmacodynamics of subcutaneous aldesleukin were also analysed, revealing hyperacute decreases in the frequencies of Tregs and of other cells in the blood, with frequencies returning to, or exceeding, baseline frequencies 24 h or days later, depending on aldesleukin dose and cell type. These decreases could be due to aldesleukin enhancing retention of cells in the tissues and/or egress of cells from the circulation into the tissues.

In the first hours after administration of aldesleukin there was limited Treg selectivity, with responses from Teffs and NK cells, due to high concentrations of the drug for the first few hours, consistent with the drug’s half-life in vivo of 4–5 h following subcutaneous administration [49]. These initial IL-2 concentrations are greater than those reported in patients with sepsis [45]. When a conventional IL-2 assay was used and not the supersensitive IL-2 assay [46], we observed the peak IL-2 concentrations at 90 min, but no quantifiable readings above background after this time point were measured. Use of the much more sensitive assay allowed for the measurement of sustained physiologically active concentrations of aldesleukin up to 4 d after dosing in the case of Tregs and between 1 and 2 d after dosing for mTeffs and NK CD56^bright^ cells. Nevertheless, there is still some level of Treg selectivity, and it remains to be determined what homeostasis the immune system will reach if steady-state increases of Tregs in the order of 20%–50% are achieved by repeated aldesleukin doses, which is now being investigated in a follow-up trial, DILfrequency (NCT02265809) [56]. The finding at 24 h post-dosing that the higher doses of aldesleukin (>0.38 × 10^6^ IU/m^2^) resulted in sufficient concentrations of IL-2 to stimulate Teffs and alter their circulation and proliferation should inform the design of dosing regimens.

Another finding that impacts the design of dosing regimens came from analysis of the expression of the IL-2 receptor subunits on Tregs, NK cells, and Teffs. There was a rapid down modulation of the signal-transducing subunit, the β chain/CD122, on Tregs associated with a reduction in the sensitivity of these cells to IL-2. It is possible that in a daily dosing induction phase of IL-2 treatment, a combination of Treg desensitisation and Teff activation could increase insulitis and exacerbate T cell/cytokine-mediated destruction of β cells in T1D patients. Taken together, the results suggest a regimen with doses in the range of 0.1 × 10^6^ to 0.5 × 10^6^ IU/m^2^, and we envisage that a favourable interval might be greater than every 2 d, but probably not as much as 1 or 2 wk, if a steady-state Treg increase is the aim. Desensitisation could partly explain the non-responsiveness of Tregs in some patients receiving daily doses of aldesleukin of 1.0 × 10^6^ IU/m^2^ or more [36]. We acknowledge that regular dosing to achieve steady-state increases of Tregs of 20%–50%, if this is achievable in DILfrequency, may not prevent β cell destruction, and a phase 2 clinical trial to test efficacy will be required to answer this question.

The prolonged, dose-dependent duration of Treg response to a single dose of aldesleukin was unexpected. At the higher doses of aldesleukin, mTregs remained elevated for greater than 7 d in the absence of IL-2 plasma levels above baseline, suggesting that trafficking through tissues and exposure to self-antigens helps to sustain the Treg increase. Exposure of Tregs to IL-2 in vitro is not sufficient to cause proliferation. Activation and cell division of Tregs requires antigen and T cell receptor signalling. In mouse lymph nodes removal of either T cell receptor signalling or the IL-2 supplied from Teffs leads to reduced Treg function and uncontrolled Teffs [22].

Finally, in addition to the effects on Tregs by aldesleukin, we observed that NK cells that express high levels of CD56 and CD122 and low levels of CD25 are highly sensitive to aldesleukin in vivo. These NK CD56^bright^ cells could have immunoregulatory function, so their induction by aldesleukin may be a desirable phenotype in suppression of T-cell-mediated autoimmunity [57] and perhaps also in the regulation of the Teff responses that were observed when plasma aldesleukin concentrations were high.

Having found an aldesleukin-induced alteration in cell trafficking, we noted that a decline in T cells from the peripheral blood has also been reported in clinical trials of teplizumab (humanised non-Fc-binding antibody to anti-CD3), which has been shown to preserve C-peptide in newly diagnosed T1D participants [4,58]. This T cell response has been characterised in a humanised mouse model and was shown to require migration of CCR6^+^ CD4^+^ T cells to the gut to acquire a regulatory phenotype, including increased CCR6 expression and IL-10 production by FOXP3^+^ Tregs [59]. Endogenous IL-2 production in response to anti-CD3 treatment was reported in the original preclinical NOD mouse study [60], and plasma IL-2 was increased in a portion of participants in the clinical trials of teplizumab in T1D, accompanied by an increase of CD25^+^CD4^+^ T cells [61]. Therefore it seems possible that anti-CD3-induced Treg migration during treatment may be due in part to IL-2 production by anti-CD3-stimulated T cells in vivo.

The DILT1D trial has characterised the effects of single ultra-low doses of aldesleukin on circulating immune cells and defined two doses that increased Tregs within the normal range. The next step in our systematic experimental medicine approach is to bring these two doses forward in a second adaptive study to determine the optimal dose and frequency of administration to reach a homeostatic increase in Tregs while minimising Teff activation [56]. We do not think this can be modelled, given the pleiotropic response to aldesleukin observed, and has to be determined using an efficient statistical design, with mechanistic analyses to characterise the effects of repeated doses of aldesleukin on the immune system.

## Supporting Information

S1 Analysis Primary endpoint analysis population. (PDF)

S2 Analysis Primary endpoint—fitted models and their parameters. (PDF)

S3 Analysis Analysis of lymphocytes and eosinophil counts from Diabetes—Genes, Autoimmunity and Prevention (D-GAP) and Cambridge BioResource cohorts. (PDF)

S1 Data Supporting data for Fig 1. (XLSX)

S2 Data Supporting data for Fig 2. (XLSX)

S3 Data Supporting data for Fig 3. (XLSX)

S4 Data Supporting data for Fig 4. (XLSX)

S5 Data Supporting data for Fig 5. (XLSX)

S6 Data Supporting data for Fig 6. (XLSX)

S7 Data Supporting data for Fig 7. (XLSX)

S8 Data Supporting data for Fig 8. (XLSX)

S9 Data Supporting data for Fig 9. (XLSX)

S10 Data Supporting data for Fig 10. (XLSX)

S1 Fig DILT1D sample workflow for each participant’s trial visit. (PDF)

S2 Fig Clinical course and anti-norovirus GII.4 antibody response of norovirus-infected participant. (PDF)

S3 Fig Injection site reactions. (PDF)

S4 Fig Changes in CD8^+^ T, B, and natural killer cell counts in response to aldesleukin. (PDF)

S5 Fig Correlation between clinical and mechanistic FACS analysis of regulatory T cells. (PDF)

S6 Fig CD25 and CD122 expression on memory and naïve regulatory T cells from baseline to day 7 post-treatment. (PDF)

S7 Fig Linear increase in CD25 expression on regulatory T cells in response to increased aldesleukin dose. (PDF)

S8 Fig Effects in vitro of aldesleukin on CD25 and CD122 expression on natural killer CD56^bright^ cells, memory regulatory T cells, and effector T cells. (PDF)

S9 Fig Increased pSTAT5, CD25, and FOXP3 levels in regulatory T cell subsets in blood following a dose of aldesleukin. (PDF)

S10 Fig The effects of an aldesleukin dose on memory effector T cell frequency and CD69^+^ regulatory and effector T cells and pSTAT5 response in memory effector T cells. (PDF)

S11 Fig Natural killer cell responses to treatment and baseline expression of CD25 and CD122. (PDF)

S12 Fig Soluble CD25 and C-reactive protein responses. (PDF)

S13 Fig Changes in frequency of CXCR3^+^CCR6^+^ and CXCR3^−^CCR6^−^memory regulatory T cells. (PDF)

S14 Fig CXCR3^+^ and CCR6^+^ memory and naïve regulatory T cell responses to treatment. (PDF)

S1 Materials DILT1D standard operating procedure for flow cytometry staining and cell sorting. (PDF)

S2 Materials Mechanistic flow quality control process and missing mechanistic data summary. (PDF)

S3 Materials Dose Determining Committee statistical analysis report from the adaptive phase of DILT1D (interim report DILT1D trial). (PDF)

S1 Table Antibody combinations for surface (tubes 1–6) and intracellular staining (tube 7). (PDF)

S2 Table Detailed antibody/clone information. (PDF)

S3 Table Antibody combinations for cell sorting. (PDF)

S4 Table Antibody combinations and information for pSTAT5 assay. (PDF)

S5 Table Full blood counts baseline, day 1, and final visit. (PDF)

S6 Table Clinical FACS analysis of TBNK assay at baseline, day 1, and final visit. (PDF)

S7 Table Metabolic measures and thyroid function tests at baseline and final visit. (PDF)

S8 Table Renal, bone, and liver biochemistries at baseline and final visit. (PDF)

S1 Text DILT1D trial protocol. (PDF)

S2 Text CONSORT statement. (DOC)

S3 Text Health Research Authority—favourable ethical opinion with conditions. (PDF)

S4 Text Health Research Authority—acknowledgment of receipt of additional documentation and confirmation of ethical approval. (PDF)

## Abbreviations

AE: adverse event
AIC: Akaike information criterion
CPM: counts per minute
CRP: C-reactive protein
DDC: Dose Determining Committee
DILT1D: Adaptive Study of IL-2 Dose on Regulatory T Cells in Type 1 Diabetes
FACS: fluorescence-activated cell sorting
MFI: mean fluorescence intensity
mTeff: memory effector T cell
mTreg: memory regulatory T cell
NK: natural killer
NOD: non-obese diabetic
nTreg: naïve regulatory T cell
PBMC: peripheral blood mononuclear cell
rhIL-2: recombinant human IL-2
SE: standard error
Teff: effector T cell
Treg: regulatory T cell
T1D: type 1 diabetes

## References

[1] Atkinson MA, Eisenbarth GS, Michels AW. Type 1 diabetes. Lancet. 2014;383:69–82. 10.1016/S0140-6736(13)60591-7 23890997 PMC4380133

[2] McKnight JA, Wild SH, Lamb MJ, Cooper MN, Jones TW, Davis EA, et al Glycaemic control of Type 1 diabetes in clinical practice early in the 21st century: an international comparison. Diabet Med. 2015;32:1036–1050. 10.1111/dme.12676 25510978

[3] Miller KM, Foster NC, Beck RW, Bergenstal RM, DuBose SN, DiMeglio LA, et al Current state of type 1 diabetes treatment in the U.S.: updated data from the T1D Exchange clinic registry. Diabetes Care. 2015;38:971–978. 10.2337/dc15-0078 25998289

[4] Herold KC, Gitelman SE, Willi SM, Gottlieb PA, Waldron-Lynch F, Devine L, et al Teplizumab treatment may improve C-peptide responses in participants with type 1 diabetes after the new-onset period: a randomised controlled trial. Diabetologia. 2013;56:391–400. 10.1007/s00125-012-2753-4 23086558 PMC3537871

[5] Pescovitz MD, Greenbaum CJ, Krause-Steinrauf H, Becker DJ, Gitelman SE, Goland R, et al Rituximab, B-lymphocyte depletion, and preservation of beta-cell function. N Engl J Med. 2009;361:2143–2152. 10.1056/NEJMoa0904452 19940299 PMC6410357

[6] Keymeulen B, Vandemeulebroucke E, Ziegler AG, Mathieu C, Kaufman L, Hale G, et al Insulin needs after CD3-antibody therapy in new-onset type 1 diabetes. N Engl J Med. 2005;352:2598–2608. 10.1056/NEJMoa043980 15972866

[7] Rigby MR, Harris KM, Pinckney A, DiMeglio LA, Rendell MS, Felner EI, et al Alefacept provides sustained clinical and immunological effects in new-onset type 1 diabetes patients. J Clin Invest. 2015;125:3285–3296. 10.1172/JCI81722 26193635 PMC4623571

[8] Orban T, Bundy B, Becker DJ, DiMeglio LA, Gitelman SE, Goland R, et al Co-stimulation modulation with abatacept in patients with recent-onset type 1 diabetes: a randomised, double-blind, placebo-controlled trial. Lancet. 2011;378:412–419. 10.1016/S0140-6736(11)60886-6 21719096 PMC3462593

[9] Nelson MR, Tipney H, Painter JL, Shen J, Nicoletti P, Shen Y, et al The support of human genetic evidence for approved drug indications. Nat Genet. 2015;47:856–860. 10.1038/ng.3314 26121088

[10] Cho JH, Feldman M. Heterogeneity of autoimmune diseases: pathophysiologic insights from genetics and implications for new therapies. Nat Med. 2015;21:730–738. 10.1038/nm.3897 26121193 PMC5716342

[11] Todd JA, Aitman TJ, Cornall RJ, Ghosh S, Hall JR, Hearne CM, et al Genetic analysis of autoimmune type 1 diabetes mellitus in mice. Nature. 1991;351:542–547. 10.1038/351542a0 1675432

[12] Yamanouchi J, Rainbow D, Serra P, Howlett S, Hunter K, Garner VE, et al Interleukin-2 gene variation impairs regulatory T cell function and causes autoimmunity. Nat Genet. 2007;39:329–337. 10.1038/ng1958 17277778 PMC2886969

[13] Vella A, Cooper JD, Lowe CE, Walker N, Nutland S, Widmer B, et al Localization of a type 1 diabetes locus in the IL2RA/CD25 region by use of tag single-nucleotide polymorphisms. Am J Hum Genet. 2005;76:773–779. 10.1086/429843 15776395 PMC1199367

[14] Lowe CE, Cooper JD, Brusko T, Walker NM, Smyth DJ, Bailey R, et al Large-scale genetic fine mapping and genotype-phenotype associations implicate polymorphism in the IL2RA region in type 1 diabetes. Nat Genet. 2007;39:1074–1082. 10.1038/ng2102 17676041

[15] Todd JA, Walker NM, Cooper JD, Smyth DJ, Downes K, Plagnol V, et al Robust associations of four new chromosome regions from genome-wide analyses of type 1 diabetes. Nat Genet. 2007;39:857–864. 10.1038/ng2068 17554260 PMC2492393

[16] Dendrou CA, Plagnol V, Fung E, Yang JH, Downes K, Cooper JD, et al Cell-specific protein phenotypes for the autoimmune locus IL2RA using a genotype-selectable human bioresource. Nat Genet. 2009;41:1011–1015. 10.1038/ng.434 19701192 PMC2749506

[17] Garg G, Tyler JR, Yang JH, Cutler AJ, Downes K, Pekalski M, et al Type 1 diabetes-associated IL2RA variation lowers IL-2 signaling and contributes to diminished CD4+CD25+ regulatory T cell function. J Immunol. 2012;188:4644–4653. 10.4049/jimmunol.1100272 22461703 PMC3378653

[18] Thompson WS, Pekalski ML, Simons HZ, Smyth DJ, Castro-Dopico X, Guo H, et al Multi-parametric flow cytometric and genetic investigation of the peripheral B cell compartment in human type 1 diabetes. Clin Exp Immunol. 2014;177:571–585. 10.1111/cei.12362 24773525 PMC4137841

[19] Boyman O, Sprent J. The role of interleukin-2 during homeostasis and activation of the immune system. Nat Rev Immunol. 2012;12:180–190. 10.1038/nri3156 22343569

[20] Liao W, Lin JX, Leonard WJ. Interleukin-2 at the crossroads of effector responses, tolerance, and immunotherapy. Immunity. 2013;38:13–25. 10.1016/j.immuni.2013.01.004 23352221 PMC3610532

[21] Liston A, Gray DH. Homeostatic control of regulatory T cell diversity. Nat Rev Immunol. 2014;14:154–165. 10.1038/nri3605 24481337

[22] Liu Z, Gerner MY, Van Panhuys N, Levine AG, Rudensky AY, Germain RN. Immune homeostasis enforced by co-localized effector and regulatory T cells. Nature. 2015;528:225–230. 10.1038/nature16169 26605524 PMC4702500

[23] Setoguchi R, Hori S, Takahashi T, Sakaguchi S. Homeostatic maintenance of natural Foxp3(+) CD25(+) CD4(+) regulatory T cells by interleukin (IL)-2 and induction of autoimmune disease by IL-2 neutralization. J Exp Med. 2005;201:723–735. 10.1084/jem.20041982 15753206 PMC2212841

[24] Atkins MB, Lotze MT, Dutcher JP, Fisher RI, Weiss G, Margolin K, et al High-dose recombinant interleukin 2 therapy for patients with metastatic melanoma: analysis of 270 patients treated between 1985 and 1993. J Clin Oncol. 1999;17:2105–2116. 10561265 10.1200/JCO.1999.17.7.2105

[25] Negrier S, Escudier B, Lasset C, Douillard JY, Savary J, Chevreau C, et al Recombinant human interleukin-2, recombinant human interferon alfa-2a, or both in metastatic renal-cell carcinoma. Groupe Français d’Immunothérapie. N Engl J Med. 1998;338:1272–1278. 10.1056/NEJM199804303381805 9562581

[26] Sim GC, Martin-Orozco N, Jin L, Yang Y, Wu S, Washington E, et al IL-2 therapy promotes suppressive ICOS+ Treg expansion in melanoma patients. J Clin Invest. 2014;124:99–110. 10.1172/JCI46266 24292706 PMC3871216

[27] Tang Q, Adams JY, Penaranda C, Melli K, Piaggio E, Sgouroudis E, et al Central role of defective interleukin-2 production in the triggering of islet autoimmune destruction. Immunity. 2008;28:687–697. 10.1016/j.immuni.2008.03.016 18468463 PMC2394854

[28] Grinberg-Bleyer Y, Baeyens A, You S, Elhage R, Fourcade G, Gregoire S, et al IL-2 reverses established type 1 diabetes in NOD mice by a local effect on pancreatic regulatory T cells. J Exp Med. 2010;207:1871–1878. 10.1084/jem.20100209 20679400 PMC2931175

[29] Koreth J, Matsuoka K, Kim HT, McDonough SM, Bindra B, Alyea EP, et al Interleukin-2 and regulatory T cells in graft-versus-host disease. N Engl J Med. 2011;365:2055–2066. 10.1056/NEJMoa1108188 22129252 PMC3727432

[30] Koreth J, Kim HT, Jones KT, Lange PB, Reynolds CG, Chammas MJ, et al Efficacy, durability, and response predictors of low-dose interleukin-2 therapy for chronic graft vs. host disease. Blood. 2016;128:130–137. 10.1182/blood-2016-02-702852 27073224 PMC4937358

[31] Saadoun D, Rosenzwajg M, Joly F, Six A, Carrat F, Thibault V, et al Regulatory T-cell responses to low-dose interleukin-2 in HCV-induced vasculitis. N Engl J Med. 2011;365:2067–2077. 10.1056/NEJMoa1105143 22129253

[32] Humrich JY, von Spee-Mayer C, Siegert E, Alexander T, Hiepe F, Radbruch A, et al Rapid induction of clinical remission by low-dose interleukin-2 in a patient with refractory SLE. Ann Rheum Dis. 2015;74:791–792. 10.1136/annrheumdis-2014-206506 25609413

[33] Castela E, Le Duff F, Butori C, Ticchioni M, Hofman P, Bahadoran P, et al Effects of low-dose recombinant interleukin 2 to promote T-regulatory cells in alopecia areata. JAMA Dermatol. 2014;150:748–751. 10.1001/jamadermatol.2014.504 24872229

[34] Long SA, Rieck M, Sanda S, Bollyky JB, Samuels PL, Goland R, et al Rapamycin/IL-2 combination therapy in patients with type 1 diabetes augments Tregs yet transiently impairs β-cell function. Diabetes. 2012 10.2337/db12-0049 PMC3425404 22721971

[35] Long SA, Buckner JH, Greenbaum CJ. IL-2 therapy in type 1 diabetes: “Trials” and tribulations. Clin Immunol. 2013;149:324–331. 10.1016/j.clim.2013.02.005 23499139

[36] Hartemann A, Bensimon G, Payan CA, Jacqueminet S, Bourron O, Nicolas N, et al Low-dose interleukin 2 in patients with type 1 diabetes: a phase 1/2 randomised, double-blind, placebo-controlled trial. Lancet Diabetes Endocrinol. 2013;1:295–305. 10.1016/S2213-8587(13)70113-X 24622415

[37] Low-dose rhIL-2 in patients with recently-diagnosed type 1 diabetes (DIABIL-2). ClinicalTrials.gov. 2016 Feb 15 [cited 26 Jul 2016]. Available: https://clinicaltrials.gov/ct2/show/NCT02411253.

[38] Staeva-Vieira T, Peakman M, von Herrath M. Translational mini-review series on type 1 diabetes: immune-based therapeutic approaches for type 1 diabetes. Clin Exp Immunol. 2007;148:17–31. 10.1111/j.1365-2249.2007.03328.x 17349010 PMC1868847

[39] Lernmark A, Larsson HE. Immune therapy in type 1 diabetes mellitus. Nat Rev Endocrinol. 2013;9:92–103. 10.1038/nrendo.2012.237 23296174

[40] Kenefeck R, Wang CJ, Kapadi T, Wardzinski L, Attridge K, Clough LE, et al Follicular helper T cell signature in type 1 diabetes. J Clin Invest. 2015;125:292–303. 10.1172/JCI76238 25485678 PMC4382272

[41] Ferreira RC, Simons HZ, Thompson WS, Cutler AJ, Dopico XC, Smyth DJ, et al IL-21 production by CD4+ effector T cells and frequency of circulating follicular helper T cells are increased in type 1 diabetes patients. Diabetologia. 2015;58:781–790. 10.1007/s00125-015-3509-8 25652388 PMC4351433

[42] Waldron-Lynch F, Kareclas P, Irons K, Walker NM, Mander A, Wicker LS, et al Rationale and study design of the Adaptive study of IL-2 dose on regulatory T cells in type 1 diabetes (DILT1D): a non-randomised, open label, adaptive dose finding trial. BMJ Open. 2014;4:e005559 10.1136/bmjopen-2014-005559 24898091 PMC4054640

[43] Yang JH, Cutler AJ, Ferreira RC, Reading JL, Cooper NJ, Wallace C, et al Natural variation in interleukin-2 sensitivity influences regulatory T-cell frequency and function in individuals with long-standing type 1 diabetes. Diabetes. 2015;64:3891–3902. 10.2337/db15-0516 26224887 PMC4975524

[44] Rainbow DB, Yang X, Burren O, Pekalski ML, Smyth DJ, Klarqvist MD, et al Epigenetic analysis of regulatory T cells using multiplex bisulfite sequencing. Eur J Immunol. 2015;45:3200–3203. 10.1002/eji.201545646 26420295 PMC4832369

[45] Glezer EN, Stengelin M, Aghvanyan A, Nikolenko GN, Roy D, Higgins M, et al. Cytokine immunoassays with sub-fg/ml detection limits. AAPS 2014 National Biotechnology Conference; 19–21 May 2014; San Diego, CA, US.

[46] Bell CJ, Sun Y, Nowak UM, Clark J, Howlett S, Pekalski ML, et al Sustained in vivo signaling by long-lived IL-2 induces prolonged increases of regulatory T cells. J Autoimmun. 2015;56:66–80. 10.1016/j.jaut.2014.10.002 25457307 PMC4298360

[47] Downes K, Marcovecchio ML, Clarke P, Cooper JD, Ferreira RC, Howson JM, et al Plasma concentrations of soluble IL-2 receptor α (CD25) are increased in type 1 diabetes and associated with reduced C-peptide levels in young patients. Diabetologia. 2014;57:366–372. 10.1007/s00125-013-3113-8 24264051 PMC3890035

[48] Heywood J, Evangelou M, Goymer D, Kennet J, Anselmiova K, Guy C, et al Effective recruitment of participants to a phase I study using the internet and publicity releases through charities and patient organisations: analysis of the adaptive study of IL-2 dose on regulatory T cells in type 1 diabetes (DILT1D). Trials. 2015;16:86 10.1186/s13063-015-0583-7 25881192 PMC4369347

[49] Konrad MW, Hemstreet G, Hersh EM, Mansell PW, Mertelsmann R, Kolitz JE, et al Pharmacokinetics of recombinant interleukin 2 in humans. Cancer Res. 1990;50:2009–2017. 2317789

[50] Duhen T, Duhen R, Lanzavecchia A, Sallusto F, Campbell DJ. Functionally distinct subsets of human FOXP3+ Treg cells that phenotypically mirror effector Th cells. Blood. 2012;119:4430–4440. 10.1182/blood-2011-11-392324 22438251 PMC3362361

[51] The Diabetes Control and Complications Trial Research Group. The effect of intensive treatment of diabetes on the development and progression of long-term complications in insulin-dependent diabetes mellitus. N Engl J Med. 1993;329:977–986. 8366922 10.1056/NEJM199309303291401

[52] Kuhtreiber WM, Washer SL, Hsu E, Zhao M, Reinhold P, Burger D, et al Low levels of C-peptide have clinical significance for established Type 1 diabetes. Diabet Med. 2015;32:1346–1353. 10.1111/dme.12850 26172028 PMC4578991

[53] Ito S, Bollard CM, Carlsten M, Melenhorst JJ, Biancotto A, Wang E, et al Ultra-low dose interleukin-2 promotes immune-modulating function of regulatory T cells and natural killer cells in healthy volunteers. Mol Ther. 2014;22:1388–1395. 10.1038/mt.2014.50 24686272 PMC4089007

[54] Electronic Medicines Compendium. Proleukin. 2015 Jan 20 [cited 17 Mar 2016]. Available: http://www.medicines.org.uk/emc/medicine/19322.

[55] Itano AA, Sims MJ, Siu G. Mechanistic medicine: novel strategies for clinical trials. Autoimmunity. 2010;43:560–571. 10.3109/08916931003674782 20429849

[56] Truman LA, Pekalski ML, Kareclas P, Evangelou M, Walker NM, Howlett J, et al Protocol of the adaptive study of IL-2 dose frequency on regulatory T cells in type 1 diabetes (DILfrequency): a mechanistic, non-randomised, repeat dose, open-label, response-adaptive study. BMJ Open. 2015;5:e009799 10.1136/bmjopen-2015-009799 26646829 PMC4679899

[57] Bielekova B, Catalfamo M, Reichert-Scrivner S, Packer A, Cerna M, Waldmann TA, et al Regulatory CD56(bright) natural killer cells mediate immunomodulatory effects of IL-2Ralpha-targeted therapy (daclizumab) in multiple sclerosis. Proc Natl Acad Sci U S A. 2006;103:5941–5946. 10.1073/pnas.0601335103 16585503 PMC1458677

[58] Herold KC, Gitelman SE, Ehlers MR, Gottlieb PA, Greenbaum CJ, Hagopian W, et al Teplizumab (anti-CD3 mAb) treatment preserves C-peptide responses in patients with new-onset type 1 diabetes in a randomized controlled trial: metabolic and immunologic features at baseline identify a subgroup of responders. Diabetes. 2013;62:3766–3774. 10.2337/db13-0345 23835333 PMC3806618

[59] Waldron-Lynch F, Henegariu O, Deng S, Preston-Hurlburt P, Tooley J, Flavell R, et al Teplizumab induces human gut-tropic regulatory cells in humanized mice and patients. Sci Transl Med. 2012;4:118ra12 10.1126/scitranslmed.3003401 22277969 PMC4131554

[60] Chatenoud L, Primo J, Bach JF. CD3 antibody-induced dominant self tolerance in overtly diabetic NOD mice. J Immunol. 1997;158:2947–2954. 9058834

[61] Herold KC, Burton JB, Francois F, Poumian-Ruiz E, Glandt M, Bluestone JA. Activation of human T cells by FcR nonbinding anti-CD3 mAb, hOKT3gamma1(Ala-Ala). J Clin Invest. 2003;111:409–418. 10.1172/JCI16090 12569167 PMC151852

