# Supplementary material for: Regulatory T Cell Responses in Participants with Type 1 Diabetes after a Single Dose of Interleukin-2: A Non-Randomised, Open Label, Adaptive Dose-Finding Trial
Source: PLoS Med. 2016 Oct 11;13(10):e1002139. doi: 10.1371/journal.pmed.1002139 (PMC5058548; doi:10.1371/journal.pmed.1002139)
Supplement: S1 Analysis — (PDF) [file pmed.1002139.s001.pdf]

S1 analysis Primary Endpoint Analysis Population

Primary Endpoint Analysis

Analysis Population

The primary endpoint defined as the maximum percentage change observed over the profile of 7 days for the 40 individuals of the analysis population. The average value of the primary endpoint was 18.35 (0.36) and ranged between -1.408 and 72.5.

Three of the tested models converged:

i) Linear Model: $y = 0.102\ (0.0295) + 0.197\ (0.0532)\ dose$

ii) Quadratic Model: $y = 0.0707\ (0.0366) + 0.383\ (0.141)\ dose - 0.146\ (0.102)\ dose^2$

iii) Cubic Model: $y = 0.0586\ (0.0448) + 0.548\ (0.372)\ dose – 0.485\ (0.711)\ dose^2 + 0.159\ (0.331)\ dose^3$

The estimated doses of the three models that achieve the two selected targets of 10% and 20% are:

Model/ Taget	10%	20%
Linear	-0.0123 (0.152)	0.496 (0.103)
Quadratic	0.0788 (0.0807)	0.398 (0.0889)
Cubic	0.0813 (0.0618)	0.358 (0.123)

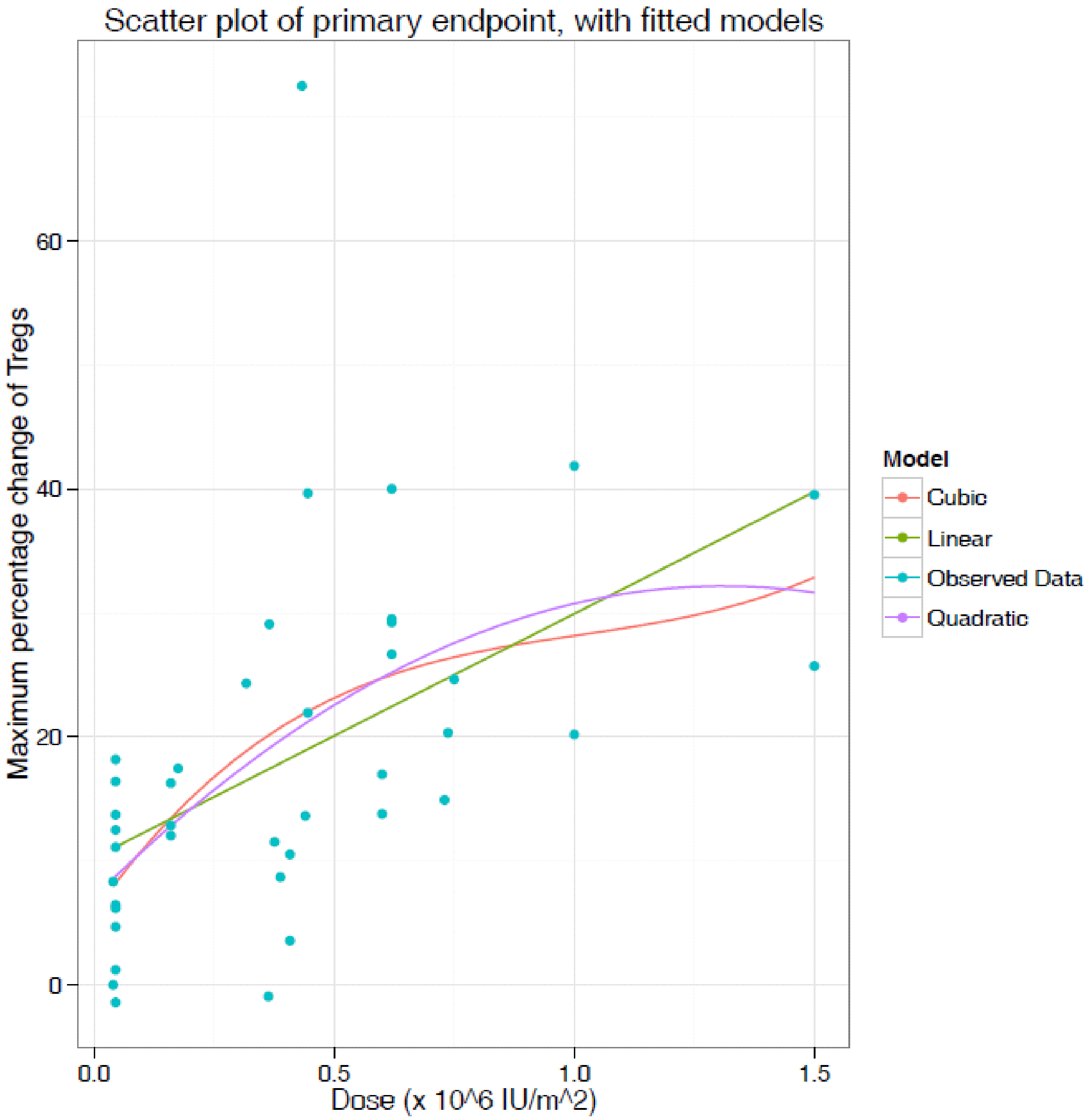
Scatter plot of primary endpoint, with fitted models
Maximum percentage change of Tregs
60
40
20
0
0.0
0.5
1.0
1.5
Dose (x 10^6 IU/m^2)
Model
Cubic
Linear
Observed Data
Quadratic
