# Supplementary material for: Regulatory T Cell Responses in Participants with Type 1 Diabetes after a Single Dose of Interleukin-2: A Non-Randomised, Open Label, Adaptive Dose-Finding Trial
Source: PLoS Med. 2016 Oct 11;13(10):e1002139. doi: 10.1371/journal.pmed.1002139 (PMC5058548; doi:10.1371/journal.pmed.1002139)

S2 analysis

Title: Primary endpoint - Fitted models and their parameters
Number: 3.10
Population: Evaluable

Model	a (SE)	b (SE)	c (SE)	d (SE)	Dose P-value	Deviance	AIC	Residual Error
Linear	0.090 (0.021)	0.195 (0.038)			1.0144e-05	0.297	-73.588	0.090
Quadratic	0.070 (0.026)	0.311 (0.102)	-0.091 (0.075)		3.2341e-05	0.285	-73.186	0.089
Emax	0.068 (0.031)	0.360 (0.214)	1.512 (1.978)		3.3965e-05	0.286	-73.080	0.089
Cubic	0.074 (0.033)	0.256 (0.274)	0.022 (0.522)	-0.053 (0.242)	0.00013872	0.284	-71.239	0.090
Logistic	-0.039 (0.505)	0.383 (0.595)	0.309 (0.900)	0.366 (0.611)	0.00014077	0.285	-71.206	0.090
Emax4	0.074 (0.055)	0.419 (0.673)	1.022 (3.574)	0.174 (1.463)	0.00014814	0.286	-71.088	0.090

Title: Primary endpoint - Predicted dose estimates
Number: 3.11
Population: Evaluable

Model	Estimate (SE) - 10% Target	95% CI - 10% Target	Estimate (SE) - 20% Target	95% CI - 20% Target
Linear	0.054 (0.102)	-0.145 - 0.253	0.567 (0.080)	0.411 - 0.723
Quadratic	0.099 (0.068)	-0.034 - 0.232	0.487 (0.087)	0.317 - 0.658
Emax	0.095 (0.062)	-0.026 - 0.217	0.486 (0.098)	0.293 - 0.679
Cubic	0.101 (0.078)	-0.052 - 0.254	0.497 (0.092)	0.316 - 0.678
Logistic	0.104 (0.086)	-0.066 - 0.273	0.494 (0.091)	0.316 - 0.673
Emax4	0.101 (0.098)	-0.091 - 0.293	0.487 (0.097)	0.297 - 0.678

Title: Scatter plot with fitted models illustrated
Number: 3.13
Population: Evaluable

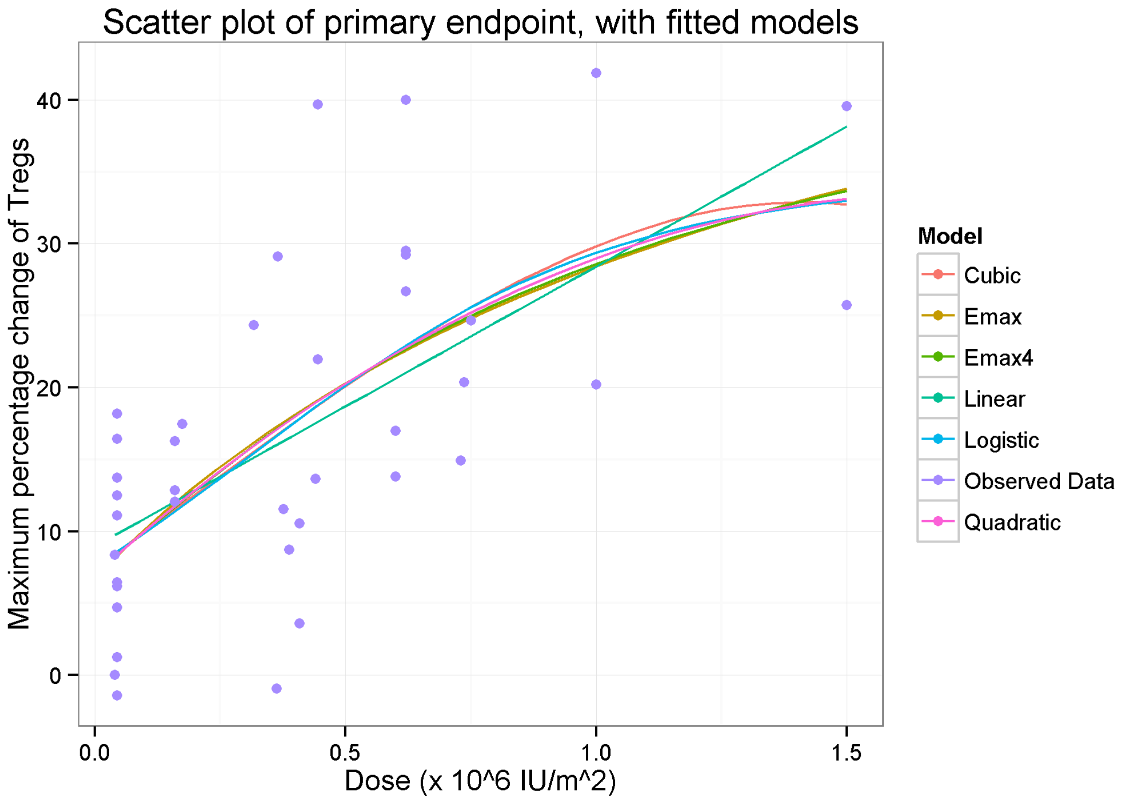

Supplement: S2 Analysis — (PDF) [file pmed.1002139.s002.pdf]
