# Supplementary material for: Regulatory T Cell Responses in Participants with Type 1 Diabetes after a Single Dose of Interleukin-2: A Non-Randomised, Open Label, Adaptive Dose-Finding Trial
Source: PLoS Med. 2016 Oct 11;13(10):e1002139. doi: 10.1371/journal.pmed.1002139 (PMC5058548; doi:10.1371/journal.pmed.1002139)
Supplement: S3 Analysis — (PDF) [file pmed.1002139.s003.pdf]

S3 analysis

Analysis of lymphocytes and eosinophil counts from Diabetes - genes, autoimmunity and prevention (**D-GAP**) and Cambridge BioResource (**CBR**) DIL cohorts

Suppose that $X_{1,\ \dots,\ N}$ and $Y_{1,\ \dots,\ N}$ refer to the baseline measurement and a measurement at a time point j of a response of interest for participants 1, …, N, respectively. As discussed in the Materials and Methods section, the absolute change at the time point j is defined as the difference between the measurement at the time point j and baseline ($D_{1,\ \dots,\ N} = Y_{1,\ \dots,\ N} - X_{1,\ \dots,\ N}$). The correlation between the absolute change and the baseline measurements is defined as:

$$\text{cor}(\mathbf{D},\mathbf{X}) = \text{cor}(\mathbf{Y}-\mathbf{X}, \mathbf{X}) = \text{cor}(\mathbf{Y},\mathbf{X}) - \text{cor}(\mathbf{X},\mathbf{X}) = \text{cor}(\mathbf{Y},\mathbf{X}) - 1$$

As the correlation between two random variables lies between -1 and 1 this implies that the cor (D,X) lies between -1 and 0. These results suggest that we are expecting the coefficient of baseline in the analysis of the absolute change of the response of interest to be negative.

The full blood count (FBC) datasets come from two observational studies investigating T1D: "Diabetes - Genes, Autoimmunity and Phenotype (**D-GAP**)" (UKCRN5799) and "Genes and Mechanisms in the Type 1 Diabetes in the Cambridge Bioresource" (**CBR**) (Table S3.1). They include adults and children (less than 18 years old) as well as T1D patients and controls. Median time differences between first and second measurement were 112 days (IQR 91-161) for D-GAP and 257 days (IQR 120-688) for CBR.

Table S3.1. Summary of independent cohort demographics

	No. Samples	No. individuals	T1D/ Controls	Sex (F/M)	Age (Children/ Adults)
D-GAP	204	102	94/ 8	45/ 57	89/ 13
CBR	374	187	6/ 181	113/ 74	2/ 185

For these two populations we have plotted the absolute change of their second measurement from their first measurement of eosinophils and lymphocytes against their first measurement (**Figure S3.1**). In DILT1D, we were interested to test how the absolute change of eosinophils (**Figure S3.2**) and lymphocytes (**Figure S3.3**) depend on their corresponding baseline at different time points: 90 minutes post administration, Day 1, Day 2 and for their final visit.

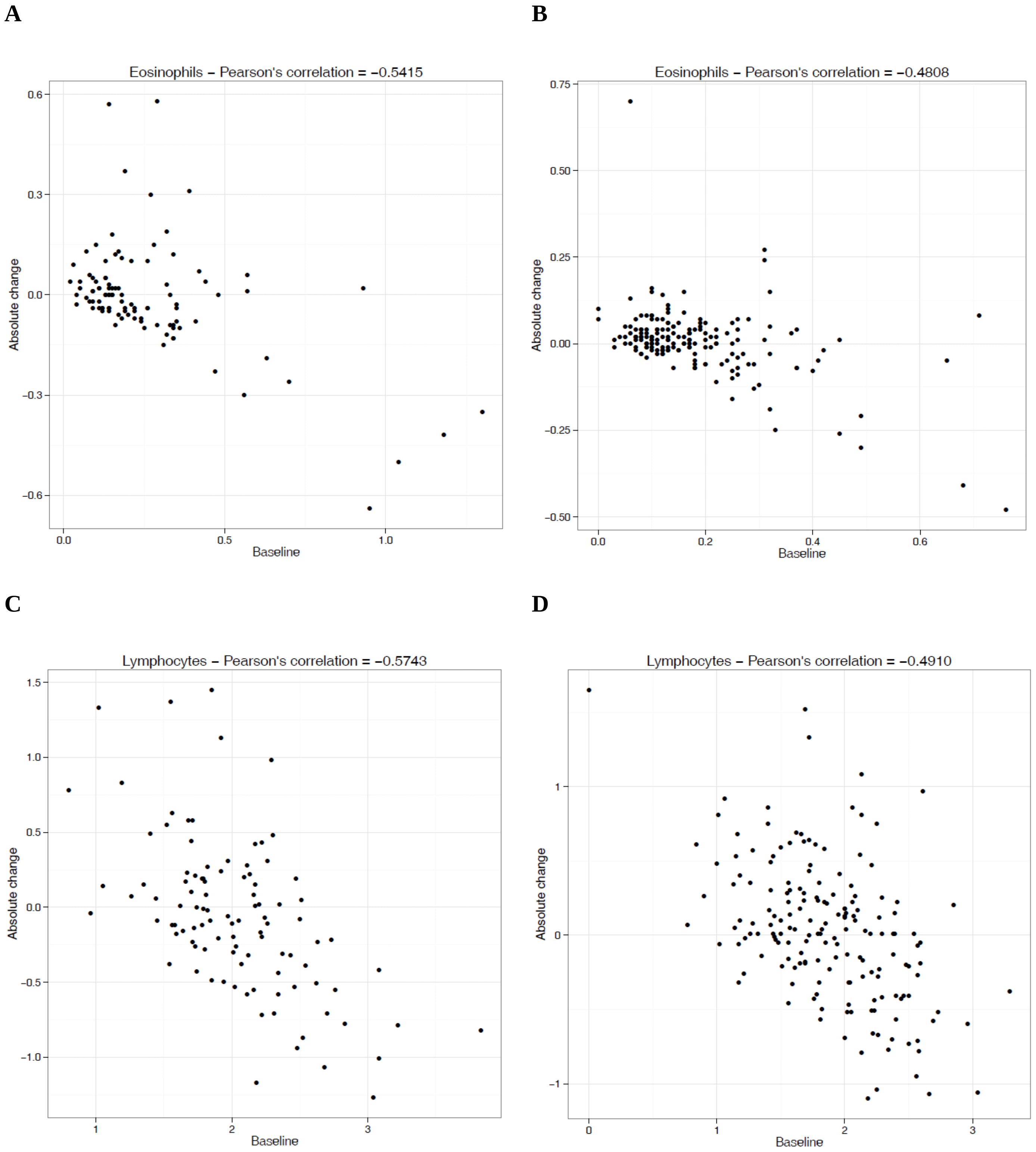

Figure S3.1. Plots of absolute change in a second measurement in eosinophil count D-GAP (A) and CBR (B) lymphocyte count (bottom panel) in two independent cohorts: D-GAP (C) and CBR (D).

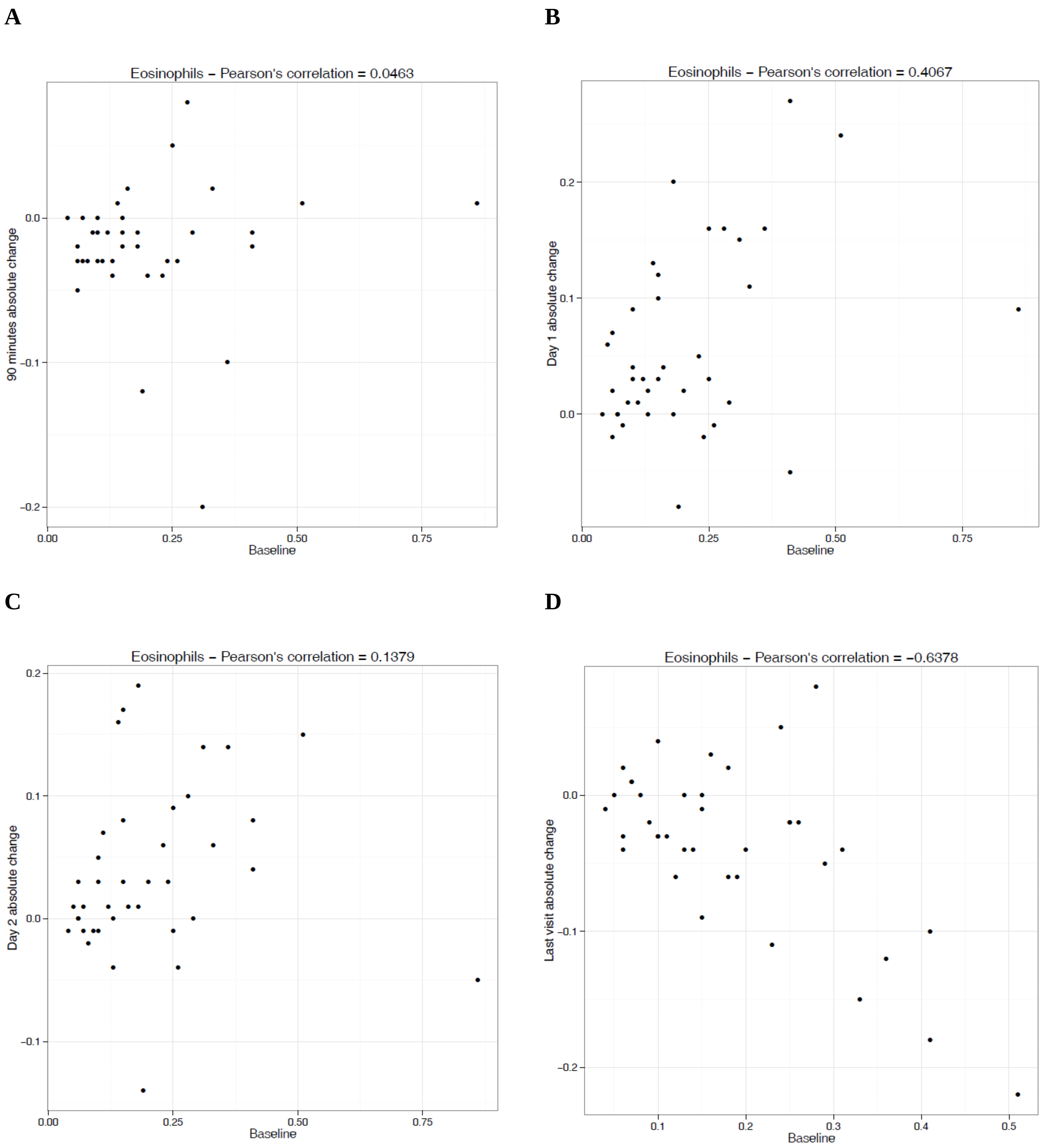

Figure S3.2. Plots of eosinophil counts for DILT1D participants at baseline against counts at 90 minutes (**A**), Day 1 (**B**), Day 2 (**C**) and the last visit (**D**)

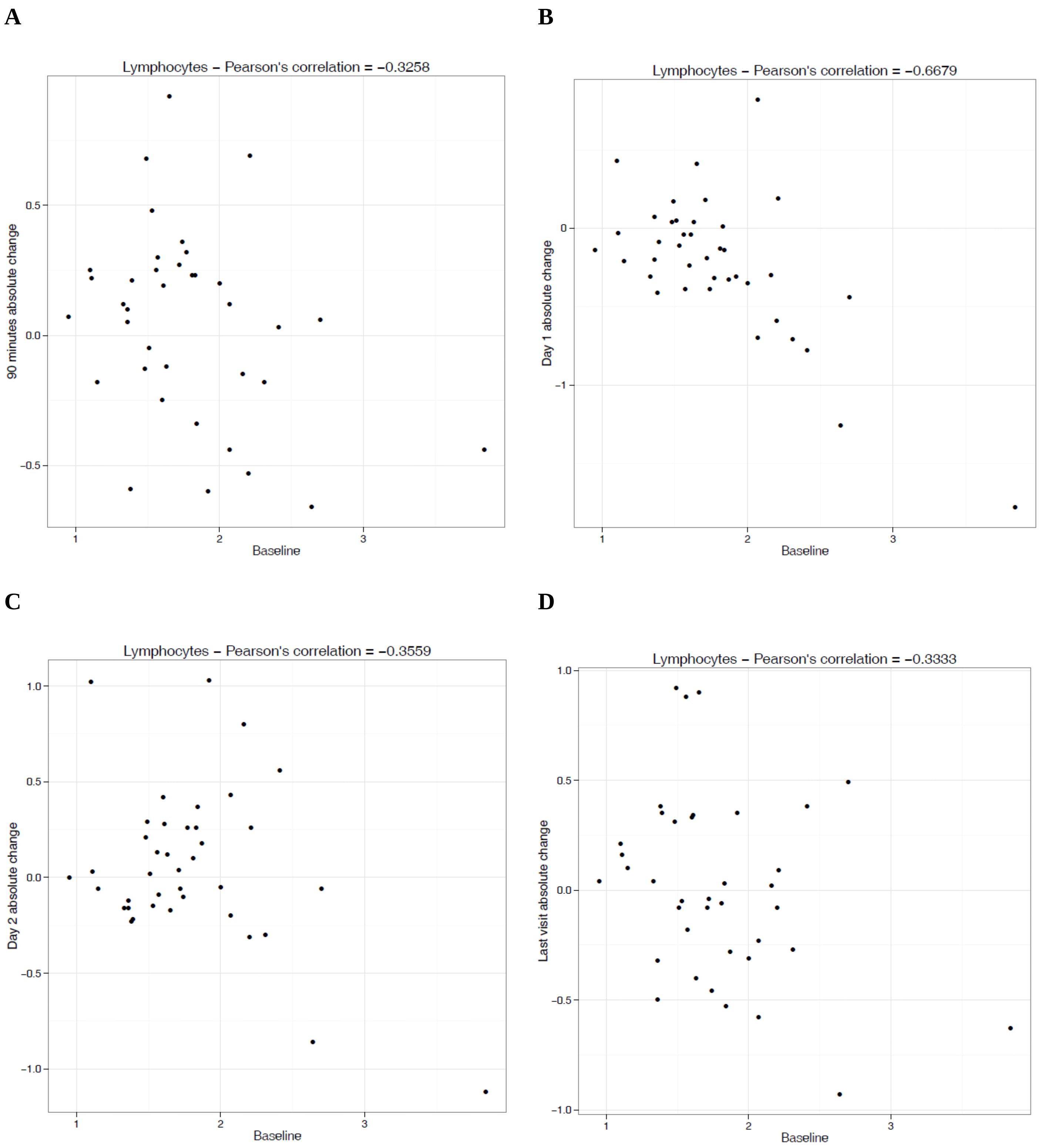

Figure S3.3. Plots of lymphocyte counts for DILT1D participants at baseline against counts at 90 minutes (**A**), Day 1 (**B**), Day 2 (**C**) and the last visit (**D**)

As can be seen, the correlation of the absolute change of eosinophils for the first 3 data points with its baseline in DILT1D participants (**Figure S3.2** A-C) is positive. This is not expected based on the theoretical result (**Figure S3.1 –** top panel) and indicates a genuine relationship between baseline and the absolute change of eosinophils at the 90 minutes, Day 1 and Day 2 time points.

On the other hand, the correlation between the absolute change of lymphocytes and its baseline is negative for all four reported measurements (**Figure S3.3**), in keeping with that seen in the two independent cohorts (**Figure S3.1 –** bottom panel). However, a set of candidate models that included the effects of both baseline lymphocyte count and dose was fitted and we found that the change in lymphocyte counts was found to be associated with dose through an interaction model that depends both on baseline and dose
