# Supplementary material for: Regulatory T Cell Responses in Participants with Type 1 Diabetes after a Single Dose of Interleukin-2: A Non-Randomised, Open Label, Adaptive Dose-Finding Trial
Source: PLoS Med. 2016 Oct 11;13(10):e1002139. doi: 10.1371/journal.pmed.1002139 (PMC5058548; doi:10.1371/journal.pmed.1002139)
Supplement: S1 Fig — (PDF) [file pmed.1002139.s014.pdf]

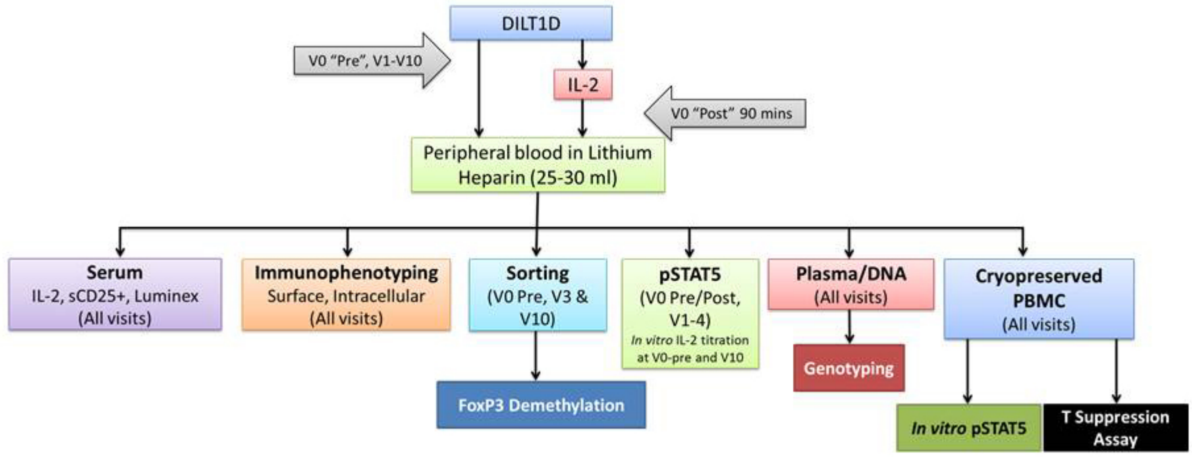

S1 Fig. DILT1D sample workflow for each participant's trial visit. Cell sorting was carried out in a subset of patients on Visits 0 (V0, pre-treatment), V3 and V10. The pSTAT5 whole blood assay to assess IL-2 signalling *in vivo* was performed on V0, Post (90 minutes), V1, V2, V3 and V4. At the highest doses of 1-1.5 x $10^6/m^2$, day 7 post-treatment was also assessed since pSTAT5 levels had not fallen to baseline by V4. *In vitro* IL-2 titration was carried out on V0 (N=21) and V10 (Day 60, N=39). No difference was observed in the dose response to IL-2 at V0 versus V10 in 39 participants where both time points were assessed.
