# Supplementary material for: Regulatory T Cell Responses in Participants with Type 1 Diabetes after a Single Dose of Interleukin-2: A Non-Randomised, Open Label, Adaptive Dose-Finding Trial
Source: PLoS Med. 2016 Oct 11;13(10):e1002139. doi: 10.1371/journal.pmed.1002139 (PMC5058548; doi:10.1371/journal.pmed.1002139)
Supplement: S2 Fig — (PDF) [file pmed.1002139.s015.pdf]

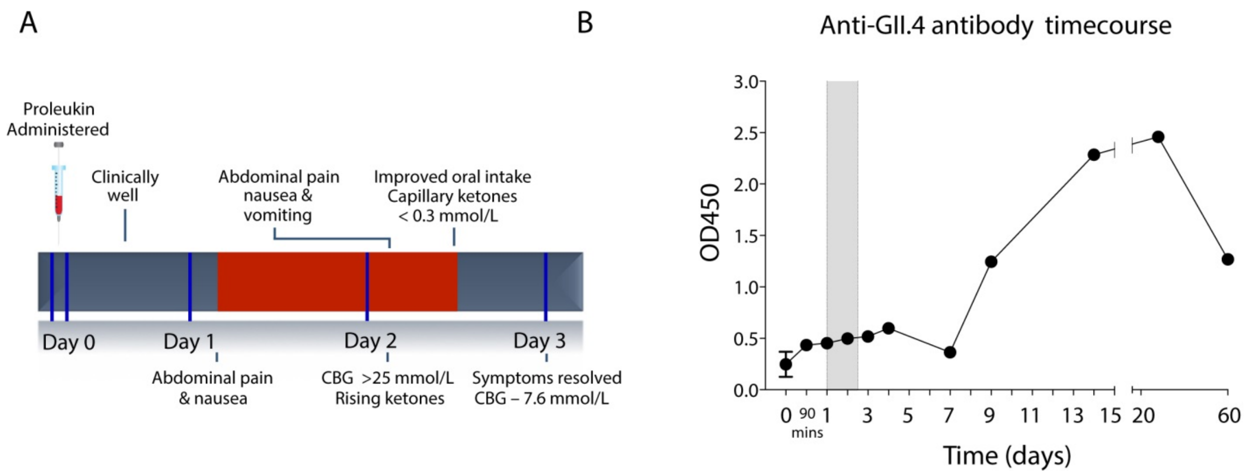

S2 Fig. Clinical course and anti-norovirus GII.4 antibody response of Norovirus infected participant. (**A**) Clinical course, development and conservative management of acute gastroenteritis. Blue lines indicate the blood sampling timepoints. Shaded red box indicates period of acute gastroenteritis symptoms. (**B**) Serum IgG titers against NoV GII.4 Dijon-strain virus-like particles (VLP) through the trial period in the participant with a gastrointestinal infection. The shaded area indicates the period of reported gastroenteritis.
