# Supplementary material for: Regulatory T Cell Responses in Participants with Type 1 Diabetes after a Single Dose of Interleukin-2: A Non-Randomised, Open Label, Adaptive Dose-Finding Trial
Source: PLoS Med. 2016 Oct 11;13(10):e1002139. doi: 10.1371/journal.pmed.1002139 (PMC5058548; doi:10.1371/journal.pmed.1002139)
Supplement: S3 Fig — (PDF) [file pmed.1002139.s016.pdf]

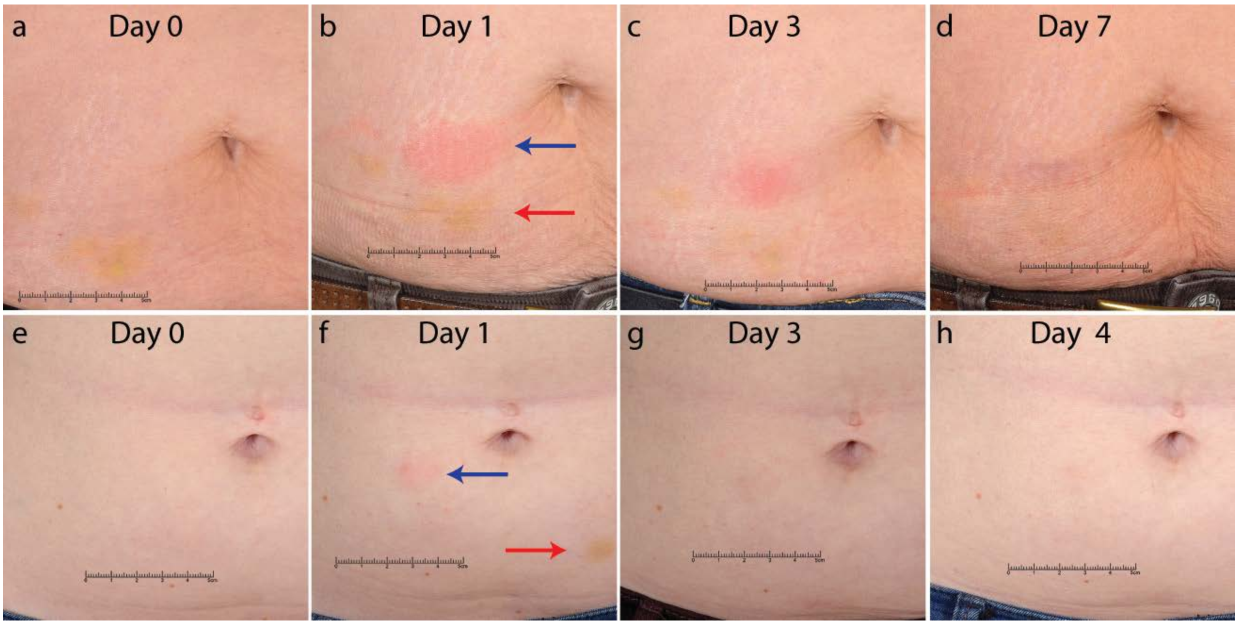

S3 Fig. Injection site reactions. Almost all participants had a delayed injection site reaction that was observed on day 1. Representative clinical photographs of the abdomens of two female participants showing typical injection site reactions through time of a participant administered 0.620 x 10^6 IU/m^2 of Proleukin (a-d) and 0.045 x 10^6 IU/m^2 (e-h). The site reaction consisted of non-itchy erythematous rash followed by the formation of a palpable subcutaneous nodule. The blue arrows mark the site reaction; red arrows mark the ecchymosis from subcutaneous insulin administration; ruler 0-5 cm.
