# Supplementary material for: Regulatory T Cell Responses in Participants with Type 1 Diabetes after a Single Dose of Interleukin-2: A Non-Randomised, Open Label, Adaptive Dose-Finding Trial
Source: PLoS Med. 2016 Oct 11;13(10):e1002139. doi: 10.1371/journal.pmed.1002139 (PMC5058548; doi:10.1371/journal.pmed.1002139)
Supplement: S4 Fig — (PDF) [file pmed.1002139.s017.pdf]

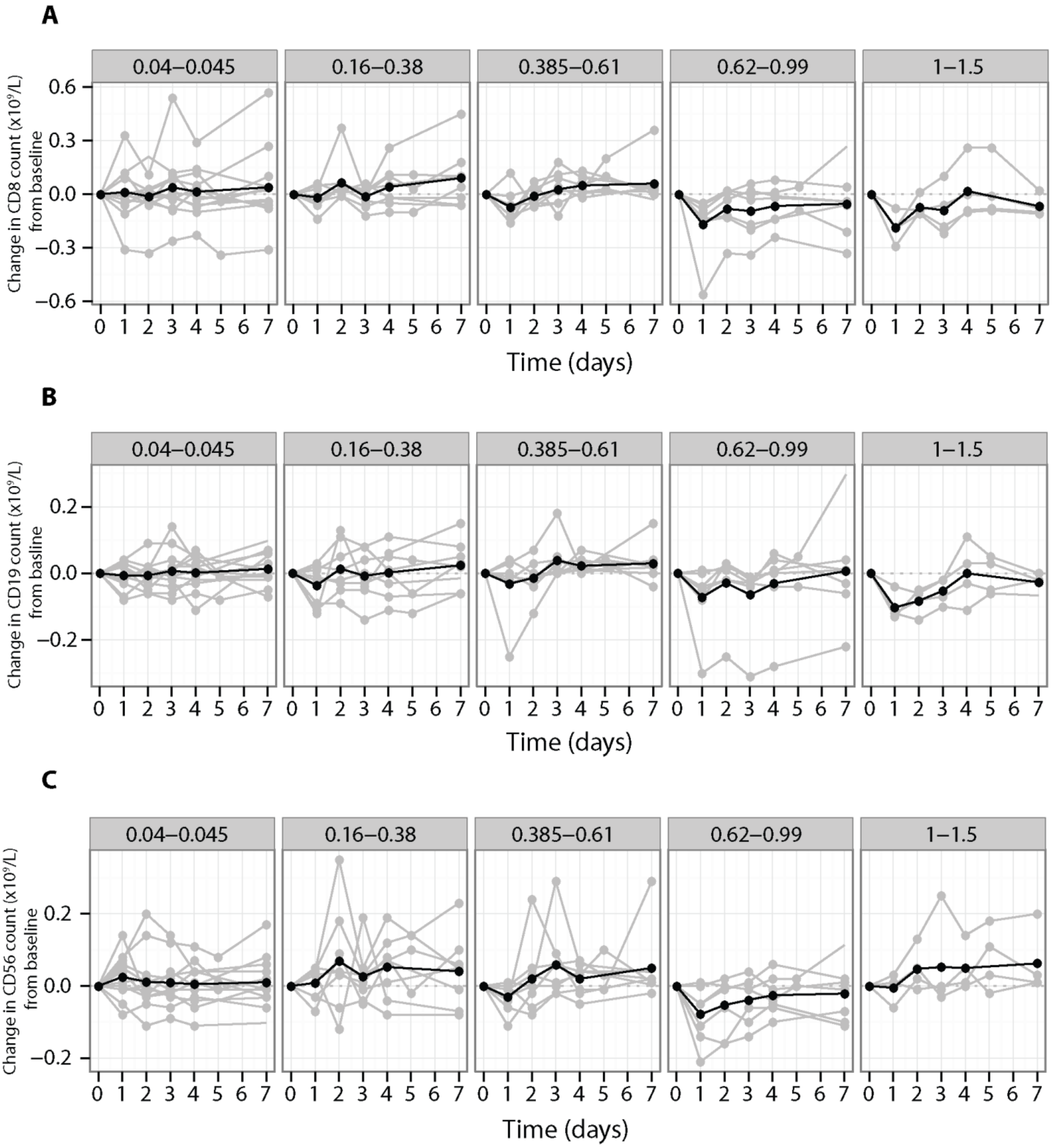

S4 Fig. Changes in CD8$^+$ T, B and NK cell counts in response to Proleukin. (**A**) CD8$^+$ T cells in blood have a dose-dependent decrease in number in response to treatment on day 0. (**B**) B cell counts also decrease recovering to pretreatment ranges by day 4. (**C**) Increase in total NK counts at the two highest doses
