# Supplementary material for: Regulatory T Cell Responses in Participants with Type 1 Diabetes after a Single Dose of Interleukin-2: A Non-Randomised, Open Label, Adaptive Dose-Finding Trial
Source: PLoS Med. 2016 Oct 11;13(10):e1002139. doi: 10.1371/journal.pmed.1002139 (PMC5058548; doi:10.1371/journal.pmed.1002139)
Supplement: S5 Fig — (PDF) [file pmed.1002139.s018.pdf]

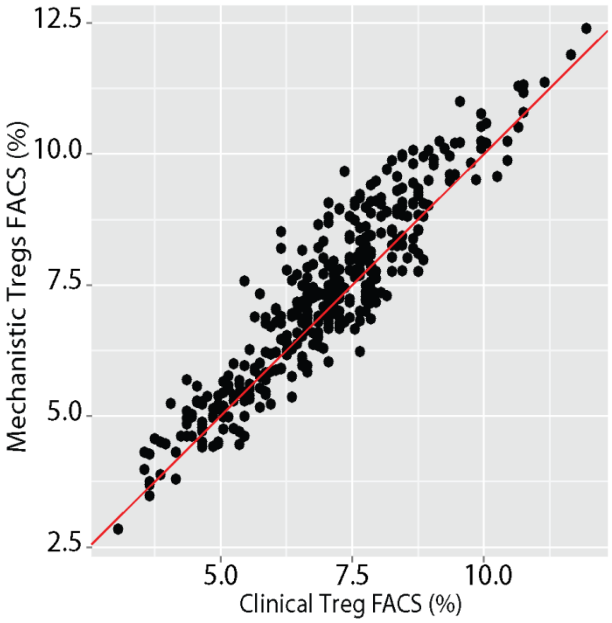

S5 Fig. Correlation between clinical and mechanistic FACS analysis of Tregs. The measurement of Tregs as a percentage of $CD4^+$ T cells between the two independent Treg FACS assays from days 1-7 (Pearson's correlation coefficient= 0.93; t-test $p < 2.2 \times 10^{-16}$; N=377).
