# Supplementary material for: Regulatory T Cell Responses in Participants with Type 1 Diabetes after a Single Dose of Interleukin-2: A Non-Randomised, Open Label, Adaptive Dose-Finding Trial
Source: PLoS Med. 2016 Oct 11;13(10):e1002139. doi: 10.1371/journal.pmed.1002139 (PMC5058548; doi:10.1371/journal.pmed.1002139)
Supplement: S6 Fig — (PDF) [file pmed.1002139.s019.pdf]

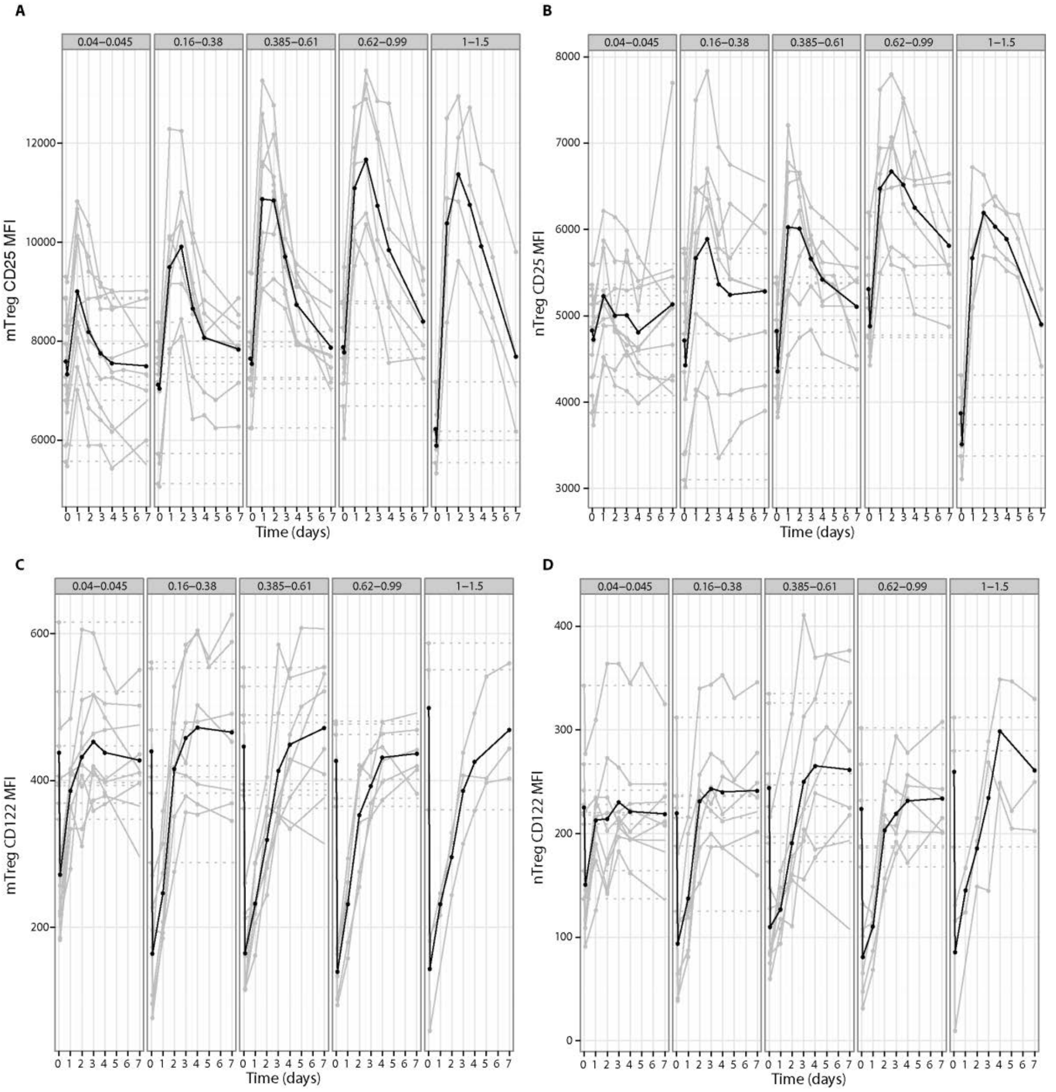

S6 Fig. CD25 and CD122 expression on memory and naïve Tregs from baseline to day 7 post-treatment. (**A and B**) CD25 expression is higher on mTregs than nTregs and both subsets have a longer duration of response at higher doses (baseline nTreg CD25 MFI average = 4792 stardard error = 132; range 3103-6120 Number of participants =37). (**C and D**) CD122 expression is higher in mTreg than nTreg and declines in a dose-dependent manner on both subsets with a longer durationof decline at higher doses (baseline nTreg CD122 MFI (231 (9.73) 125-343 N=33). [Black lines show the mean values for each dose group while the grey lines show the individual participants, mean fluorescence intensity (MFI)]
