# Supplementary material for: Regulatory T Cell Responses in Participants with Type 1 Diabetes after a Single Dose of Interleukin-2: A Non-Randomised, Open Label, Adaptive Dose-Finding Trial
Source: PLoS Med. 2016 Oct 11;13(10):e1002139. doi: 10.1371/journal.pmed.1002139 (PMC5058548; doi:10.1371/journal.pmed.1002139)
Supplement: S7 Fig — (PDF) [file pmed.1002139.s020.pdf]

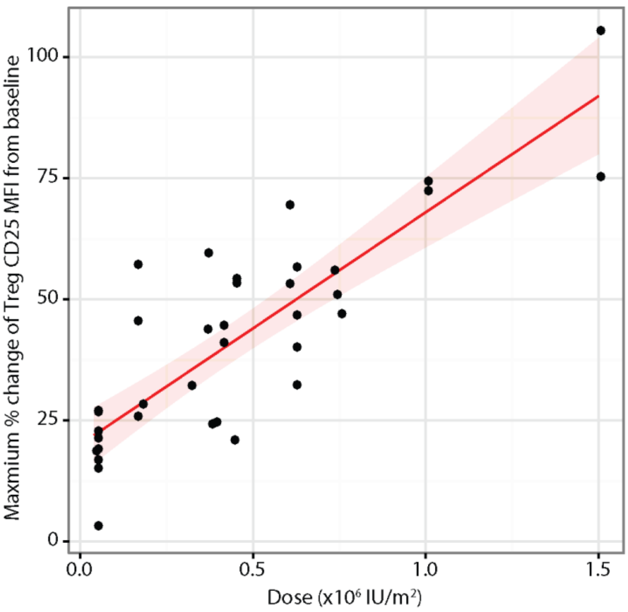

S7 Fig. Linear increase in CD25 expression on Tregs in response to increased Proleukin dose. A linear model best describes the Treg CD25 dose response to Proleukin (Day 0-7) (mean baseline measurement of Treg CD25 MFI=6526 (SE=167; range 4576-8270) N=37). [The shaded area presents the 95% confidence interval of the fitted linear model.]
