# Supplementary material for: Regulatory T Cell Responses in Participants with Type 1 Diabetes after a Single Dose of Interleukin-2: A Non-Randomised, Open Label, Adaptive Dose-Finding Trial
Source: PLoS Med. 2016 Oct 11;13(10):e1002139. doi: 10.1371/journal.pmed.1002139 (PMC5058548; doi:10.1371/journal.pmed.1002139)
Supplement: S8 Fig — (PDF) [file pmed.1002139.s021.pdf]

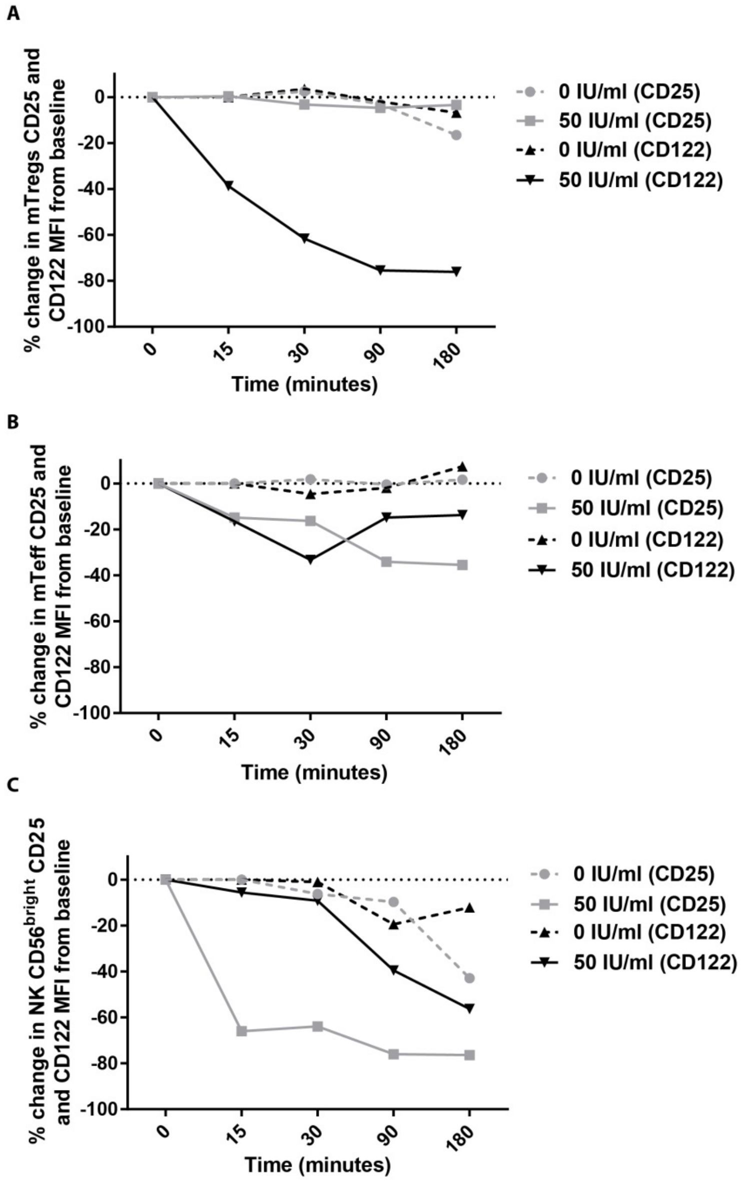

S8 Fig. Effects *in vitro* of Proleukin on CD25 and CD122 expression on NK CD56^{bright}, memory T regulatory, and T effector cells. **(A)** mTregs demonstrate a rapid decrease in CD122 MFI from baseline following Proleukin, plateauing at 90 minutes (baseline CD25 MFI = 5429, maximum % change from baseline = -4.8) (baseline CD122 MFI = 1263, maximum % change from baseline = -76.1) . **(B)** Both CD25 and CD122 MFI have a decrease on mTeff (CD25 MFI = 855, maximum % change from baseline = -35.6%) (CD122 MFI 610, maximum % change from baseline = -33.3 . **(C)** NK CD56^{brights} show a rapid fall in CD25 MFI, with a less pronounced reduction in CD122 MFI after 90 minutes (CD25 MFI = 380, maximum % change from baseline = -76.4) (CD122 MFI = 4520, maximum % change from baseline = -56.3).
