# Supplementary material for: Regulatory T Cell Responses in Participants with Type 1 Diabetes after a Single Dose of Interleukin-2: A Non-Randomised, Open Label, Adaptive Dose-Finding Trial
Source: PLoS Med. 2016 Oct 11;13(10):e1002139. doi: 10.1371/journal.pmed.1002139 (PMC5058548; doi:10.1371/journal.pmed.1002139)
Supplement: S9 Fig — (PDF) [file pmed.1002139.s022.pdf]

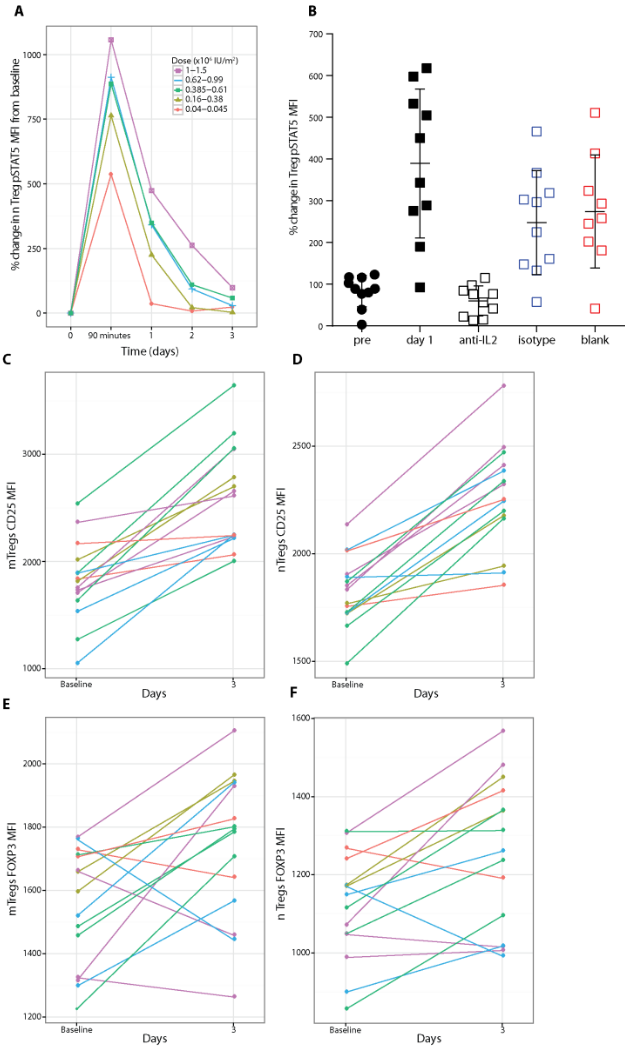

S9 Fig. Increased pSTAT5, CD25 and FOXP3 levels in T regulatory cell subsets in blood following a dose of Proleukin. (**A**) The maximal pSTAT5 response of nTregs to treatment was at 90 minutes and was sustained for several days at the higher doses (nTreg average baseline MFI 8.71 (SE=0.32; Range=5-15) N=36) (**B**) The increased level of pSTAT5 on day 1 was eliminated by incubating the blood for 2 hours with a neutralizing anti-IL-2 antibody prior to initiating the staining procedure. Increase in expression of CD25 on (**C**) mTregs and (**D**) nTregs on day 3 post-treatment using cryopreserved PBMC. Increase in FOXP3 expression 3 days following treatment on (**E**) mTregs and (**F**) nTregs using cryopreserved PBMC.
