# Supplementary material for: Regulatory T Cell Responses in Participants with Type 1 Diabetes after a Single Dose of Interleukin-2: A Non-Randomised, Open Label, Adaptive Dose-Finding Trial
Source: PLoS Med. 2016 Oct 11;13(10):e1002139. doi: 10.1371/journal.pmed.1002139 (PMC5058548; doi:10.1371/journal.pmed.1002139)
Supplement: S10 Fig — (PDF) [file pmed.1002139.s023.pdf]

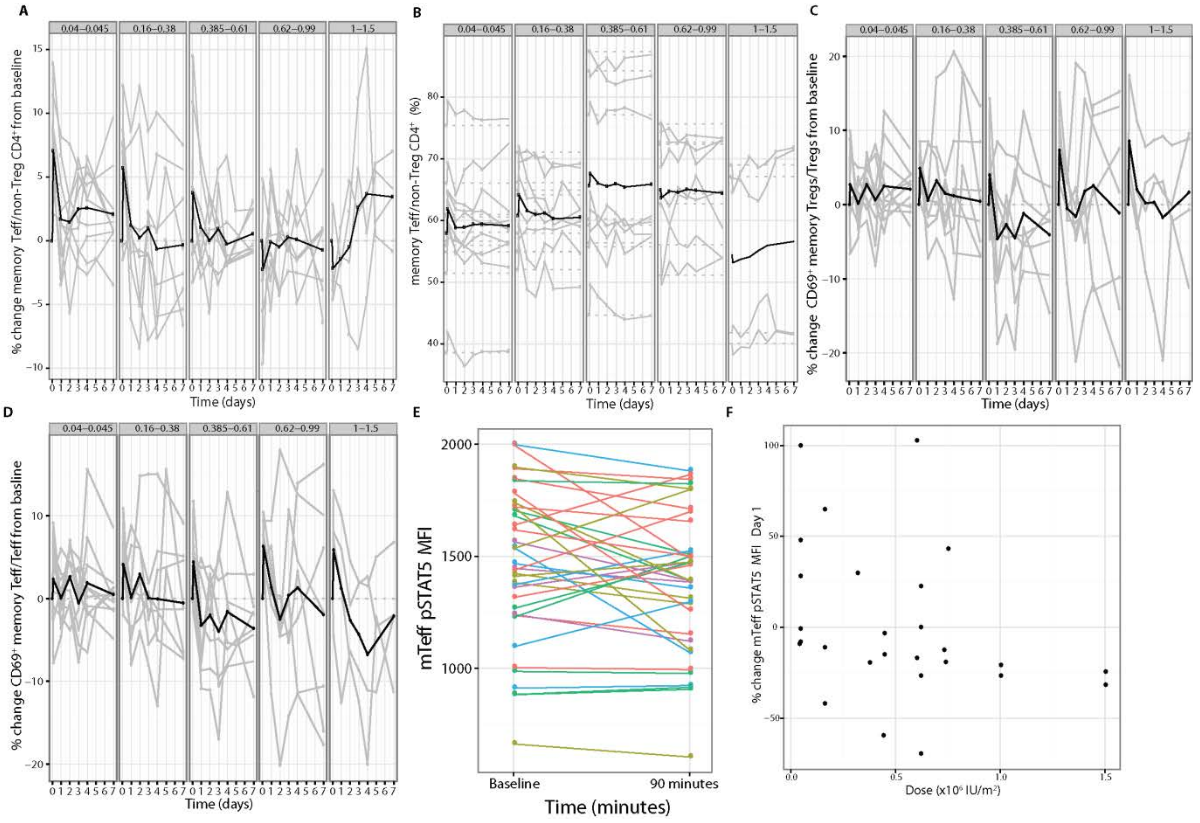

S10 Fig. The effects of a Proleukin dose on memory T effector cell frequency and CD69$^+$ T regulatory and effector cells and pSTAT5 response in mTeff cells. (**A**) Dose response by memory T effectors (mTeffs) (% non-Treg CD4$^+$ T cells) following treatment with lower doses leading to an early increase in mTeff at 90 minutes while highest doses lead to a decrease followed by a sustained increase in blood. (**B**) Absolute frequency of mTeff (% non-Treg CD4$^+$ T cells). (**C**) There was a small increase in CD69$^+$ mTregs (% Tregs) at 90 minutes (average baseline CD69$^+$ mTregs 21.84 % (SE=1.06; range= 6.04-34.4), N=37) (**D**) with a similar response in CD69$^+$ mTeffs (% Teffs) in blood at 90 minutes without dose dependency (average baseline CD69$^+$ mTeff 23.94 % (0.98; 11.7-36.9) N= 37). (**E**) Compared to baseline there was reduction in the pSTAT5 response at 90 minutes in mTeffs after maximum *in vitro* IL-2 stimulation (1000 IU/ml). (**F**) Using cryopreserved PBMC, on Day 1 there is no evidence of a dose-dependent reduction in response (pSTAT5) when mTeff were stimulated *in vitro* with Proleukin (10 IU/ml). [Black lines show the mean values for each dose group while the grey lines show the individual participant's values]
