# Supplementary material for: Regulatory T Cell Responses in Participants with Type 1 Diabetes after a Single Dose of Interleukin-2: A Non-Randomised, Open Label, Adaptive Dose-Finding Trial
Source: PLoS Med. 2016 Oct 11;13(10):e1002139. doi: 10.1371/journal.pmed.1002139 (PMC5058548; doi:10.1371/journal.pmed.1002139)
Supplement: S11 Fig — (PDF) [file pmed.1002139.s024.pdf]

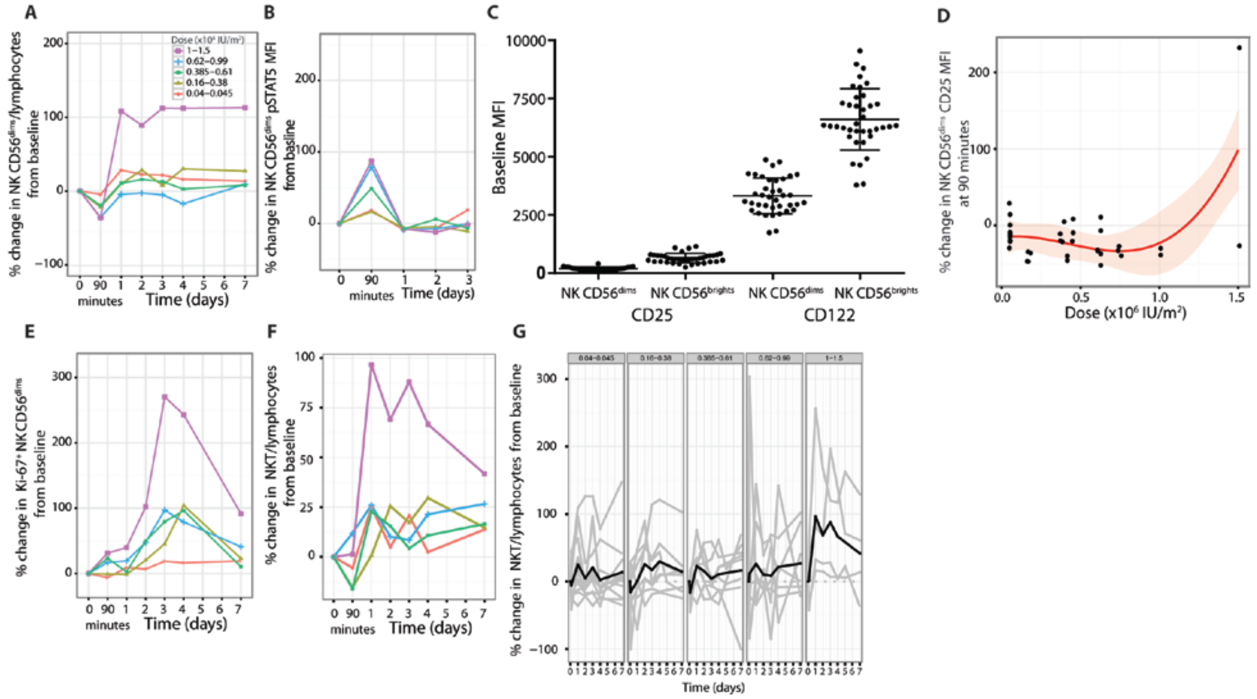

S11 Fig. Natural killer cell responses to treatment and baseline expression of CD25 and CD122. (A) NK CD56^{dims} have a large and sustained increase as a % of lymphocytes at the highest doses of 1.0-1.5 x 10^6 IU/m^2 (average baseline NK CD56^{dims} (% lymphocytes) 8.09 % SE= 0.61 range= 2.09-18.9, N=38). **(B)** NK CD56^{dims} pSTAT5 response at 90 minutes (average baseline pSTAT5 MFI 20.46 (1.2) 0-40.62, N=34). (**C)** Pre-treatment expression of CD25 and CD122 on NK CD56^{dims} and NK CD56^{brights}. (**D)** CD25 expression on NK CD56^{dims} at 90 minutes following treatment (baseline NK CD56^{dims} CD25 MFI 184 (11) 83.8-401, N=38). (**E**) Change in frequency of Ki-67$^+$ NK CD56^{dims} with the greatest increase at the highest doses (1.0-1.5 x 10^6 IU/m^2) (baseline Ki-67$^+$ NK CD56^{dims} 4.49 % (0.52) 1.5-13.1, N=30). (**F**) NKT response to Proleukin at 90 minutes and on following days post treatment (average baseline NKT (% lymphocytes) 2.18% SE=0.26 range=0.06-7.03, N=38). **(E)** Individual plots of NKT response in participants (grey lines) with black lines showing the mean values for each dose group
