# Supplementary material for: Regulatory T Cell Responses in Participants with Type 1 Diabetes after a Single Dose of Interleukin-2: A Non-Randomised, Open Label, Adaptive Dose-Finding Trial
Source: PLoS Med. 2016 Oct 11;13(10):e1002139. doi: 10.1371/journal.pmed.1002139 (PMC5058548; doi:10.1371/journal.pmed.1002139)
Supplement: S12 Fig — (PDF) [file pmed.1002139.s025.pdf]

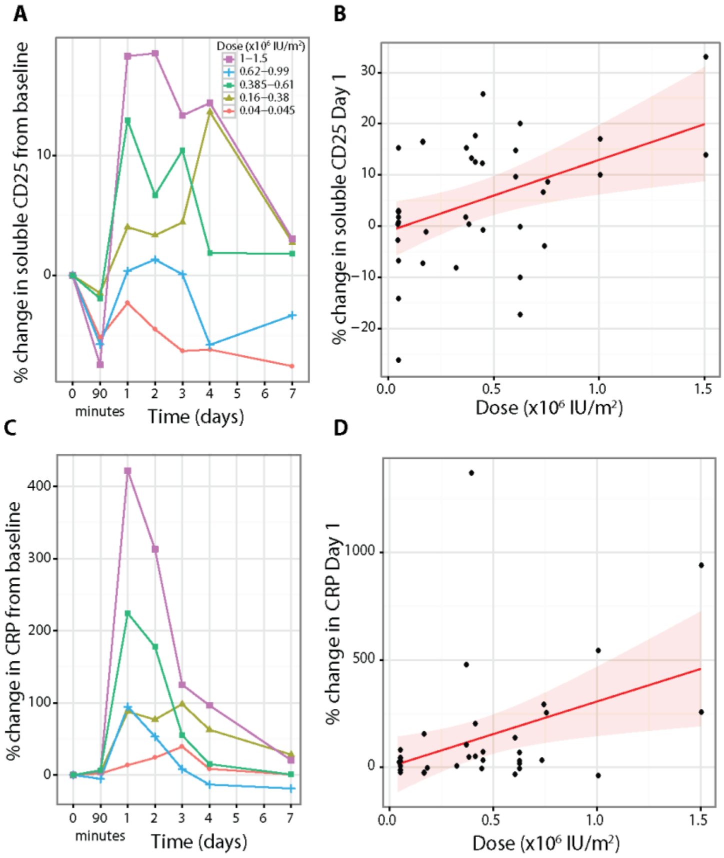

S12 Fig. Soluble CD25 and C-reactive protein responses. (**A and B)** Soluble CD25 increased linearly with dose on day 1 (average baseline 2.5 x 10^3 pg/ml (0.09; 1.55 – 4.07); N=38). **(C and D)** Acute phase protein C-reactive protein (CRP) showed a linear dose response on day 1 (average baseline 2.98 x 10^6 pg/ml (0.78; 0.12-23.25); N=37). **[**The shared area presents the 95% confidence interval of the fitted models]
