# Supplementary material for: Regulatory T Cell Responses in Participants with Type 1 Diabetes after a Single Dose of Interleukin-2: A Non-Randomised, Open Label, Adaptive Dose-Finding Trial
Source: PLoS Med. 2016 Oct 11;13(10):e1002139. doi: 10.1371/journal.pmed.1002139 (PMC5058548; doi:10.1371/journal.pmed.1002139)
Supplement: S13 Fig — (PDF) [file pmed.1002139.s026.pdf]

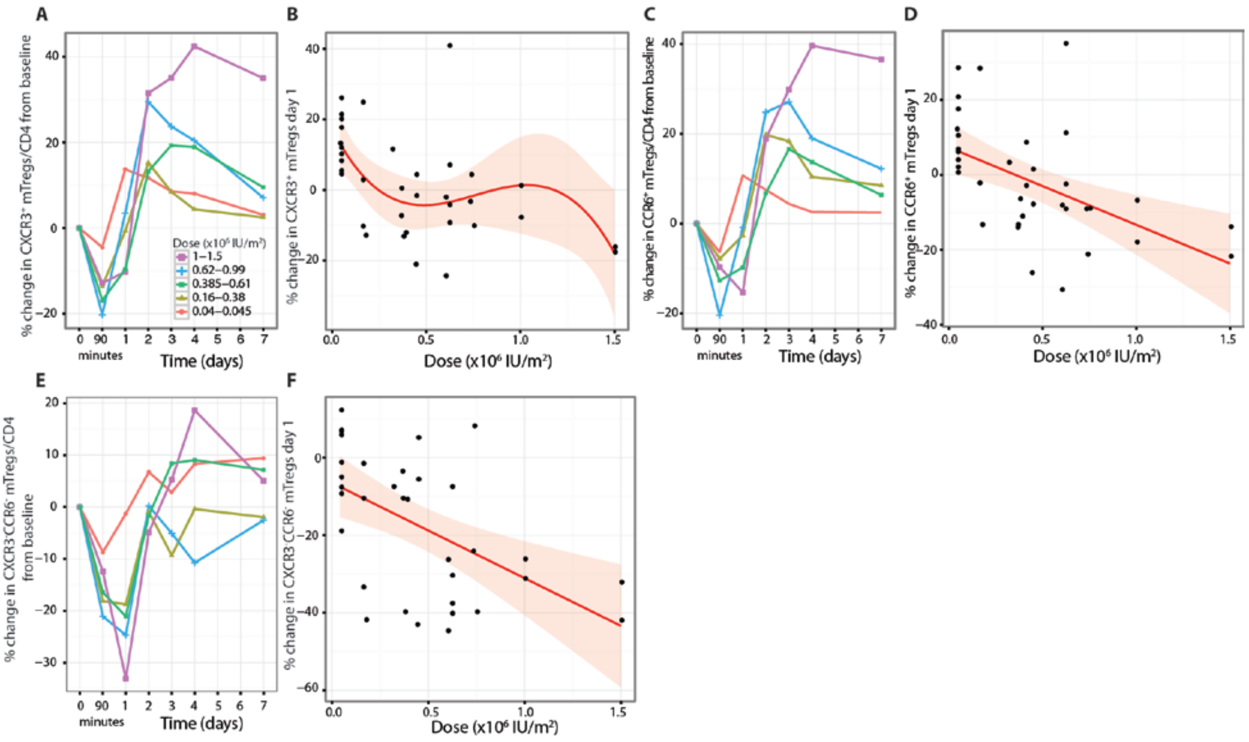

S13 Fig. Changes in frequency of CXCR3$^+$,CCR6$^+$ and CXCR3$^-$CCR6$^-$memory Tregs. (A and B) Rapid dose-dependent reduction in the frequency of CXCR3$^+$ mTregs followed by an increase (average baseline mTregs (% CD4 T cells) 2.04% (0.12; 0.94-3.69) N = 35) induced by Proleukin. (**C**) A similar reduction in the percentage of CCR6$^+$ mTregs was seen after a dose of Proleukin followed by a sustained increase over several days. (**D**) The decrease in CCR6$^+$ mTregs was dose dependent with a linear response on day 1 (average baseline CCR6$^+$ mTreg (out of % CD4 T cells) 3.0% (0.14; 1.7 - 5.84) N=37). (**E and F)** CXCR3$^-$CCR6$^-$ mTregs also had a dose-dependent decline in frequency in blood following treatment with Proleukin (average baseline 0.82% (0.05; 0.36-1.54) N = 34). **[**The shared area presents the 95% confidence interval of the fitted models]
