# Supplementary material for: Regulatory T Cell Responses in Participants with Type 1 Diabetes after a Single Dose of Interleukin-2: A Non-Randomised, Open Label, Adaptive Dose-Finding Trial
Source: PLoS Med. 2016 Oct 11;13(10):e1002139. doi: 10.1371/journal.pmed.1002139 (PMC5058548; doi:10.1371/journal.pmed.1002139)
Supplement: S14 Fig — (PDF) [file pmed.1002139.s027.pdf]

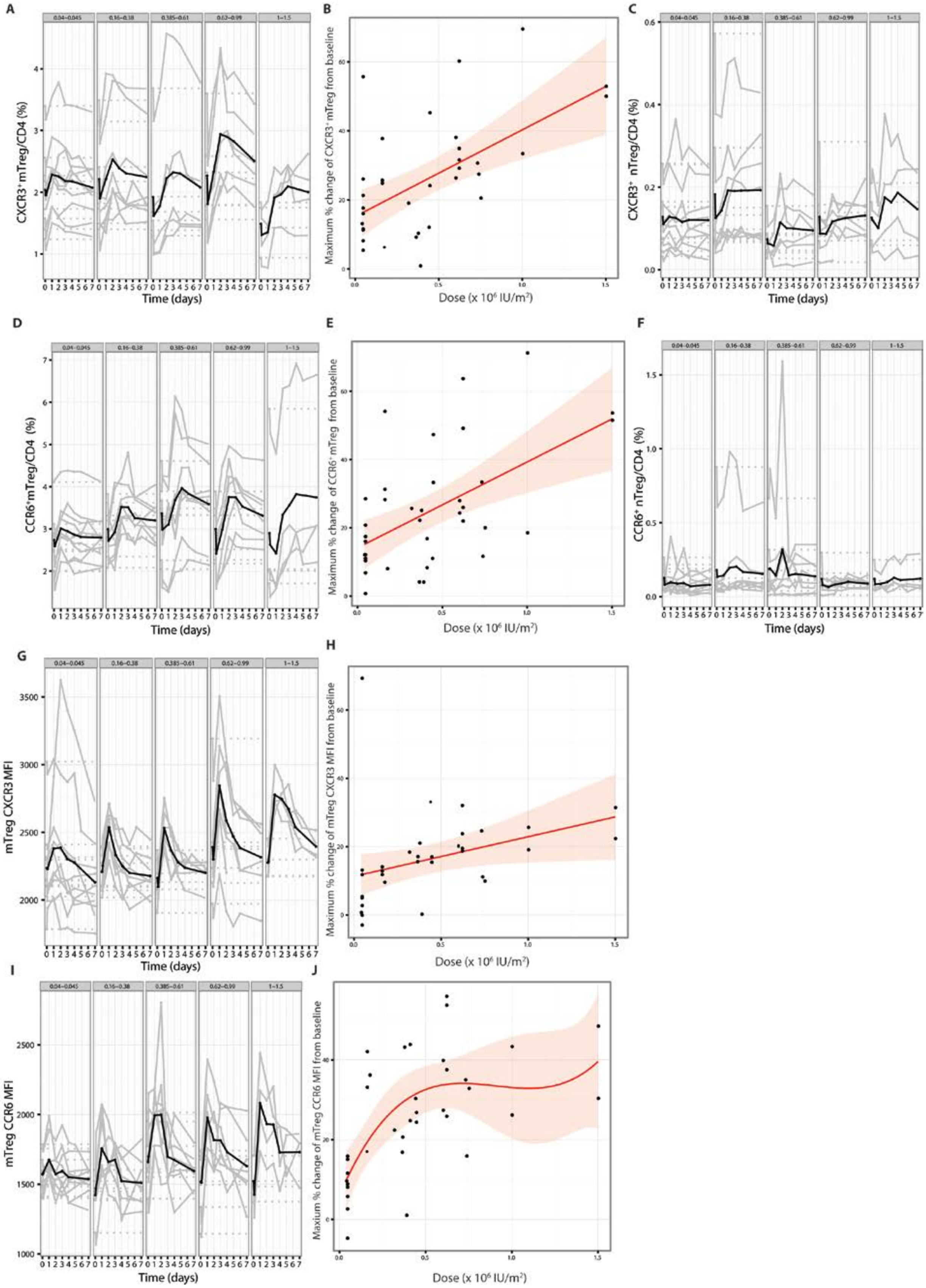
A
B
C
D
E
F
G
H
I
J
0.04–0.045
0.16–0.38
0.385–0.61
0.62–0.99
1–1.5
CXCR3⁺ mTreg/CD4 (%)
CXCR3⁺ nTreg/CD4 (%)
CCR6⁺mTreg/CD4 (%)
CCR6⁺ nTreg/CD4 (%)
mTreg CXCR3 MFI
mTreg CCR6 MFI
Time (days)
Maximum % change of CXCR3⁺ mTreg from baseline
Maximum % change of CCR6⁺ mTreg from baseline
Maximum % change of mTreg CXCR3 MFI from baseline
Maxium % change of mTreg CCR6 MFI from baseline
Dose (x 10⁶ IU/m²)

S14 Fig. CXCR3$^+$ and CCR6$^+$ memory and naive T regulatory cell responses to treatment. (**A**) Absolute frequency of CXCR3$^+$ mTreg (% CD4 T cells) across the five dose groups. (**B**) A linear model describes the dose response best with the maximum percentage increase from baseline of CXCR3$^+$ mTreg in each participant over the 7 days following treatment shown (average baseline CXCR3$^+$ mTreg 2.03 % SE= 0.11 range = 0.94-3.68, N=35). (**C**) Pre-treatment absolute frequencies at day 0 and response of CXCR3$^+$ nTreg (% CD4 T cells) following a dose of Proleukin (baseline 0.13 % (0.01) 0.01-0.5, N=35). (**D**) Absolute frequencies of CCR6$^+$ mTregs (% CD4$^+$ T cells). (**F**) Dose response of CCR6$^+$ mTreg to Proleukin over the 7 days following treatment (baseline CCR6$^+$ mTreg 2.99 % (0.14) 1.7-5.84, N=37). (**E**) Absolute CCR6$^+$ nTreg (% CD4 T cells) frequencies following treatment (baseline 0.14 % (0.02) 0-0.87, N=37). (**G**) Increased CXCR3 mean fluorescence intensity (MFI) on mTreg following treatment. (**H**) Dose-dependent increase in CXCR3 expression on mTregs over the 7 days following treatment (baseline 2252 MFI (45.08) 1783-3193, N=35). (**I**) Increased expression of CCR6 on mTregs (baseline average 1542 MFI (30.58) 1114-2014). (**J**) A cubic dose-response model describes the increase in expression of CCR6 on mTregs best in the 7 days following treatment. [Black lines show the mean values for each dose group while the grey lines show the individual participants. The shaded area presents the 95% confidence interval of the fitted model]
