# Supplementary material for: Regulatory T Cell Responses in Participants with Type 1 Diabetes after a Single Dose of Interleukin-2: A Non-Randomised, Open Label, Adaptive Dose-Finding Trial
Source: PLoS Med. 2016 Oct 11;13(10):e1002139. doi: 10.1371/journal.pmed.1002139 (PMC5058548; doi:10.1371/journal.pmed.1002139)
Supplement: S1 Materials — (PDF) [file pmed.1002139.s028.pdf]

S1 Materials

DILT1D: Standard operating procedure Flow Cytometry Staining and Cell Sorting

A. Reagents

1. **Staining reagents**
   - **FACS Lysing Solution** (BD: 349202)

   Diluted 5 ml of 10X stock solution with 45 ml room temperature Ambion nuclease-free water (ABI: AM9932 1 litre).
   - **PBS (pH7.2) + BSA (7.5%)** (Gibco: 20012019 + Sigma: A8412)
   - **Brilliant Stain Buffer** (BD: 563794)

2. **Sorting reagents**
   - **RBC Lysis Buffer** (eBioscience: 00433357)
   - **XVIVO + 1% Human AB Sera** (Lonza: BE04418F + Sigma: H4522)

B. Preparation of whole blood samples for surface staining

1. Aliquot optimized volumes of antibodies into pre-labeled FACS tubes containing 50 µl Brilliant Stain Buffer (tubes 1-6, Table S6 & S7).
2. Add 150 µl of whole blood to each tube and vortex.
3. Cover samples with foil and incubate for 45 minutes at room temperature.
5. Add 2 ml of BD FACS Lysing Solution (as prepared in Part A) to each tube and vortex.
6. Incubate for 8 minutes at room temperature.
7. Centrifuge for 8 minutes at 700g at room temperature.
8. Pour off supernatant and rack tubes gently.
9. Add 2 ml PBS + BSA to each tube and centrifuge again for 8 minutes at 700g at 4°C.
11. Pour off supernatant and rack tubes gently.
12. Resuspend in 200 µl of PBS + BSA.
13. Place tubes on ice and cover with foil. Samples are now ready to be analyzed on the BD Fortessa cytometer.

C. Preparation of whole blood samples intra-cellular staining

1. Aliquot optimized volumes of surface antibodies into a FACS tube containing 50 µl Brilliant Stain Buffer (tube 7, Table S6 & S7).
2. Add 200 µl of whole blood to each tube and vortex.
3. Cover samples with foil and incubate for 45 minutes at room temperature.
4. Add 500 µl of Fixation/Permeabilization (as prepared in Part A) solution to each tube and vortex.
5. Cover samples with foil and incubate for 30 minutes at room temperature.
6. Add 3 ml of Permeabilization Buffer (as prepared in Part A) to each tube and vortex.

7. Place on ice, cover with foil and incubate for 5 minutes, then centrifuge for 5 minutes at 700g at 4°C.
8. Pour off supernatant and rack tubes gently.
9. Perform an additional wash by repeating steps 8 to 10.
10. Add pre-optimized volumes of intracellular antibodies (shaded cells in tube 7, Table **) and vortex. Cover with foil and incubate for 45 minutes at 4°C.
11. Perform two more washes by repeating steps 8 to 10 twice.
12. Resuspend in 200 µl of PBS + BSA.
13. Place tubes on ice and cover with foil. Analyze samples on the BD Fortessa cytometer.

D. Preparation of whole blood samples for cell sorting

1. Label one 50 ml falcon tube and one FACS tube per sample with appropriate sample ID.
2. Aliquot sorting antibodies to the appropriate tube (Table S7 & S8).
3. Add 3 ml of whole blood to each tube and vortex.
4. Cover samples with foil and incubate for 1 hour at room temperature, vortexing again after 30 minutes.
5. Add 30 ml of 1x RBC Lysis buffer (eBioscience) to each sample and vortex.
6. Cover with foil and incubate at room temperature for 10 minutes, vortexing again every few minutes.
7. Top the sample to 50 ml with room temperature PBS. Centrifuge for 8 minutes at 700g at 4°C.
8. Pour off supernatant and resuspend in 500 µl XVIVO + 1% Human AB Sera and transfer to a labelled FACS tube. Place tubes on ice and cover with foil.
9. Label up four 1.5 ml DNA LoBind safe-lock eppendorfs with the following ASA barcode labels:
   - TTreg_HEP (total Treg)
   - MemTeff_HEP (memory central)
   - 62L-MTeff_HEP (memory effector)
   - CD56NK_HEP (CD56 bright)

11 Add 500 µl XVIVO + 1% AB to each tube.

12 Analyze samples on the cell sorter (BD AriaIII/AriaFusion
