# Supplementary material for: Regulatory T Cell Responses in Participants with Type 1 Diabetes after a Single Dose of Interleukin-2: A Non-Randomised, Open Label, Adaptive Dose-Finding Trial
Source: PLoS Med. 2016 Oct 11;13(10):e1002139. doi: 10.1371/journal.pmed.1002139 (PMC5058548; doi:10.1371/journal.pmed.1002139)
Supplement: S2 Materials — (PDF) [file pmed.1002139.s029.pdf]

Mechanistic flow quality control (QC) process

While all but the primary endpoint in clinical analyses reports using the Evaluable population (N=39), the numbers reported in mechanistic analyses varies. In line with the CONSORT checklist items 16 (Numbers analysed) and 18 (Ancillary analyses), and as an aid to reproducibility, we have recorded the reasons why participants and visits do not feature in mechanistic analyses in a **mechanistic QC failure log (*QClog*)**:

- Some samples failed QC
- Some analyses were only attempted on samples from a subset of participants and visits

The mechanistic flow QC process is described schematically on the next page:

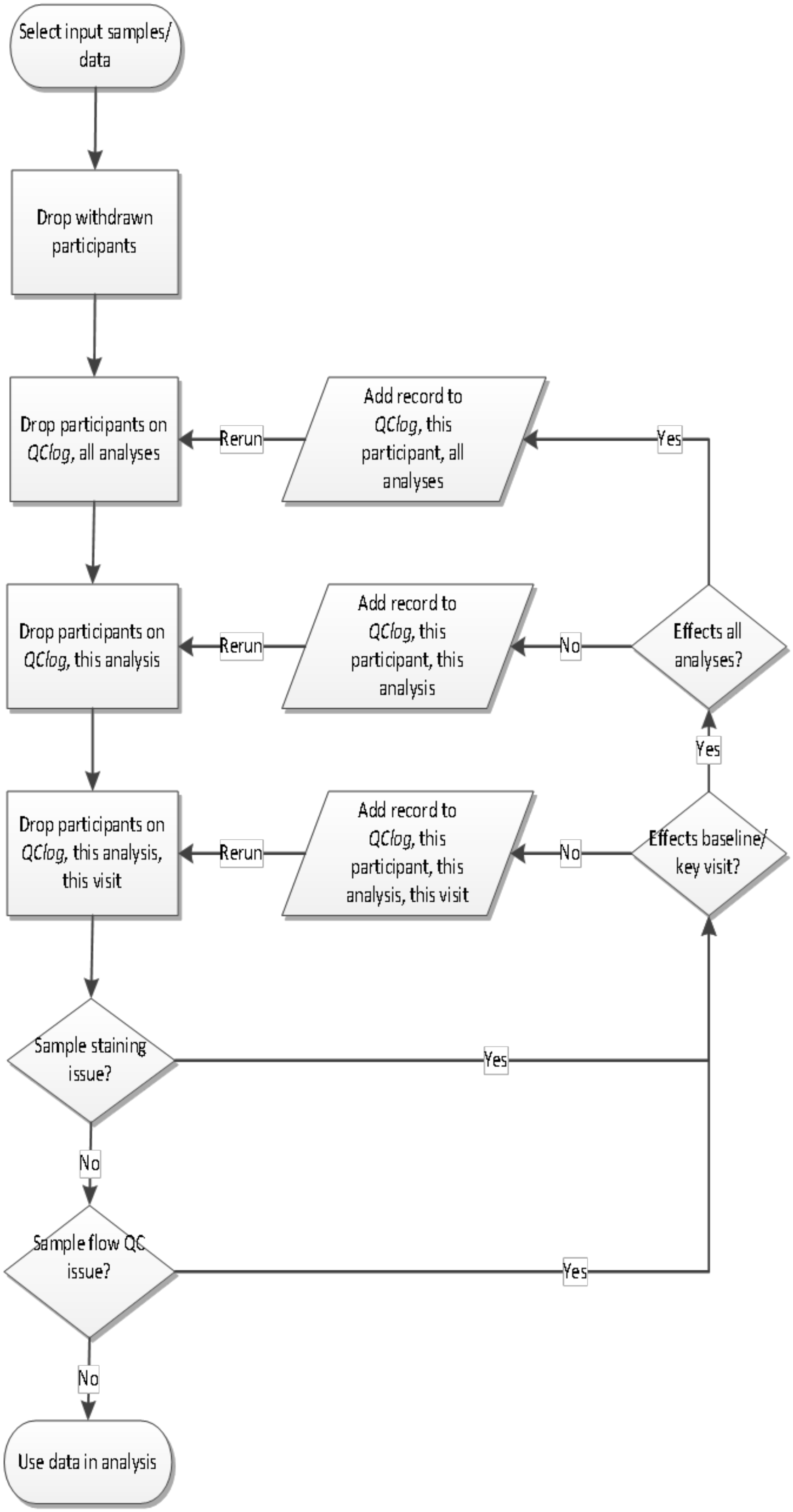
Select input samples/ data
Drop withdrawn participants
Drop participants on QClog, all analyses
Rerun
Add record to QClog, this participant, all analyses
Yes
Drop participants on QClog, this analysis
Rerun
Add record to QClog, this participant, this analysis
No
Effects all analyses?
Yes
Drop participants on QClog, this analysis, this visit
Rerun
Add record to QClog, this participant, this analysis, this visit
No
Effects baseline/ key visit?
Sample staining issue?
Yes
No
Sample flow QC issue?
Yes
No
Use data in analysis

The overall method is that – having been selected – samples and data from participants at various visits can be dropped from all analyses, one analysis or just one visit on the programmatic application of entries in the *QClog* to the most complete starting input dataset. This programmatic application allows for the inclusion or exclusion of the withdrawn participant in preparing datasets for both this paper and the accompanying paper on immune response to norovirus.

Missing mechanistic data summary

There were 41 mechanistic analyses attempted based on flow data, with the following records dropped:

1. 1 participant was withdrawn – see CONSORT diagram, Fig 1A (main paper)
2. 1 participant was dropped from all secondary and exploratory analyses, due to recurrent Adverse Events
3. 51 participants were dropped from a whole analysis, on the basis of: missing baseline (37), missing intermediate visits (14)
4. 61 participants were dropped from a specific visit for an analysis, on the basis of: staining issue (45), flow QC issue (16).

As a worked example, looking at Fig 4C, we start with the Analysis Population (N=40), the set of treated participants:

1. 1 participant was withdrawn
2. 1 participant was dropped from all secondary and exploratory analyses
3. 1 participant was dropped on the basis of missing baseline
4. 0 participants were dropped from any specific visit.

This gives N=37.
