# Supplementary material for: Regulatory T Cell Responses in Participants with Type 1 Diabetes after a Single Dose of Interleukin-2: A Non-Randomised, Open Label, Adaptive Dose-Finding Trial
Source: PLoS Med. 2016 Oct 11;13(10):e1002139. doi: 10.1371/journal.pmed.1002139 (PMC5058548; doi:10.1371/journal.pmed.1002139)
Supplement: S3 Materials — (PDF) [file pmed.1002139.s030.pdf]

Interim Report DILT1D Trial

Simon Bond

January 29, 2014

1 Individual Patient Profiles

The patient profiles are shown overlaid in figure 1, and split up by dose in figure 2.

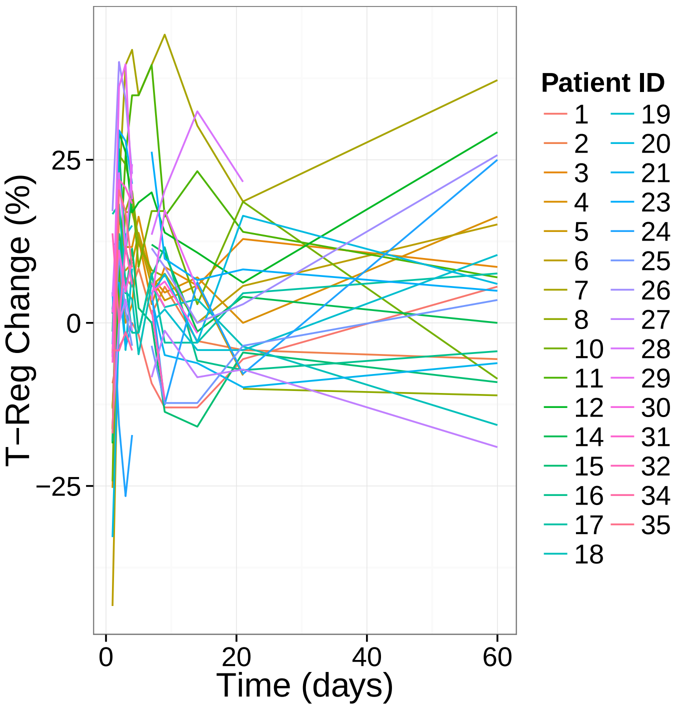

Figure 1: Spaghetti plot of all profiles

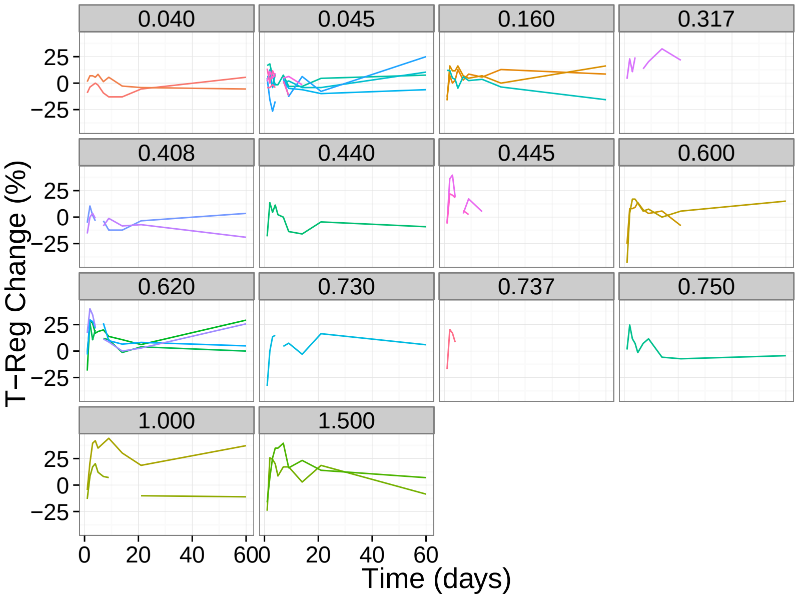

Figure 2: Profiles separated by dose ($\times 10^6 IU/m^2$)

2 Summary of dose and maximum Treg values

We have a plot of sequence of doses given in order in figure 3. A scatter plot of dose versus maximum T-reg change is given in figure 4

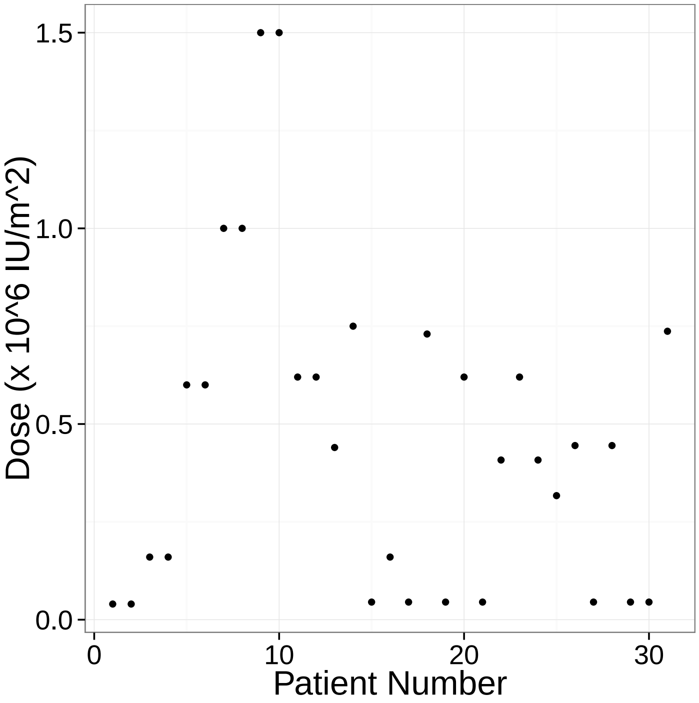

Figure 3: Dose Sequence

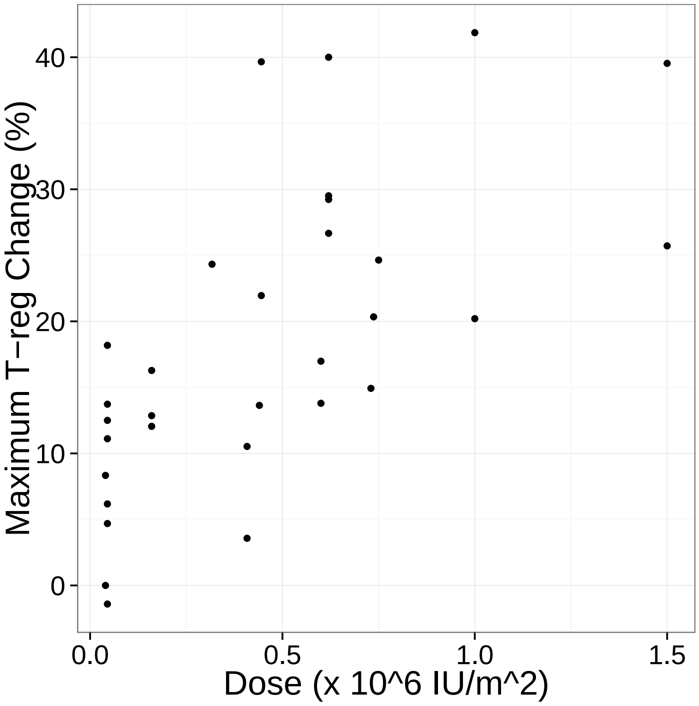

Figure 4: Scatter Plot of Dose versus Maximum T-reg change

3 Model Fitting and Dose Choice

The targets are set as 10% and 20%. Fitted models are estimated under a variety of different modelling assumptions

The following models did not converge: logistic, Emax4.

Figure 5 shows all the fitted models' predicted values overlaid onto the scatter plot of the raw data. The 95% confidence bands for the individual models are shown in figure 6.

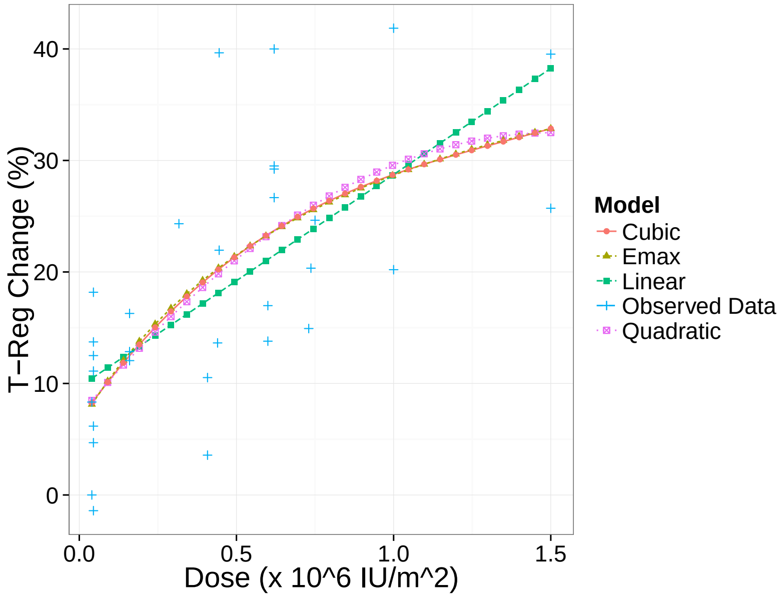

Figure 5: Fitted Values for the convergent Models

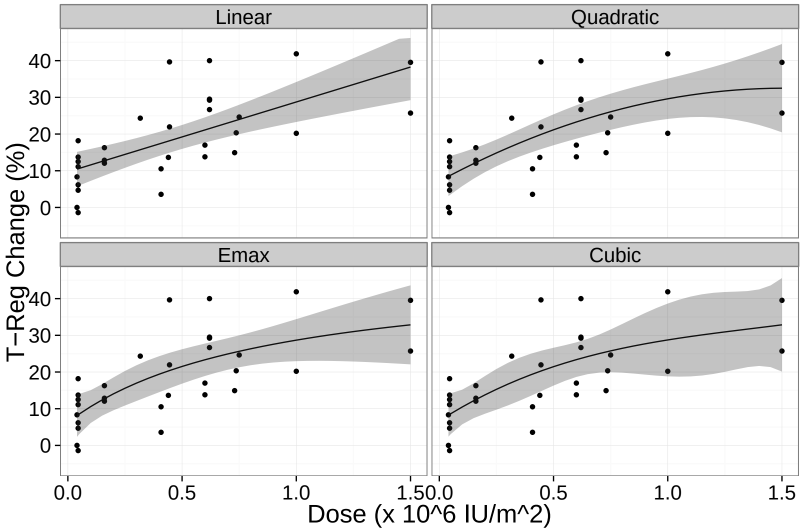

Figure 6: Model predictions with 95% pointwise confidence intervals

Figure 6 shows the uncertainty in the models about the population average *expected* value. It does not incoporate between-patient variability.

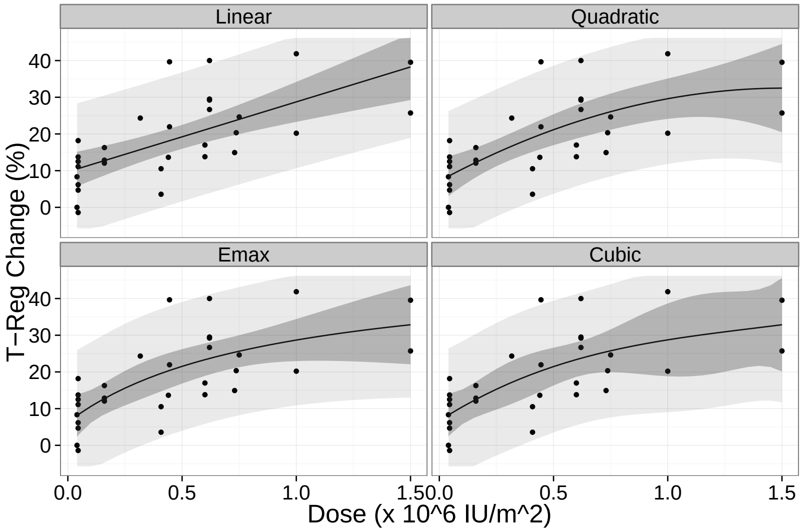

Figure 7: Patient-level predictions with 95% pointwise confidence intervals

Figure 7 shows the uncertainty in the models about the population average

expected value in the dark gray, identical to figure 6. The light gray bands add on the extra variability due to between-patient variability. If we were to dose a new patient, we are 95% certain their outcome will lie in the light, or dark, gray region. The values shown in figure 7 are tabulated below in table 1

**Model**	**Dose**	**Estimate**	**CI for Mean Value**		**CI for a Patient**	
			lower	upper	lower	upper
Linear	0.04	0.10	0.06	0.15	−0.06	0.28
	0.05	0.11	0.06	0.15	−0.06	0.29
	0.40	0.17	0.14	0.21	0.00	0.35
	0.45	0.18	0.15	0.22	0.01	0.36
	0.50	0.19	0.16	0.22	0.02	0.37
	0.55	0.20	0.17	0.23	0.03	0.38
Quadratic	0.04	0.08	0.03	0.14	−0.06	0.26
	0.05	0.09	0.04	0.14	−0.06	0.27
	0.40	0.19	0.15	0.23	0.01	0.36
	0.45	0.20	0.16	0.24	0.03	0.37
	0.50	0.21	0.17	0.25	0.04	0.39
	0.55	0.22	0.18	0.27	0.05	0.40
Emax	0.04	0.08	0.02	0.14	−0.06	0.26
	0.05	0.09	0.03	0.14	−0.06	0.26
	0.40	0.19	0.15	0.24	0.02	0.37
	0.45	0.21	0.16	0.25	0.03	0.38
	0.50	0.22	0.17	0.26	0.04	0.39
	0.55	0.22	0.18	0.27	0.05	0.40
Cubic	0.04	0.08	0.02	0.14	−0.06	0.26
	0.05	0.09	0.03	0.14	−0.06	0.27
	0.40	0.19	0.13	0.25	0.01	0.37
	0.45	0.20	0.15	0.26	0.02	0.38
	0.50	0.21	0.16	0.27	0.03	0.39
	0.55	0.22	0.18	0.27	0.05	0.40

Table 1: Modelling Uncertainty

4 Residual Plots for the fitted Models

Standard residual plots in figures 8, 9.

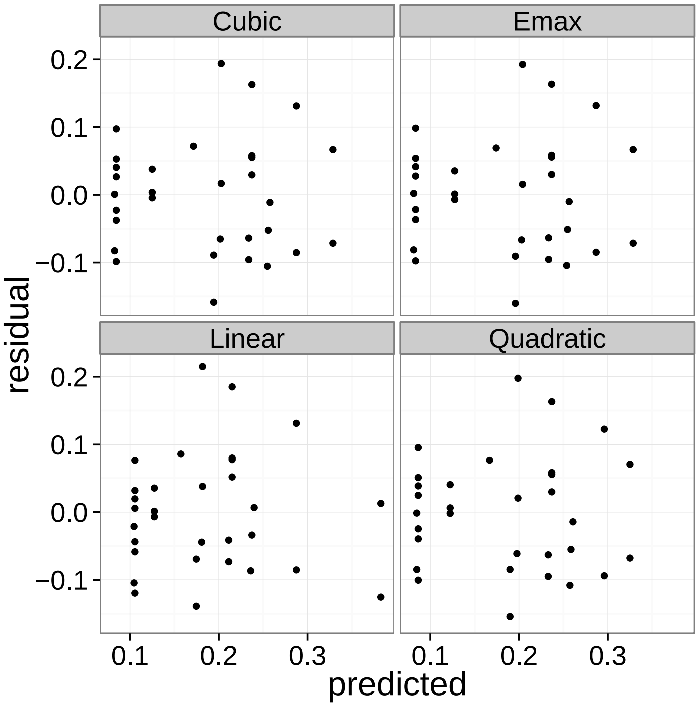

Figure 8: Residual versus Predicted Value

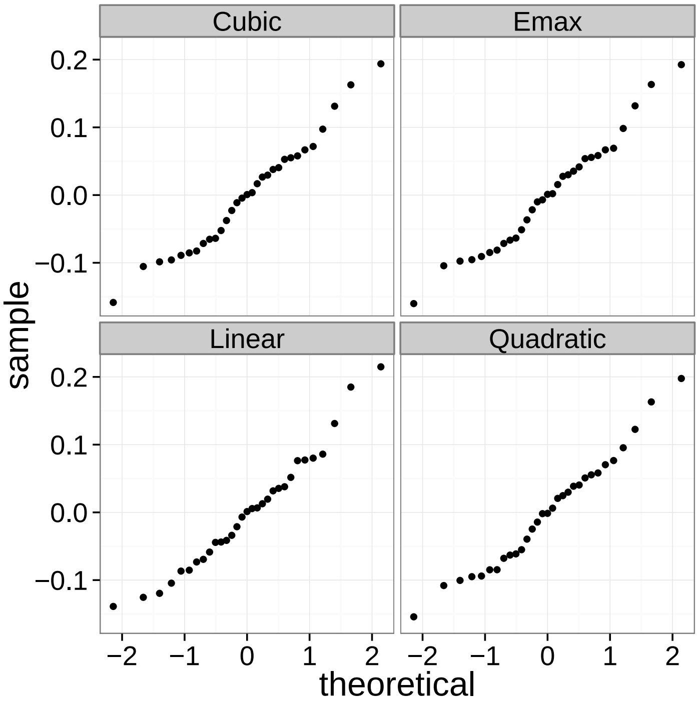

Figure 9: Quantile-Quantile Plot for Residuals

5 Details of Models and Estimated Target Doses

The fitted model details and predicted targets are summarised in table 2.

Table 2: Table of Predicted Target Doses, 95% C.I. and Goodness-of-Fit measures

**Model**	**Target 1**		**Target 2**			
	Estimate (SE)	95% C.I.	Estimate (SE)	95% C.I.	Deviance	AIC
linear	0.0162 (0.124)	-0.227, 0.260	0.541 (0.0850)	0.375, 0.708	0.226	-58.6
quadratic	0.0874 (0.0734)	-0.0564, 0.231	0.450 (0.0834)	0.286, 0.613	0.210	-58.8
Emax	0.0851 (0.0575)	-0.0277, 0.198	0.426 (0.109)	0.212, 0.641	0.210	-58.9
cubic	0.0871 (0.0659)	-0.0420, 0.216	0.433 (0.120)	0.198, 0.668	0.210	-56.9

6 Dosing

- There are patients currently in the study on these doses: 0.175.
- The number of doses to be chosen is: 1.
- The doses must lie between 0.045 and 1.5
- The target levels are: 0.1, 0.2

The recommended dose for each of the convergent models is given in table 3

Table 3: Recommended Doses by Model

**Model**	**Recomended Dose(s)**
linear	1.50
quadratic	0.0450
Emax	0.0450
cubic	0.365

Table 4: Sensitivity Analysis to Dose Choice

	Recomended Doses	Decrease in CR Area (%)			
		linear	quadratic	Emax	cubic
linear	1.500	10.6	0.9	1.5	0.0
quadratic	0.045	3.2	4.2	4.6	4.5
Emax	0.045	3.2	4.2	4.6	4.5
cubic	0.365	1.7	2.1	3.6	4.8

7 Complete R Estimation Details

The output summarising the fitted models is given below

```
$linear
$fit
Nonlinear regression model
  model: y ~ f(dose, a, b)
   data: data
      a       b
0.09691 0.19046
 residual sum-of-squares: 0.2261

Number of iterations to convergence: 1
Achieved convergence tolerance: 6.218e-17

$model
[1] "linear"

$fitSummary

Formula: y ~ f(dose, a, b)

Parameters:
  Estimate Std. Error t value Pr(>|t|)
a  0.09691    0.02412   4.017 0.000382 ***
b  0.19046    0.03958   4.812 4.28e-05 ***
---
Signif. codes:  0 '***' 0.001 '**' 0.01 '*' 0.05 '.' 0.1 ' ' 1

Residual standard error: 0.0883 on 29 degrees of freedom

Number of iterations to convergence: 1
Achieved convergence tolerance: 6.218e-17
```

```
$dhat

0.01622152 0.54125766

$dhatCov
           [,1]        [,2]
[1,] 0.01540853 0.005363680
[2,] 0.00536368 0.007224569

$CurrentDose
[1] 0.175

$NextDose
[1] 1.5

$target
[1] 0.1 0.2

$data
    Group.1 patid baseTreg                    dose time treg id       Dtreg
1         1     1      5.4                   0.040    7  5.4  1  0.000000
2         2     2      7.2                   0.040    7  7.8  7  8.333333
3         3     3      7.0                   0.160    7  7.9  8 12.857143
4         4     4      4.3                   0.160    7  5.0  9 16.279070
5         5     5      8.7                   0.600    7  9.9 10 13.793103
6         6     6      5.3                   0.600    7  6.2 11 16.981132
7         7     7      4.3                   1.000    7  6.1 12 41.860465
8         8     8      9.9                   1.000    7 11.9 13 20.202020
9        10    10      7.0                   1.500    7  8.8 15 25.714286
10       11    11      4.3                   1.500    7  6.0 16 39.534884
11       12    12      6.5                   0.620    7  8.4 17 29.230769
12       14    14      7.5                   0.620    7  9.5 19 26.666667
13       15    15      4.4                   0.440    7  5.0 20 13.636364
14       16    16      6.9                   0.750    7  8.6 21 24.637681
15       17    17      6.6                   0.045    7  7.8 22 18.181818
16       18    18      8.3                   0.160    7  9.3 23 12.048193
17       19    19      4.8                   0.045    7  5.4 24 12.500000
18       20    20      6.7                   0.730    7  7.7 25 14.925373
19       21    21      8.1                   0.045    7  8.6 26  6.172840
20       23    23      6.1                   0.620    7  7.9 28 29.508197
21       24    24      6.4                   0.045    7  6.7 29  4.687500
22       25    25      5.7                   0.408    7  6.3 30 10.526316
23       26    26      3.5                   0.620    7  4.9 31 40.000000
24       27    27      8.4                   0.408    7  8.7 32  3.571429
25       28    28      7.4                   0.317    7  9.2 33 24.324324
```

```
26       29    29      5.8                  0.445    7  8.1 34 39.655172
27       30    30      6.3                  0.045    7  7.0 36 11.111111
28       31    31      8.2                  0.445    7 10.0 37 21.951220
29       32    32      5.1                  0.045    7  5.8 38 13.725490
30       34    34      7.1                  0.045    7  7.0 40 -1.408451
31       35    35      5.9                  0.737    7  7.1 41 20.338983
              dosetext           y
1   0.04 x 10^6 IU/m^2  0.00000000
2   0.04 x 10^6 IU/m^2  0.08333333
3   0.16 x 10^6 IU/m^2  0.12857143
4   0.16 x 10^6 IU/m^2  0.16279070
5    0.6 x 10^6 IU/m^2  0.13793103
6    0.6 x 10^6 IU/m^2  0.16981132
7      1 x 10^6 IU/m^2  0.41860465
8      1 x 10^6 IU/m^2  0.20202020
9    1.5 x 10^6 IU/m^2  0.25714286
10   1.5 x 10^6 IU/m^2  0.39534884
11  0.62 x 10^6 IU/m^2  0.29230769
12  0.62 x 10^6 IU/m^2  0.26666667
13  0.44 x 10^6 IU/m^2  0.13636364
14  0.75 x 10^6 IU/m^2  0.24637681
15 0.045 x 10^6 IU/m^2  0.18181818
16  0.16 x 10^6 IU/m^2  0.12048193
17 0.045 x 10^6 IU/m^2  0.12500000
18  0.73 x 10^6 IU/m^2  0.14925373
19 0.045 x 10^6 IU/m^2  0.06172840
20  0.62 x 10^6 IU/m^2  0.29508197
21 0.045 x 10^6 IU/m^2  0.04687500
22 0.408 x 10^6 IU/m^2  0.10526316
23  0.62 x 10^6 IU/m^2  0.40000000
24 0.408 x 10^6 IU/m^2  0.03571429
25 0.317 x 10^6 IU/m^2  0.24324324
26 0.445 x 10^6 IU/m^2  0.39655172
27 0.045 x 10^6 IU/m^2  0.11111111
28 0.445 x 10^6 IU/m^2  0.21951220
29 0.045 x 10^6 IU/m^2  0.13725490
30 0.045 x 10^6 IU/m^2 -0.01408451
31 0.737 x 10^6 IU/m^2  0.20338983

$lowDose
[1] 0.045

$hiDose
[1] 1.5

$optimResult
```

```
$optimResult$par
[1] 1.5

$optimResult$value
[1] -9.671962

$optimResult$counts
function gradient
       1        1

$optimResult$convergence
[1] 0

$optimResult$message
[1] "CONVERGENCE: NORM OF PROJECTED GRADIENT <= PGTOL"


$betaCovariance
              a             b
a  0.0005818912 -0.0007194411
b -0.0007194411  0.0015667492

$sigma
[1] 0.08830279

$f
function (dose, a, b)
{
    .value <- a + b * dose
    .grad <- array(0, c(length(.value), 2L), list(NULL, c("a",
        "b")))
    .hessian <- array(0, c(length(.value), 2L, 2L), list(NULL,
        c("a", "b"), c("a", "b")))
    .grad[, "a"] <- 1
    .grad[, "b"] <- dose
    attr(.value, "gradient") <- .grad
    attr(.value, "hessian") <- .hessian
    .value
}

$g
function (dose, a, b)
{
    .value <- a + b * dose
    .grad <- array(0, c(length(.value), 1L), list(NULL, c("dose")))
    .hessian <- array(0, c(length(.value), 1L, 1L), list(NULL,
```

```
        c("dose"), c("dose")))
    .grad[, "dose"] <- b
    attr(.value, "gradient") <- .grad
    attr(.value, "hessian") <- .hessian
    .value
}

$Optimality
[1] "Det"

attr(,"class")
[1] "NextDose"

$quadratic
$fit
Nonlinear regression model
  model: y ~ f(dose, a, b, c)
   data: data
       a        b        c
 0.07153  0.33557 -0.11107
 residual sum-of-squares: 0.2103

Number of iterations to convergence: 1
Achieved convergence tolerance: 6.71e-17

$model
[1] "quadratic"

$fitSummary

Formula: y ~ f(dose, a, b, c)

Parameters:
  Estimate Std. Error t value Pr(>|t|)
a  0.07153    0.02943   2.431  0.02172 *
b  0.33557    0.10720   3.130  0.00406 **
c -0.11107    0.07648  -1.452  0.15755
---
Signif. codes:  0 '***' 0.001 '**' 0.01 '*' 0.05 '.' 0.1 ' ' 1

Residual standard error: 0.08666 on 28 degrees of freedom

Number of iterations to convergence: 1
Achieved convergence tolerance: 6.71e-17
```

```
$dhat

0.08736582 0.44981054

$dhatCov
            [,1]        [,2]
[1,] 0.005381853 0.001820379
[2,] 0.001820379 0.006955536

$CurrentDose
[1] 0.175

$NextDose
[1] 0.045

$target
[1] 0.1 0.2

$data
   Group.1 patid baseTreg                    dose time treg id     Dtreg
1        1     1      5.4                   0.040    7  5.4  1  0.000000
2        2     2      7.2                   0.040    7  7.8  7  8.333333
3        3     3      7.0                   0.160    7  7.9  8 12.857143
4        4     4      4.3                   0.160    7  5.0  9 16.279070
5        5     5      8.7                   0.600    7  9.9 10 13.793103
6        6     6      5.3                   0.600    7  6.2 11 16.981132
7        7     7      4.3                   1.000    7  6.1 12 41.860465
8        8     8      9.9                   1.000    7 11.9 13 20.202020
9       10    10      7.0                   1.500    7  8.8 15 25.714286
10      11    11      4.3                   1.500    7  6.0 16 39.534884
11      12    12      6.5                   0.620    7  8.4 17 29.230769
12      14    14      7.5                   0.620    7  9.5 19 26.666667
13      15    15      4.4                   0.440    7  5.0 20 13.636364
14      16    16      6.9                   0.750    7  8.6 21 24.637681
15      17    17      6.6                   0.045    7  7.8 22 18.181818
16      18    18      8.3                   0.160    7  9.3 23 12.048193
17      19    19      4.8                   0.045    7  5.4 24 12.500000
18      20    20      6.7                   0.730    7  7.7 25 14.925373
19      21    21      8.1                   0.045    7  8.6 26  6.172840
20      23    23      6.1                   0.620    7  7.9 28 29.508197
21      24    24      6.4                   0.045    7  6.7 29  4.687500
22      25    25      5.7                   0.408    7  6.3 30 10.526316
23      26    26      3.5                   0.620    7  4.9 31 40.000000
24      27    27      8.4                   0.408    7  8.7 32  3.571429
25      28    28      7.4                   0.317    7  9.2 33 24.324324
26      29    29      5.8                   0.445    7  8.1 34 39.655172
```

```
27      30    30      6.3                      0.045     7  7.0 36 11.111111
28      31    31      8.2                      0.445     7 10.0 37 21.951220
29      32    32      5.1                      0.045     7  5.8 38 13.725490
30      34    34      7.1                      0.045     7  7.0 40 -1.408451
31      35    35      5.9                      0.737     7  7.1 41 20.338983
              dosetext           y
1   0.04 x 10^6 IU/m^2  0.00000000
2   0.04 x 10^6 IU/m^2  0.08333333
3   0.16 x 10^6 IU/m^2  0.12857143
4   0.16 x 10^6 IU/m^2  0.16279070
5    0.6 x 10^6 IU/m^2  0.13793103
6    0.6 x 10^6 IU/m^2  0.16981132
7      1 x 10^6 IU/m^2  0.41860465
8      1 x 10^6 IU/m^2  0.20202020
9    1.5 x 10^6 IU/m^2  0.25714286
10   1.5 x 10^6 IU/m^2  0.39534884
11  0.62 x 10^6 IU/m^2  0.29230769
12  0.62 x 10^6 IU/m^2  0.26666667
13  0.44 x 10^6 IU/m^2  0.13636364
14  0.75 x 10^6 IU/m^2  0.24637681
15 0.045 x 10^6 IU/m^2  0.18181818
16  0.16 x 10^6 IU/m^2  0.12048193
17 0.045 x 10^6 IU/m^2  0.12500000
18  0.73 x 10^6 IU/m^2  0.14925373
19 0.045 x 10^6 IU/m^2  0.06172840
20  0.62 x 10^6 IU/m^2  0.29508197
21 0.045 x 10^6 IU/m^2  0.04687500
22 0.408 x 10^6 IU/m^2  0.10526316
23  0.62 x 10^6 IU/m^2  0.40000000
24 0.408 x 10^6 IU/m^2  0.03571429
25 0.317 x 10^6 IU/m^2  0.24324324
26 0.445 x 10^6 IU/m^2  0.39655172
27 0.045 x 10^6 IU/m^2  0.11111111
28 0.445 x 10^6 IU/m^2  0.21951220
29 0.045 x 10^6 IU/m^2  0.13725490
30 0.045 x 10^6 IU/m^2 -0.01408451
31 0.737 x 10^6 IU/m^2  0.20338983

$lowDose
[1] 0.045

$hiDose
[1] 1.5

$optimResult
$optimResult$par
```

```
[1] 0.045

$optimResult$value
[1] -10.41625

$optimResult$counts
function gradient
       1        1

$optimResult$convergence
[1] 0

$optimResult$message
[1] "CONVERGENCE: NORM OF PROJECTED GRADIENT <= PGTOL"


$betaCovariance
              a            b            c
a  0.0008658801 -0.002439146  0.001336628
b -0.0024391463  0.011492711 -0.007641976
c  0.0013366279 -0.007641976  0.005849532

$sigma
[1] 0.0866613

$f
function (dose, a, b, c)
{
    .expr3 <- dose^2
    .value <- a + b * dose + c * .expr3
    .grad <- array(0, c(length(.value), 3L), list(NULL, c("a",
        "b", "c")))
    .hessian <- array(0, c(length(.value), 3L, 3L), list(NULL,
        c("a", "b", "c"), c("a", "b", "c")))
    .grad[, "a"] <- 1
    .grad[, "b"] <- dose
    .grad[, "c"] <- .expr3
    .hessian[, "c", "c"] <- 0
    attr(.value, "gradient") <- .grad
    attr(.value, "hessian") <- .hessian
    .value
}

$g
function (dose, a, b, c)
{
```

```
    .value <- a + b * dose + c * dose^2
    .grad <- array(0, c(length(.value), 1L), list(NULL, c("dose")))
    .hessian <- array(0, c(length(.value), 1L, 1L), list(NULL,
        c("dose"), c("dose")))
    .grad[, "dose"] <- b + c * (2 * dose)
    .hessian[, "dose", "dose"] <- c * 2
    attr(.value, "gradient") <- .grad
    attr(.value, "hessian") <- .hessian
    .value
}

$Optimality
[1] "Det"

attr(,"class")
[1] "NextDose"

$Emax
$fit
Nonlinear regression model
  model: y ~ f(dose, a, b, c)
   data: data
      a       b       c
0.06314 0.47449 0.89211
 residual sum-of-squares: 0.2097

Number of iterations to convergence: 3
Achieved convergence tolerance: 6.223e-06

$model
[1] "Emax"

$fitSummary

Formula: y ~ f(dose, a, b, c)

Parameters:
  Estimate Std. Error t value Pr(>|t|)
a  0.06314    0.03698   1.707   0.0988 .
b  0.47449    0.33953   1.397   0.1732
c  0.89211    1.07001   0.834   0.4115
---
Signif. codes:  0 '***' 0.001 '**' 0.01 '*' 0.05 '.' 0.1 ' ' 1

Residual standard error: 0.08654 on 28 degrees of freedom
```

```
Number of iterations to convergence: 3
Achieved convergence tolerance: 6.223e-06


$dhat

0.08510266 0.42627256

$dhatCov
            [,1]        [,2]
[1,] 0.003310277 0.001745466
[2,] 0.001745466 0.011973825

$CurrentDose
[1] 0.175

$NextDose
[1] 0.045

$target
[1] 0.1 0.2

$data
```

	Group.1	patid	baseTreg	dose	time	treg	id	Dtreg
1	1	1	5.4	0.040	7	5.4	1	0.000000
2	2	2	7.2	0.040	7	7.8	7	8.333333
3	3	3	7.0	0.160	7	7.9	8	12.857143
4	4	4	4.3	0.160	7	5.0	9	16.279070
5	5	5	8.7	0.600	7	9.9	10	13.793103
6	6	6	5.3	0.600	7	6.2	11	16.981132
7	7	7	4.3	1.000	7	6.1	12	41.860465
8	8	8	9.9	1.000	7	11.9	13	20.202020
9	10	10	7.0	1.500	7	8.8	15	25.714286
10	11	11	4.3	1.500	7	6.0	16	39.534884
11	12	12	6.5	0.620	7	8.4	17	29.230769
12	14	14	7.5	0.620	7	9.5	19	26.666667
13	15	15	4.4	0.440	7	5.0	20	13.636364
14	16	16	6.9	0.750	7	8.6	21	24.637681
15	17	17	6.6	0.045	7	7.8	22	18.181818
16	18	18	8.3	0.160	7	9.3	23	12.048193
17	19	19	4.8	0.045	7	5.4	24	12.500000
18	20	20	6.7	0.730	7	7.7	25	14.925373
19	21	21	8.1	0.045	7	8.6	26	6.172840
20	23	23	6.1	0.620	7	7.9	28	29.508197
21	24	24	6.4	0.045	7	6.7	29	4.687500
22	25	25	5.7	0.408	7	6.3	30	10.526316

```
23      26    26      3.5                0.620     7  4.9 31 40.000000
24      27    27      8.4                0.408     7  8.7 32  3.571429
25      28    28      7.4                0.317     7  9.2 33 24.324324
26      29    29      5.8                0.445     7  8.1 34 39.655172
27      30    30      6.3                0.045     7  7.0 36 11.111111
28      31    31      8.2                0.445     7 10.0 37 21.951220
29      32    32      5.1                0.045     7  5.8 38 13.725490
30      34    34      7.1                0.045     7  7.0 40 -1.408451
31      35    35      5.9                0.737     7  7.1 41 20.338983
              dosetext           y
1   0.04 x 10^6 IU/m^2  0.00000000
2   0.04 x 10^6 IU/m^2  0.08333333
3   0.16 x 10^6 IU/m^2  0.12857143
4   0.16 x 10^6 IU/m^2  0.16279070
5    0.6 x 10^6 IU/m^2  0.13793103
6    0.6 x 10^6 IU/m^2  0.16981132
7      1 x 10^6 IU/m^2  0.41860465
8      1 x 10^6 IU/m^2  0.20202020
9    1.5 x 10^6 IU/m^2  0.25714286
10   1.5 x 10^6 IU/m^2  0.39534884
11  0.62 x 10^6 IU/m^2  0.29230769
12  0.62 x 10^6 IU/m^2  0.26666667
13  0.44 x 10^6 IU/m^2  0.13636364
14  0.75 x 10^6 IU/m^2  0.24637681
15 0.045 x 10^6 IU/m^2  0.18181818
16  0.16 x 10^6 IU/m^2  0.12048193
17 0.045 x 10^6 IU/m^2  0.12500000
18  0.73 x 10^6 IU/m^2  0.14925373
19 0.045 x 10^6 IU/m^2  0.06172840
20  0.62 x 10^6 IU/m^2  0.29508197
21 0.045 x 10^6 IU/m^2  0.04687500
22 0.408 x 10^6 IU/m^2  0.10526316
23  0.62 x 10^6 IU/m^2  0.40000000
24 0.408 x 10^6 IU/m^2  0.03571429
25 0.317 x 10^6 IU/m^2  0.24324324
26 0.445 x 10^6 IU/m^2  0.39655172
27 0.045 x 10^6 IU/m^2  0.11111111
28 0.445 x 10^6 IU/m^2  0.21951220
29 0.045 x 10^6 IU/m^2  0.13725490
30 0.045 x 10^6 IU/m^2 -0.01408451
31 0.737 x 10^6 IU/m^2  0.20338983

$lowDose
[1] 0.045

$hiDose
```

```
[1] 1.5

$optimResult
$optimResult$par
[1] 0.045

$optimResult$value
[1] -10.36596

$optimResult$counts
function gradient
       1        1

$optimResult$convergence
[1] 0

$optimResult$message
[1] "CONVERGENCE: NORM OF PROJECTED GRADIENT <= PGTOL"


$betaCovariance
             a           b           c
a  0.001393592 -0.01032456  0.02665438
b -0.010324561  0.12017825 -0.36559735
c  0.026654380 -0.36559735  1.19747318

$sigma
[1] 0.08653605

$f
function (dose, a, b, c)
{
    .expr1 <- b * dose
    .expr3 <- 1 + dose/c
    .expr7 <- c^2
    .expr8 <- dose/.expr7
    .expr10 <- .expr3^2
    .expr12 <- .expr1 * .expr8
    .value <- a + .expr1/.expr3
    .grad <- array(0, c(length(.value), 3L), list(NULL, c("a",
        "b", "c")))
    .hessian <- array(0, c(length(.value), 3L, 3L), list(NULL,
        c("a", "b", "c"), c("a", "b", "c")))
    .grad[, "a"] <- 1
    .grad[, "b"] <- dose/.expr3
    .hessian[, "b", "b"] <- 0
```

```
    .hessian[, "b", "c"] <- .hessian[, "c", "b"] <- dose * .expr8/.expr10
    .grad[, "c"] <- .expr12/.expr10
    .hessian[, "c", "c"] <- -(.expr1 * (dose * (2 * c)/.expr7^2)/.expr10 -
        .expr12 * (2 * (.expr8 * .expr3))/.expr10^2)
    attr(.value, "gradient") <- .grad
    attr(.value, "hessian") <- .hessian
    .value
}

$g
function (dose, a, b, c)
{
    .expr1 <- b * dose
    .expr3 <- 1 + dose/c
    .expr7 <- 1/c
    .expr8 <- .expr1 * .expr7
    .expr9 <- .expr3^2
    .expr13 <- b * .expr7/.expr9
    .value <- a + .expr1/.expr3
    .grad <- array(0, c(length(.value), 1L), list(NULL, c("dose")))
    .hessian <- array(0, c(length(.value), 1L, 1L), list(NULL,
        c("dose"), c("dose")))
    .grad[, "dose"] <- b/.expr3 - .expr8/.expr9
    .hessian[, "dose", "dose"] <- -(.expr13 + (.expr13 - .expr8 *
        (2 * (.expr7 * .expr3))/.expr9^2))
    attr(.value, "gradient") <- .grad
    attr(.value, "hessian") <- .hessian
    .value
}

$Optimality
[1] "Det"

attr(,"class")
[1] "NextDose"

$cubic
$fit
Nonlinear regression model
  model: y ~ f(dose, a, b, c, d)
   data: data
       a        b        c        d
 0.06705  0.39829 -0.23523  0.05729
 residual sum-of-squares: 0.2099

Number of iterations to convergence: 1
```

```
Achieved convergence tolerance: 2.418e-16

$model
[1] "cubic"

$fitSummary

Formula: y ~ f(dose, a, b, c, d)

Parameters:
  Estimate Std. Error t value Pr(>|t|)
a  0.06705    0.03643   1.840   0.0767 .
b  0.39829    0.31052   1.283   0.2105
c -0.23523    0.58072  -0.405   0.6886
d  0.05729    0.26557   0.216   0.8308
---
Signif. codes:  0 '***' 0.001 '**' 0.01 '*' 0.05 '.' 0.1 ' ' 1

Residual standard error: 0.08818 on 27 degrees of freedom

Number of iterations to convergence: 1
Achieved convergence tolerance: 2.418e-16


$dhat

0.08708264 0.43273202

$dhatCov
            [,1]        [,2]
[1,] 0.004337504 0.001994908
[2,] 0.001994908 0.014394161

$CurrentDose
[1] 0.175

$NextDose
[1] 0.3652248

$target
[1] 0.1 0.2

$data
    Group.1 patid baseTreg                      dose time treg id      Dtreg
1         1     1      5.4                     0.040    7  5.4  1  0.000000
2         2     2      7.2                     0.040    7  7.8  7  8.333333
```

3	3	3	7.0	0.160	7	7.9	8	12.857143
4	4	4	4.3	0.160	7	5.0	9	16.279070
5	5	5	8.7	0.600	7	9.9	10	13.793103
6	6	6	5.3	0.600	7	6.2	11	16.981132
7	7	7	4.3	1.000	7	6.1	12	41.860465
8	8	8	9.9	1.000	7	11.9	13	20.202020
9	10	10	7.0	1.500	7	8.8	15	25.714286
10	11	11	4.3	1.500	7	6.0	16	39.534884
11	12	12	6.5	0.620	7	8.4	17	29.230769
12	14	14	7.5	0.620	7	9.5	19	26.666667
13	15	15	4.4	0.440	7	5.0	20	13.636364
14	16	16	6.9	0.750	7	8.6	21	24.637681
15	17	17	6.6	0.045	7	7.8	22	18.181818
16	18	18	8.3	0.160	7	9.3	23	12.048193
17	19	19	4.8	0.045	7	5.4	24	12.500000
18	20	20	6.7	0.730	7	7.7	25	14.925373
19	21	21	8.1	0.045	7	8.6	26	6.172840
20	23	23	6.1	0.620	7	7.9	28	29.508197
21	24	24	6.4	0.045	7	6.7	29	4.687500
22	25	25	5.7	0.408	7	6.3	30	10.526316
23	26	26	3.5	0.620	7	4.9	31	40.000000
24	27	27	8.4	0.408	7	8.7	32	3.571429
25	28	28	7.4	0.317	7	9.2	33	24.324324
26	29	29	5.8	0.445	7	8.1	34	39.655172
27	30	30	6.3	0.045	7	7.0	36	11.111111
28	31	31	8.2	0.445	7	10.0	37	21.951220
29	32	32	5.1	0.045	7	5.8	38	13.725490
30	34	34	7.1	0.045	7	7.0	40	-1.408451
31	35	35	5.9	0.737	7	7.1	41	20.338983

	dosetext	y
1	0.04 x 10^6 IU/m^2	0.00000000
2	0.04 x 10^6 IU/m^2	0.08333333
3	0.16 x 10^6 IU/m^2	0.12857143
4	0.16 x 10^6 IU/m^2	0.16279070
5	0.6 x 10^6 IU/m^2	0.13793103
6	0.6 x 10^6 IU/m^2	0.16981132
7	1 x 10^6 IU/m^2	0.41860465
8	1 x 10^6 IU/m^2	0.20202020
9	1.5 x 10^6 IU/m^2	0.25714286
10	1.5 x 10^6 IU/m^2	0.39534884
11	0.62 x 10^6 IU/m^2	0.29230769
12	0.62 x 10^6 IU/m^2	0.26666667
13	0.44 x 10^6 IU/m^2	0.13636364
14	0.75 x 10^6 IU/m^2	0.24637681
15	0.045 x 10^6 IU/m^2	0.18181818
16	0.16 x 10^6 IU/m^2	0.12048193

```
17 0.045 x 10^6 IU/m^2  0.12500000
18  0.73 x 10^6 IU/m^2  0.14925373
19 0.045 x 10^6 IU/m^2  0.06172840
20  0.62 x 10^6 IU/m^2  0.29508197
21 0.045 x 10^6 IU/m^2  0.04687500
22 0.408 x 10^6 IU/m^2  0.10526316
23  0.62 x 10^6 IU/m^2  0.40000000
24 0.408 x 10^6 IU/m^2  0.03571429
25 0.317 x 10^6 IU/m^2  0.24324324
26 0.445 x 10^6 IU/m^2  0.39655172
27 0.045 x 10^6 IU/m^2  0.11111111
28 0.445 x 10^6 IU/m^2  0.21951220
29 0.045 x 10^6 IU/m^2  0.13725490
30 0.045 x 10^6 IU/m^2 -0.01408451
31 0.737 x 10^6 IU/m^2  0.20338983

$lowDose
[1] 0.045

$hiDose
[1] 1.5

$optimResult
$optimResult$par
[1] 0.3652248

$optimResult$value
[1] -9.907959

$optimResult$counts
function gradient
      17       17

$optimResult$convergence
[1] 0

$optimResult$message
[1] "CONVERGENCE: REL_REDUCTION_OF_F <= FACTR*EPSMCH"


$betaCovariance
             a            b           c           d
a  0.001327400 -0.008560918  0.01333106 -0.00551340
b -0.008560918  0.096424759 -0.17522500  0.07721125
c  0.013331059 -0.175225002  0.33723847 -0.15283294
d -0.005513400  0.077211254 -0.15283294  0.07052876
```

```
$sigma
[1] 0.08817558

$f
function (dose, a, b, c, d)
{
    .expr3 <- dose^2
    .expr6 <- dose^3
    .value <- a + b * dose + c * .expr3 + d * .expr6
    .grad <- array(0, c(length(.value), 4L), list(NULL, c("a",
        "b", "c", "d")))
    .hessian <- array(0, c(length(.value), 4L, 4L), list(NULL,
        c("a", "b", "c", "d"), c("a", "b", "c", "d")))
    .grad[, "a"] <- 1
    .grad[, "b"] <- dose
    .grad[, "c"] <- .expr3
    .hessian[, "c", "c"] <- 0
    .hessian[, "c", "d"] <- .hessian[, "d", "c"] <- 0
    .grad[, "d"] <- .expr6
    .hessian[, "d", "d"] <- 0
    attr(.value, "gradient") <- .grad
    attr(.value, "hessian") <- .hessian
    .value
}

$g
function (dose, a, b, c, d)
{
    .expr3 <- dose^2
    .expr9 <- 2 * dose
    .value <- a + b * dose + c * .expr3 + d * dose^3
    .grad <- array(0, c(length(.value), 1L), list(NULL, c("dose")))
    .hessian <- array(0, c(length(.value), 1L, 1L), list(NULL,
        c("dose"), c("dose")))
    .grad[, "dose"] <- b + c * .expr9 + d * (3 * .expr3)
    .hessian[, "dose", "dose"] <- c * 2 + d * (3 * .expr9)
    attr(.value, "gradient") <- .grad
    attr(.value, "hessian") <- .hessian
    .value
}

$Optimality
[1] "Det"

attr(,"class")
```

```
[1] "NextDose"
```
