# Supplementary material for: Regulatory T Cell Responses in Participants with Type 1 Diabetes after a Single Dose of Interleukin-2: A Non-Randomised, Open Label, Adaptive Dose-Finding Trial
Source: PLoS Med. 2016 Oct 11;13(10):e1002139. doi: 10.1371/journal.pmed.1002139 (PMC5058548; doi:10.1371/journal.pmed.1002139)
Supplement: S1 Table — (PDF) [file pmed.1002139.s031.pdf]

S1 Table. Antibody combinations for surface (tubes 1-6) and intra-cellular staining (tube 7)

Tube Numbers		1	2	3	4	5	6	7
Brilliant Stain Buffer		50	50	50	50	50	50	50
Fluorochrome	APC	CD25	CD25	CD25	CD25	CD25	CD25	CD25
	AF700	CD4	CD4	CD4	CD4	CD4	CD4	CD4
	BV785	CD45RA	CD45RA	CD45RA	CD45RA	CD45RA	CD45RA	CD45RA
	PE/Cy7	CD127	CD127	CD127	CD127	CD127	-	CD127
	PerCP/Cy5.5	CXCR3	CXCR3	CXCR3	CXCR3	CXCR3	CD69	KI67
	BV421	-	-	CCR4 (CD194)	-	CCR7	CD56	-
	PB	HLADR	HLADR	-	CD14	-	-	FOXP3
	FITC	-	-	-	-	CD31	TCRab	HELIOS
	AF488	CCR6	CCR6	CCR6	CCR6	-	-	-
	PE	CTLA4 (CD152)	CD69	CCR10	IL6R	CD122	CD122	CTLA4 (CD152)
	APC/Cy7	CD8	CD8	CD8	CD8	CD8	CD8	CD8
	eFluor605/BV605	CD62L	CD62L	CD62L	CD62L	-	CD161	CD62L
	BV510	-	-	-	-	CD3	-	-
