# Supplementary material for: Regulatory T Cell Responses in Participants with Type 1 Diabetes after a Single Dose of Interleukin-2: A Non-Randomised, Open Label, Adaptive Dose-Finding Trial
Source: PLoS Med. 2016 Oct 11;13(10):e1002139. doi: 10.1371/journal.pmed.1002139 (PMC5058548; doi:10.1371/journal.pmed.1002139)
Supplement: S2 Table — (PDF) [file pmed.1002139.s032.pdf]

S2 Table. Detailed antibody/clone information

Antibody	Fluorochrome	Clone	Source	Catalogue no.
CCR10	PE	*	RD Systems	FAB3478P
CCR4 (CD194)	Brilliant Violet 421	IG1	BD Bioscience	562579
CCR6 (CD196)	Alexa Fluor 488	G034E3	BioLegend	353414
CCR7	Brilliant Violet 421	G043H7	BioLegend	353208
CD122	PE	*	BD Bioscience	554525
CD122	PE	TU27	BioLegend	339006
CD127	PE-Cy7	eBioRDR5	eBioscience	25-1278-42
CD14	Pacific Blue	M5E2	BioLegend	301828
CD161	Brilliant Violet 605	HP-3G10	BioLegend	339915
CD25	APC	M-A251	BD Bioscience	555434
CD25	APC	2a3	BD Bioscience	340907
CD3	Brilliant Violet 510	OKT3	BioLegend	317331
CD31	FITC	WM-59	eBioscience	11-0319-42
CD4	Alexa Fluor 700	RPA-T4	BioLegend	300526
CD45RA	Brilliant Violet 785	HI100	BioLegend	304139
CD45RA	Pacific Blue	HI100	BioLegend	304123
CD56	Brilliant Violet 421	HCD56	BioLegend	318327
CD56	PE	HCD56	BioLegend	318306
CD62L	Brilliant Violet 605	DREG-56	eBioscience	93-0629-42
CD69	PE	FN50	BioLegend	310906
CD69	PerCP/Cy5.5	FN50	BioLegend	310926
CD8	APC/Cy7	RPA-T8	BioLegend	301016
CXCR3	PerCP/Cy5.5	G025H7	BioLegend	353714
CXCR5	Alexa Fluor 488	RF8B2	BD Bioscience	558112
GRANZYME A	PE	CB9	BioLegend	507206
HLADR	Pacific Blue	L243	BioLegend	307633
ICOS	PE	C398.4A	BioLegend	313508
IL-6Ra	PE	UV4	BioLegend	352804
PD-1 (CD279)	Pacific Blue	EH12.2h7	BioLegend	329916
TCRab	FITC	IP26	BioLegend	306706
CTLA4 (CD152)	PE		BD Bioscience	555853
FOXP3	Pacific Blue	259D	BioLegend	320216
KI67	PerCP/Cy5.5	B56	BD Bioscience	561284

*Not noted by company
