# Supplementary material for: Regulatory T Cell Responses in Participants with Type 1 Diabetes after a Single Dose of Interleukin-2: A Non-Randomised, Open Label, Adaptive Dose-Finding Trial
Source: PLoS Med. 2016 Oct 11;13(10):e1002139. doi: 10.1371/journal.pmed.1002139 (PMC5058548; doi:10.1371/journal.pmed.1002139)
Supplement: S3 Table — (PDF) [file pmed.1002139.s033.pdf]

S3 Table. Antibody combinations for cell sorting

		Antibody	Sorted Cell			
			**TTreg** (Total T-reg)	**MemTeff** (Mem central)	**62L-MTeff** (Mem effector)	**NK-CD56** (CD56 bright)
**Fluorochrome**	**APC**	CD25 (2 clones)	4 x test volume			
	**Pacific Blue**	CD45RA	4 x test volume			
	**PE**	CD56	4 x test volume			
	**PE/Cy7**	CD127	4 x test volume			
	**BV605/eFluor605**	CD62L	4 x test volume			
	**FITC**	TCRab	2.5 x test volume			
	**AF700**	CD4	4 x test volume			
