# Supplementary material for: Regulatory T Cell Responses in Participants with Type 1 Diabetes after a Single Dose of Interleukin-2: A Non-Randomised, Open Label, Adaptive Dose-Finding Trial
Source: PLoS Med. 2016 Oct 11;13(10):e1002139. doi: 10.1371/journal.pmed.1002139 (PMC5058548; doi:10.1371/journal.pmed.1002139)
Supplement: S4 Table — (PDF) [file pmed.1002139.s034.pdf]

S4 Table. Antibody combinations and information for pSTAT5 assay

Fluorochrome	Antibody	Clone	Supplier
**APC**	CD25	2A3, M-A251	BD Biosciences
**AF700**	CD4	RPA-T4	Biolegend
**PE/Cy7**	CD56	HCD56	Biolegend
**PerCP/Cy5.5**	CD3	UCHT1	Biolegend
**PB**	CD45RA	HI100	Biolegend
**AF488**	STAT5a (pY7694)	47	BD Biosciences
**PE**	FoxP3	259D	Biolegend
**eFluor605/BV605**	CD8	RPA-T8	Biolegend
