# Supplementary material for: Regulatory T Cell Responses in Participants with Type 1 Diabetes after a Single Dose of Interleukin-2: A Non-Randomised, Open Label, Adaptive Dose-Finding Trial
Source: PLoS Med. 2016 Oct 11;13(10):e1002139. doi: 10.1371/journal.pmed.1002139 (PMC5058548; doi:10.1371/journal.pmed.1002139)
Supplement: S5 Table — (PDF) [file pmed.1002139.s035.pdf]

S5 Table. Full blood counts baseline, day 1 and final visit

	Baseline (N=40)	Day 1 (N=40)	Final visit (N=38)	Normal range
	Mean (SE, Range)	Mean (SE, Range)	Mean (SE, Range)	
WBC (10^9/l)	6.11 (0.40, 3.9–18.9)	5.61 (0.18, 3.4-8.6)	5.98 (0.27, 3.7-12.6)	4.00 - 11.00
RBC (10^{12}/l)	4.75 (0.07, 3.96-5.66)	4.77 (0.07, 3.98-5.79)	4.74 (0.07, 3.98-5.90)	4.20 - 5.80
Hb (g/l)	145.53 (1.96, 109-164)	146.25 (2.03, 110-172)	145.26 (2.22, 105-173)	130.00 - 170.00
Haematocrit (l/l)	0.43 (0.01, 0.34-0.49)	0.43 (0.01, 0.34-0.49)	0.42 (0.01, 0.32-0.51)	0.39 - 0.50
MCV (fl)	90.05 (0.76, 79.6-100.3)	90.02 (0.79, 79.2-100.7)	89.48 (0.79, 74.1-101.2)	80.00 - 100.00
MCH (pg)	30.70 (0.29, 25.6-33.7)	30.75 (0.29, 25.5-34.1)	30.65 (0.29, 24-33.8)	27.00 - 32.00
Platelets (10^9/l)	198.58 (5.59, 143-268)	199.02 (5.96, 142-272)	214.47 (7.89, 139-306)	150.00 - 400.00
Neutrophils (10^9/l)	3.62 (0.36, 1.68-15.49)	3.30 (0.15, 1.82-5.12)	3.54 (0.24, 1.73-10.02)	2.00 - 8.00
Lymphocytes (10^9/l)	1.78 (0.08, 0.95-3.84)	1.56 (0.06, 0.81-2.89)	1.79 (0.09, 0.86-3.21)	1.00 - 4.50
Monocytes (10^9/l)	0.46 (0.03, 0.22-1.00)	0.44 (0.02, 0.23-0.76)	0.44 (0.02, 0.25-0.68)	0.10 - 0.80
Eosinophils (10^9/l)	0.20 (0.02, 0.03-0.86)	0.25 (0.03, 0.03-0.95)	0.15 (0.01, 0.02-0.36)	0.00 - 0.40
Basophils (10^9/l)	0.04 (0.00, 0.01-0.18)	0.04 (0.00, 0.01-0.12)	0.05 (0.01, 0.01-0.31)	0.00 - 0.30
