# Supplementary material for: Regulatory T Cell Responses in Participants with Type 1 Diabetes after a Single Dose of Interleukin-2: A Non-Randomised, Open Label, Adaptive Dose-Finding Trial
Source: PLoS Med. 2016 Oct 11;13(10):e1002139. doi: 10.1371/journal.pmed.1002139 (PMC5058548; doi:10.1371/journal.pmed.1002139)
Supplement: S6 Table — (PDF) [file pmed.1002139.s036.pdf]

S6 Table. Clinical FACS analysis of TNBK assay at baseline, day 1, and final visit

	Baseline (N=40)	Day 1 (N=40)	Final visit (N=38)	Normal range
	Mean (SE, Range)	Mean (SE, Range)	Mean (SE, Range)	
CD19 (%)	12.63 (0.75, 4-26)	12.30 (0.71, 4-28)	13.76 (0.84, 6-30)	--
CD19 Total B Cells ($x10^9$/l)	0.23 (0.02, 0.07-0.57)	0.19 (0.01, 0.05-0.46)	0.25 (0.02, 0.06-0.62)	0.10 - 0.50
CD3 (%)	77.00 (1.09, 58-89)	76.72 (1.09, 56-86)	76.11 (1.07, 61-88)	--
CD3 Total T Cells ($x10^9$/l)	1.44 (0.07,0.76-3.18)	1.24 (0.06, 0.70-2.46)	1.41 (0.08, 0.7-2.74)	0.70 - 2.10
CD4 (%)	46.40 (1.27, 21-61)	46.23 (1.42, 21-69)	47.68 (1.27, 35-67)	--
CD4 Total ($x10^9$/l)	0.87 (0.05, 0.47-2.08)	0.73 (0.03, 0.42-1.28)	0.88 (0.06, 0.48-1.77)	0.30 - 1.40
CD56 (%)	9.70 (0.77, 3-24)	10.5 (0.82, 5-27)	9.42 (0.70, 3-21)	--
CD56 Total NK Cells ($x10^9$/l)	0.17 (0.01, 0.04-0.42)	0.16 (0.01, 0.08-0.46)	0.16 (0.01, 0.06-0.34)	0.12 - 0.88
CD8 (%)	28.30 (1.18, 18-59)	28.05 (1.29, 17-62)	26.24 (0.80, 17-40)	--
CD8 Total ($x10^9$/l)	0.53 (0.04, 0.26-1.48)	0.46 (0.04, 0.20-1.81)	0.48 (0.03, 0.19-0.90)	0.20 - 0.90
Lymphocyte count ($x10^9$/l)	1.87 (0.09, 1.04-4.08)	1.62 (0.06, 0.89-2.93)	1.85 (0.10, 0.91-3.48)	1.00 - 2.80
TREG (% CD4 T cells)	6.54 (0.25, 3.5-10.7)	6.06 (0.25, 3-9.3)	6.61 (0.19, 4-8.8)	--
TREG Absolute ($x10^9$/l)	0.06 (0.00, 0.02-0.14)	0.04 (0.00, 0.02-0.09)	0.06 (0.00, 0.03-0.13)	--
TREG CD25 (MFI)*	3881.75 (88.28, 2792-5814)	4973.93 (165.56, 2981-8191)	3640.16 (93.11, 2533-5548)	--

*Mean fluorescence intensity
