# Supplementary material for: Regulatory T Cell Responses in Participants with Type 1 Diabetes after a Single Dose of Interleukin-2: A Non-Randomised, Open Label, Adaptive Dose-Finding Trial
Source: PLoS Med. 2016 Oct 11;13(10):e1002139. doi: 10.1371/journal.pmed.1002139 (PMC5058548; doi:10.1371/journal.pmed.1002139)
Supplement: S7 Table — (PDF) [file pmed.1002139.s037.pdf]

S7 Table. Metabolic measures & Thyroid function tests at baseline and final visit

	Baseline		Final visit		Normal range
	N	Mean (SE, Range)	N	Mean (SE, Range)	
Insulin usage (units-bolus)	27	13.5 (1.60, 0-30)	25	13.48 (1.71, 1-36)	--
Insulin usage (units-basal)	40	14.54 (1.91, 0-64)	38	14.22 (1.94, 0-64)	--
SMBG (mmol/l)	39	9.57 (0.82, 2.9-27.6)	37	7.59 (0.39, 3.2-12.7)	--
Glucose (mmol/l)	40	10.05 (0.84, 2.7-27.7)	38	9.19 (0.62, 4-18.7)	--
HbA1C (mmol/l)	40	58.93 (3.52, 34-132)	38	55.37 (3.06, 36-123)	30.00-45.00
C-peptide (pmol/l)	40	483.78 (55.44, 9-1350)	38	450.24 (48.38, 9-1130)	174.00-960.00
TSH (mU/l)	40	2.82 (1.01, 0.45-40.4)	38	2.12 (0.29, 0.38-11.2)	0.35-5.50
T4 (pmol/l)	40	15.38 (0.36, 10.5-21.7)	38	15.32 (0.42, 10.8-21)	10.00-19.80
