# Supplementary material for: Regulatory T Cell Responses in Participants with Type 1 Diabetes after a Single Dose of Interleukin-2: A Non-Randomised, Open Label, Adaptive Dose-Finding Trial
Source: PLoS Med. 2016 Oct 11;13(10):e1002139. doi: 10.1371/journal.pmed.1002139 (PMC5058548; doi:10.1371/journal.pmed.1002139)
Supplement: S8 Table — (PDF) [file pmed.1002139.s038.pdf]

S8 Table. Renal, bone and liver biochemistries at baseline and final visit

	Baseleine		Final visit (N=38)	Normal range
	N	Mean (SE, Range)	Mean (SE, Range)	
Serum Sodium (mmol/l)	40	138.88 (0.42, 132-143)	138.68 (0.30, 135-142)	135.00 - 145.00
Serum Potassium (mmol/l)	40	4.30 (0.04, 3.8-5)	4.29 (0.04, 3.8-4.8)	3.40 - 5.00
Urea (mmol/l)	40	5.45 (0.18, 3.6-8.5)	5.56 (0.20, 3.4-9.3)	0.00 - 7.50
Creatinine (µmol/l)	40	79.08 (2.40, 51-129)	74.90 (2.64, 30-115)	35.00 - 125.00
Albumin (g/L)	40	40.60 (0.53, 33-49)	41.05 (0.56, 31-49)	60.00 - 51.00
Calcium (mmol/L)	40	2.26 (0.01)	2.29 (0.01)	--
Corrected Calcium (mmol/L)	40	2.21 (0.01, 2.05-2.39)	2.23 (0.01, 2.1-2.4)	2.10 - 2.50
T.Bilirubin (µmol/l)	40	10.70 (1.33, 4-54)	11.18 (1.53, 2-64)	0.00 - 17.00
Alkaline Phosphatase (U/l)	40	94.70 4.59, 54-171)	94.18 (5.69, 47-190)	30.00 - 135.00
ALT (U/l)	40	26.93 (1.84, 15-64)	24.16 (1.82, 10-59)	0.00 - 50.00
AST (U/l)	39	18.15 (0.67, 9-31)	19.29 (1.00, 9-44)	0.00 - 40.00
Gamma GT (U/l)	40	22.60 (1.82, 9-69)	21.42 (1.67, 8-60)	0.00 - 51.00
