# Supplementary material for: Regulatory T Cell Responses in Participants with Type 1 Diabetes after a Single Dose of Interleukin-2: A Non-Randomised, Open Label, Adaptive Dose-Finding Trial
Source: PLoS Med. 2016 Oct 11;13(10):e1002139. doi: 10.1371/journal.pmed.1002139 (PMC5058548; doi:10.1371/journal.pmed.1002139)
Supplement: S3 Text — (PDF) [file pmed.1002139.s041.pdf]

NRES Committee East of England - Cambridge Central
Victoria House
Capital Park
Fulbourn
Cambridge
CB21 5XB

Telephone: 01223 597685
Facsimile: 01223 597645

18 February 2013

Dr Frank Waldron-Lynch
Cambridge Institute for Medical Research, University of Cambridge
Hills Road
Cambridge
CB2 0XY

Dear Dr Waldron-Lynch

**Study title:**	**Adaptive study of IL-2 dose on regulatory T cells in type 1 diabetes (DILT1D)**
**REC reference:**	**13/EE/0020**
**Protocol number:**	**DILT1D**
**IRAS project ID:**	**115333**

Thank you for your letter of 04 February 2013, responding to the Committee's request for further information on the above research and submitting revised documentation.

The further information has been considered on behalf of the Committee by the Chair.

We plan to publish your research summary wording for the above study on the NRES website, together with your contact details, unless you expressly withhold permission to do so. Publication will be no earlier than three months from the date of this favourable opinion letter. Should you wish to provide a substitute contact point, require further information, or wish to withhold permission to publish, please contact the Co-ordinator Miss Nicky Storey, Nicky.Storey@eoe.nhs.uk.

Confirmation of ethical opinion

On behalf of the Committee, I am pleased to confirm a favourable ethical opinion for the above research on the basis described in the application form, protocol and supporting documentation as revised, subject to the conditions specified below.

Ethical review of research sites

NHS sites

The favourable opinion applies to all NHS sites taking part in the study, subject to management permission being obtained from the NHS/HSC R&D office prior to the start of the study (see "Conditions of the favourable opinion" below).

Non-NHS sites

The Committee has not yet been notified of the outcome of any site-specific assessment (SSA) for the non-NHS research site(s) taking part in this study. The favourable opinion does not therefore apply to any non-NHS site at present. We will write to you again as soon as one Research Ethics Committee has notified the outcome of a SSA. In the meantime no study procedures should be initiated at non-NHS sites.

Conditions of the favourable opinion

The favourable opinion is subject to the following conditions being met prior to the start of the study.

Management permission or approval must be obtained from each host organisation prior to the start of the study at the site concerned.

Management permission ("R&D approval") should be sought from all NHS organisations involved in the study in accordance with NHS research governance arrangements.

Guidance on applying for NHS permission for research is available in the Integrated Research Application System or at http://www.rdforum.nhs.uk.

Where a NHS organisation's role in the study is limited to identifying and referring potential participants to research sites ("participant identification centre"), guidance should be sought from the R&D office on the information it requires to give permission for this activity.

For non-NHS sites, site management permission should be obtained in accordance with the procedures of the relevant host organisation.

Sponsors are not required to notify the Committee of approvals from host organisations

The 2nd paragraph of the patient invitation letter does not make sense because some of the text is repeated and the Chair would like this paragraph to be rewritten so it is clearer.

It is the responsibility of the sponsor to ensure that all the conditions are complied with before the start of the study or its initiation at a particular site (as applicable).

You should notify the REC in writing once all conditions have been met (except for site approvals from host organisations) and provide copies of any revised documentation with updated version numbers. The REC will acknowledge receipt and provide a final list of the approved documentation for the study, which can be made available to host organisations to facilitate their permission for the study. Failure to provide the final versions to the REC may cause delay in obtaining permissions.

Approved documents

The final list of documents reviewed and approved by the Committee is as follows:

Document	*Version*	*Date*
Covering Letter	Dr Frank Waldron-Lynch	17 December 2012
Evidence of insurance or indemnity	University of Cambridge Finance Division	30 November 2012
GP/Consultant Information Sheets	1.0	06 December 2012
Investigator CV	Dr Frank Waldron-Lynch	10 December 2012
Other: Participant card	1.1	27 November 2012
Other: copy award letter from The Wellcome Trust	Dr Emma Hudson	04 June 2010
Other: Patient Invitation letter - clean	1.1	01 February 2013
Other: Patient Invitation letter - tracked changes	1.1	01 February 2013
Other: Patient Information letter - clean	1.1	01 February 2013
Other: Patient Information letter - tracked changes	1.1	01 February 2013
Other: Poster	1.1	01 February 2013
Other: Leaflet	1.1	01 February 2013
Other: details of the changes to the DILT1D Leaflet and Poster from V1.0 to V1.1		
Participant Consent Form: clean	2.1	01 February 2013
Participant Consent Form: tracked	2.1	01 February 2013
Participant Information Sheet: clean	2.1	01 February 2013
Participant Information Sheet: tracked	2.1	01 February 2013
Protocol	2.0	10 December 2012
REC application	115333/395070/1/65	21 December 2012
Response to Request for Further Information	Dr Frank Waldron-Lynch	04 February 2013

Statement of compliance

The Committee is constituted in accordance with the Governance Arrangements for Research Ethics Committees and complies fully with the Standard Operating Procedures for Research Ethics Committees in the UK.

After ethical review

<u>Reporting requirements</u>

The attached document *“After ethical review – guidance for researchers”* gives detailed guidance on reporting requirements for studies with a favourable opinion, including:

- Notifying substantial amendments
- Adding new sites and investigators
- Notification of serious breaches of the protocol
- Progress and safety reports
- Notifying the end of the study

The NRES website also provides guidance on these topics, which is updated in the light of changes in reporting requirements or procedures.

Feedback

You are invited to give your view of the service that you have received from the National Research Ethics Service and the application procedure. If you wish to make your views known please use the feedback form available on the website.

Further information is available at National Research Ethics Service website > After Review

13/EE/0020	Please quote this number on all correspondence

We are pleased to welcome researchers and R & D staff at our NRES committee members' training days – see details at http://www.hra.nhs.uk/hra-training/

With the Committee's best wishes for the success of this project.

Yours sincerely

pp NStorey

Mrs Carolyn Read
Chair

Email:Nicky.Storey@eoe.nhs.uk

Enclosures: "After ethical review – guidance for researchers"

Copy to: *Mr Stephen Kelleher, Cambridge University Hospitals NHS Foundation Trust*
