# Supplementary material for: Regulatory T Cell Responses in Participants with Type 1 Diabetes after a Single Dose of Interleukin-2: A Non-Randomised, Open Label, Adaptive Dose-Finding Trial
Source: PLoS Med. 2016 Oct 11;13(10):e1002139. doi: 10.1371/journal.pmed.1002139 (PMC5058548; doi:10.1371/journal.pmed.1002139)
Supplement: S4 Text — (PDF) [file pmed.1002139.s042.pdf]

NHS
Health Research Authority
NRES Committee East of England - Cambridge Central
The Old Chapel
Royal Standard Place
Nottingham
NG1 6FS

Telephone: 01223 597685
Facsimile: 01223 597645

05 March 2013

Dr Frank Waldron-Lynch
Cambridge Institute for Medical Research, University of Cambridge
Hills Road
Cambridge
CB2 0XY

Dear Dr Waldron-Lynch

**Study title:**	**Adaptive study of IL-2 dose on regulatory T cells in type 1 diabetes (DILT1D)**
**REC reference:**	**13/EE/0020**
**Protocol number:**	**DILT1D**
**IRAS project ID:**	**115333**

Thank you for your letter of 19th February 2013. I can confirm the REC has received the documents listed below and that these comply with the approval conditions detailed in our letter dated 18 February 2013

Documents received

The documents received were as follows:

Document	*Version*	*Date*
Covering Letter	Dr Frank Waldron-Lynch	19 February 2013
Other: Patient Invitation Letter - clean	1.2	19 February 2013
Other: Patient Invitation Letter - tracked changes	1.2	19 February 2013

Approved documents

The final list of approved documentation for the study is therefore as follows:

Document	*Version*	*Date*
Covering Letter	Dr Frank Waldron-Lynch	17 December 2012
Covering Letter	Dr Frank Waldron-Lynch	19 February 2013
Evidence of insurance or indemnity	University of Cambridge Finance Division	30 November 2012
GP/Consultant Information Sheets	1.0	06 December 2012

Investigator CV	Dr Frank Waldron-Lynch	10 December 2012
Other: Participant card	1.1	27 November 2012
Other: copy award letter from The Wellcome Trust	Dr Emma Hudson	04 June 2010
Other: Patient Information letter - clean	1.1	01 February 2013
Other: Patient Information letter - tracked changes	1.1	01 February 2013
Other: Poster	1.1	01 February 2013
Other: Leaflet	1.1	01 February 2013
Other: details of the changes to the DILT1D Leaflet and Poster from V1.0 to V1.1		
Other: Patient Invitation Letter - clean	1.2	19 February 2013
Other: Patient Invitation Letter - tracked changes	1.2	19 February 2013
Participant Consent Form: clean	2.1	01 February 2013
Participant Consent Form: tracked	2.1	01 February 2013
Participant Information Sheet: clean	2.1	01 February 2013
Participant Information Sheet: tracked	2.1	01 February 2013
Protocol	2.0	10 December 2012
REC application	115333/395070/1/65	21 December 2012
Response to Request for Further Information	Dr Frank Waldron-Lynch	04 February 2013

You should ensure that the sponsor has a copy of the final documentation for the study. It is the sponsor's responsibility to ensure that the documentation is made available to R&D offices at all participating sites.

**13/EE/0020**	**Please quote this number on all correspondence**

Yours sincerely

Ns Storey

Miss Nicky Storey
Committee Co-ordinator

E-mail: Nicky.Storey@eoe.nhs.uk

Copy to: Mr Stephen Kelleher, Cambridge University Hospitals NHS Foundation Trust
